# A review on commercial-scale high-value products that can be produced alongside cellulosic ethanol

**DOI:** 10.1186/s13068-019-1529-1

**Published:** 2019-10-08

**Authors:** Oscar Rosales-Calderon, Valdeir Arantes

**Affiliations:** Department of Biotechnology, Lorena School of Engineering, University of Sao Paulo, Estrada Municipal do Campinho, Lorena, SP CEP 12602-810 Brazil

**Keywords:** Cellulosic ethanol, Bioproducts, Commercial production, Biorefinery, Biofuel, Lignocellulose, Bio-based chemicals

## Abstract

The demand for fossil derivate fuels and chemicals has increased, augmenting concerns on climate change, global economic stability, and sustainability on fossil resources. Therefore, the production of fuels and chemicals from alternative and renewable resources has attracted considerable and growing attention. Ethanol is a promising biofuel that can reduce the consumption of gasoline in the transportation sector and related greenhouse gas (GHG) emissions. Lignocellulosic biomass is a promising feedstock to produce bioethanol (cellulosic ethanol) because of its abundance and low cost. Since the conversion of lignocellulose to ethanol is complex and expensive, the cellulosic ethanol price cannot compete with those of the fossil derivate fuels. A promising strategy to lower the production cost of cellulosic ethanol is developing a biorefinery which produces ethanol and other high-value chemicals from lignocellulose. The selection of such chemicals is difficult because there are hundreds of products that can be produced from lignocellulose. Multiple reviews and reports have described a small group of lignocellulose derivate compounds that have the potential to be commercialized. Some of these products are in the bench scale and require extensive research and time before they can be industrially produced. This review examines chemicals and materials with a Technology Readiness Level (TRL) of at least 8, which have reached a commercial scale and could be shortly or immediately integrated into a cellulosic ethanol process.

## Background

Over six decades ago, petroleum was the indisputable source of energy that kept the world working and growing. Nonetheless, at the beginning of the 1970s, the members of the Organization of Arab Petroleum Exporting Countries (OPEC) proclaimed an oil embargo aimed to control the production and, therefore, the price of petroleum [1]. In response, oil prices increased dramatically causing an “energy crisis” that awaked the interest in alternative fuels. Due to a serious surplus of crude oil caused by price controls and gasoline rationing, energy prices declined, causing the interest and support on alternative energy sources to decay [1, 2]. In addition to subsequent oil crises, the increasing evidence of the links between climate change and greenhouse gas (GHG) emissions has renewed the interest in alternative energy sources [3]. Thus, the emphasis today is to develop renewable energy sources that reduce our oil dependency and GHG emissions.

**Fig. 1:**
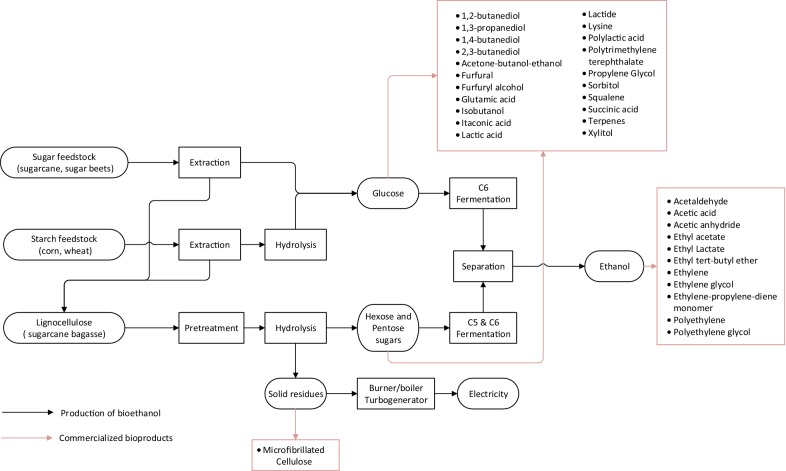
Process diagram to produce bioethanol from sugar and starch feedstocks, and lignocellulose including biochemicals and biomaterials with potential to be produced alongside bioethanol (red lines)

The transportation sector, which consumed 31.8% of the produced oil in 2017 [4], was responsible for 41.5% of the global CO_2_ emissions in 2016 [5]. Thus, a promising way of reducing our environmental impact and dependency on petroleum is through the substitution of gasoline and diesel with environmentally friendly fuels [6]. Ethanol produced from biomass, named bioethanol, is by far the most widely used biofuel in the transportation sector worldwide. As a result, the number of countries with renewable energy policies in the transportation sector increased from 56 in 2012 to 66 by 2015 [7]. Similarly, the annual world production of bioethanol increased from 13.0 billion gallons in 2007 to about 25.6 billion gallons in 2015 [7]. Despite these efforts, Brazil and the USA are the only countries that produce large quantities of bioethanol, 7.1 and 14.7 billion gallons of ethanol per year, respectively [8]. Bioethanol is currently produced from sugar- or starch-containing feedstocks. Sugar/starch derivate bioethanol is defined as first-generation (1G) bioethanol [9]. A general 1G bioethanol process is shown in Fig. 1. While the 1G bioethanol process is relatively simple, its main disadvantage is the high price of the sugar/starch feedstocks, which accounts for 40 to 70% of the total ethanol cost [10]. To achieve competitive costs and increase production, the supply of cheap raw materials is required. Cellulosic biomass, or lignocellulose, is considered the most promising feedstock for producing bioethanol, due to its availability, low cost, and the fact that it does not compete with food production as sugar/starch feedstocks do. In agreement with the 1G bioethanol definition, bioethanol produced from lignocellulose is named second-generation (2G) bioethanol or cellulosic ethanol [11].

Considering that one ton of glucan, galactan, or mannan yields 1.11 tons of six-carbon sugars, which could be fermented theoretically into 172.0 gallons of bioethanol, and that one ton of arabinan or xylan yields 1.14 tons of five-carbon sugars that could be fermented theoretically into 176.0 gallons of bioethanol, the theoretical global production of ethanol from lignocellulosic materials (rice straw, corn stover, wheat straw, pulp, etc.) can reach -442 billion liters per year [12]. Due to its complex composition (30–60% cellulose, 20–40% hemicellulose and 15–25% lignin), conversion of cellulosic materials to ethanol is more challenging than for sugar/starch-feedstocks [13]. Therefore, even when the cost of lignocellulose is lower than that of the sugar/starch crops, the production cost of cellulosic ethanol is too high to be competitive [14, 15]. Consequently, efforts to develop efficient and cost-effective technologies that reduce bioethanol’s production cost have been made in the last decades. After years of research and development, various cellulosic ethanol pilot and demonstration plants have started operations [16].

In 2012, Beta Renewables started up operations at the first industrial cellulosic ethanol plant in the world. By 2015, the 40 MMgy plant, located in Crescentino, Italy, was reported to operate on a daily basis, shipping cellulosic ethanol to Europe [17]. After this success, Beta Renewables was planning to build more cellulosic ethanol plants in India, USA, Brazil, and China. However, Beta Renewables was sold in 2018 to pay off debts from its bankrupt parent company, Mossi Ghisolfi Group [18]. DuPont started producing cellulosic ethanol at its 30-MMgy plant in Nevada, USA. With the merge of Dow Chemical and DuPont, questions about DuPont’s cellulosic ethanol investment raised. While DuPont continued building commercial relationships with feedstock growers and producing cellulosic ethanol [19], in 2017, DowDuPont announced that it intends to sell its cellulosic biofuels business and its first commercial cellulosic ethanol plant in Nevada, USA. The company found a buyer for the 30-million-gallon plant, VERBIO Vereinigte BioEnergie AG, a German company that produces renewable natural gas [20]. In 2015, Abengoa celebrated the opening of a 25 MMgy cellulosic ethanol plant in Hugoton, Kansas, USA. However, in 2016, after experimenting financial difficulties, Abengoa declared its cellulosic bioethanol plant in bankruptcy [21]. In contrast, in 2014, Raizen started up operations at its 40 MMgy cellulosic ethanol plant [22]. Raizen’s estimated minimum ethanol selling price ($2.17 per gallon) is the lowest among the current operating cellulosic plants [23]. While Raizen reported plans to export cellulosic ethanol to Europe, the company announced reductions in its cellulosic ethanol investment due to low gasoline prices [24]. In 2014, GranBio started up a cellulosic ethanol plant with a capacity of 20 MMgy in Brazil. However, the plant suspended operations in 2016 due to technical difficulties in the pretreatment stage and resumed operations in 2019 [25, 26]. In 2017, Enviral (Slovakia) acquired a license to use Clariant’s sunliquid technology (Switzerland) in a commercial-scale plant for the production of ethanol from agricultural residues. The planned plant will be integrated into the Enviral’s facility at Leopoldov, Slovakia, and will have an annual production capacity of 50 ktons (15 million gallons per year, MMgy) [27]. In 2014, POET-DSM Advanced Biofuels, a 50/50 joint venture between Royal DSM (Netherlands) and POET, LLC (USA), opened its Project Liberty facility in Emmetsburg, Iowa, USA. The cellulosic ethanol facility was set to produce 20 MMgy of ethanol and then ramp up to 25 MMgy [28]. In 2017, the company achieved a major breakthrough by announcing that Project Liberty was running pretreatment at 80 percent uptime. Moreover, POET-DSM announced the construction of an on-site enzyme manufacturing facility and ramped up biomass purchasing in anticipation of increasing production levels for 2018 [29].

Regardless of all these efforts, the global new investment in biofuels continues to decline. In 2015, the global new investment in biofuels power capacity fell by 35%, in relation to 2014, to USD 3.1 billion [7]. Thus, to boost the investment on cellulosic ethanol, technologies that reduce the production costs must be developed and industrially demonstrated. The biorefinery concept, in which biomass is converted to biochemicals and biomaterials, such as benzene, microfibrillated cellulose, toluene, xylene, styrene, or cumene [30], is a promising strategy to reduce production costs. Even so, the large number of possible combinations of feedstock, pretreatment options, conversion technologies, and downstream processes, makes difficult the evaluation of these technologies. Various authors have reviewed promising chemicals that can be produced from lignocellulose. Nonetheless, most of the technologies behind these chemicals are under development and their commercial feasibility is uncertain. Thus, this review focuses on the compelling analysis of commodity chemicals that can be produced alongside cellulosic ethanol and that are at a manufacturing level.

## Production of cellulosic ethanol

### Feedstock

Biomass is a renewable resource that is appropriate to produce ethanol and chemicals. Lignocellulose is the most promising biomass feedstock because of its availability and lowcost [31, 32]. For example, the sugarcane and corn price have been reported to be $60.9/ton [33] and $185.9/ton [34], while sugarcane bagasse and corn stover prices have been estimated to be $36.38/ton [35] and $58.50/ton [36], respectively. In contrast to the production of bioethanol from starch, cellulosic biomass is not used as a food source. The primary drivers of ethanol prices are the cost of corn grain and the gasoline prices. In the past 10 years, ethanol prices have fluctuated in correlation with gasoline or corn grain prices. When corn grain was relatively inexpensive and petroleum prices were increasing, ethanol was traded based on gasoline prices. As ethanol began to consume a larger percentage of corn grain production, its price increasingly moved in sync with corn grain prices. The correlation between corn grain and ethanol prices is expected to decline once substantial volumes are produced from cellulosic feedstock [37]. Despite these advantages, the complex structure of lignocellulose makes its processing challenging and expensive. For example, the price of sugars was reported to be $282.5/ton [38], while minimum selling price of sugars produced from corn stover was estimated to $587.3/ton [35].

Examples of lignocellulose include agricultural wastes (corn stover, wheat or rice straw), sugarcane bagasse, wood (hardwood or softwood), grass, municipal waste, and dedicated energy crops (miscanthus and switchgrass) [39]. Lignocellulose is composed of lignin, polysaccharides, such as cellulose and hemicelluloses, and pectin, proteins, ash, salts, and minerals [40]. Cellulose, the main component, consists of chains of glucose linked by β-1,4 linkages. These chains form crystalline microfibrils, which are highly recalcitrant to degradation, and amorphous domains, which are easily decomposed [41, 42]. Unlike cellulose, hemicellulose is not chemically homogeneous as it is composed of polymerized monosaccharides (glucose, mannose, galactose, xylose, arabinose, 4-*O*-methyl glucuronic acid, and galacturonic acid residues). Hemicelluloses, the second most common polysaccharides in nature, represent about 20–35% of lignocellulosic biomass [43]. Xylan is the most abundant hemicellulose in lignocellulosic biomass and contains mainly β-d-xylopyranosyl residues linked by β-1,4-glycosidic bonds [44]. Lignin, the third major component, acts as a binder between plant cells, and it is strongly resistant to biological degradation. Lignin is an aromatic macromolecule with a complex and diverse structure, which monomer units appear to repeat randomly [45]. The proportion of these three components in lignocellulose varies substantially depending on the type of biomass and harvest time [40, 46–50].

In contrast to the production of bioethanol from starch, cellulosic biomass is not used as food source. The primary drivers of ethanol prices are the cost of corn grain and the gasoline prices. In the past 10 years, ethanol prices have fluctuated in correlation with gasoline or corn grain prices. When corn grain was relatively inexpensive and petroleum prices were increasing, ethanol was traded based on gasoline prices. As ethanol began to consume a larger percentage of corn grain production, its price increasingly moved in sync with corn grain prices. The correlation between corn grain and ethanol prices is expected to decline once substantial volumes are produced from cellulosic feedstock [37].

### Cellulosic ethanol process

The conversion of lignocellulose to ethanol is challenging, mainly due to the resistant nature of lignin to degradation, the inefficient breakdown of cellulose and hemicellulose, the variety of sugars released from the carbohydrate polymers, and the cost for storage, transport, and collection of low-density lignocellulosic feedstock [51]. The production of lignocellulosic ethanol starts with the collection and transportation of lignocellulosic feedstock to the plant site, where, depending on the feedstock, it is fed to a preprocessing step (e.g., grinding, milling) to the feedstock particle size [52]. As shown in Fig. 1, bioethanol production from lignocellulose typically comprises four major steps: (1) a pretreatment step to make polysaccharides more accessible, (2) an enzymatic hydrolysis process to break down polysaccharides to simple sugars, (3) a fermentation step where a microorganism ferments sugars into ethanol, and (4) a separation stage to obtain fuel grade ethanol [53].

Within the cellulosic ethanol process, the conversion of biomass to sugars is the main barrier to achieve cost-effective production of cellulosic ethanol. The polysaccharides are buried within ordered and tightly packed cellulose microfibrils, embedded in a matrix of hemicelluloses and lignin. Thus, the one major bottleneck to efficient enzymatic hydrolysis is the limited access of enzymes to the polysaccharides [54, 55]. In addition, lignin non-specifically adsorbs and inhibits cellulases, the enzymes in charge of depolymerizing cellulose to glucose [49, 56–59]. Thus, a pretreatment stage which exposes cellulose, increasing access to enzymes, is needed. Multiple pretreatment technologies, such as steam explosion (SE), dilute sulfuric acid (DA), organosolv, ammonia fiber expansion (AFEX), and liquid hot water (LHW), have been developed in the past years [60]. Organosolv pretreatment uses organic solvent normally at 100–200 °C for short period to separate cellulose and produce unaltered lignin [61]. SE and DA pretreatments effectively hydrolyze a large portion of hemicellulose, as well as disrupt lignin, while increasing cellulose digestibility. The AFEX process pretreats biomass with anhydrous liquid ammonia at high pressure and moderate to high temperatures. In the AFEX process, the pressure is rapidly released, disrupting the biomass structure and resulting in the partial decrystallization of cellulose. The effectiveness of the pretreatment technologies and enzymatic hydrolysis depends on the type of lignocellulose and operating conditions used. For example, the rate and extent of the enzymatic hydrolysis of pretreated lignocellulose decline with increasing pretreatment slurry concentration [62, 63].

The enzymatic hydrolysis of lignocellulose is the main barrier to produce feasible 2G bioethanol. Enzymatic hydrolysis is advantageous when compared to acid hydrolysis, the chemical alternative, as it requires less energy, milder operating conditions, and it is less corrosive and toxic [64–66]. During enzymatic hydrolysis, cellulase and hemicellulase enzymes depolymerize cellulose and hemicellulose to hexoses (mannose, glucose, and galactose) and pentoses (xylose and arabinose), respectively. The three major groups of cellulases involved in the hydrolysis reaction are as follows: endoglucanase (*endo* 1,4-d-glucanase or E.C. 3.2.1.4) which attacks randomly regions of low crystallinity to create free chain-ends, exoglucanase or cellobiohydrolase (1,4-β-d glucan cellobiohydrolase or E.C. 3.2.1.91c), which releases cellobiose from the free chain-ends of crystalline regions, and β-glucosidase (E.C. 3.2.1.21) that hydrolyzes the released cellobiose to glucose [49, 64, 65, 67–69]. Due to hemicellulose complexity and the large number of enzymes required to hydrolyze it, synergy studies have only identified a few interactions between hemicellulases and substrates [40]. *Endo*-xylanases (EX) cleave the xylan backbone at internal β-1,4 xylosidic bonds, while β-xylosidases hydrolyze short xylooligomers to xylose. Past studies have evaluated the hydrolytic efficiency of cellulases produced by various microorganisms [70–77]. Cellulases produced from *Trichoderma reesei* and *Aspergillus niger* are the most extensively studied [69, 78–87]. Multiple compounds formed or released during the pretreatment and hydrolysis stage, such as 5-HMF and vanillin, inhibit the enzymatic hydrolysis. Moreover, a factorial design and analysis of variance on the enzymatic hydrolysis of dilute acid pretreated corn stover showed that high sugar concentrations (130 g/L) have a major inhibitor effect on the enzymatic hydrolysis [88]. To improve the hydrolysis yield, the private sector and academia have studied the supplementation of chemicals, enzymes, and proteins to boost cellulases performance and inhibitors resistance [89–92]. Some of the commercial enzymatic preparations released over the years are: Spezyme^®^ CP [57, 82, 93–96], Acellerase™ 1000 [82, 95, 97–100], Acellerase™ 1500 [101–105], and Acellerase™ DUET [106, 107] from Genencor, and Celluclast^®^ 1.5L [49, 79, 108–115], Novozyme 188 [87, 113, 114, 116–118], Cellic^®^ CTec2 [79, 113, 119, 120], and Cellic^®^ CTec3 [121, 122] from Novozymes. Recent studies have focused on non-hydrolytic enzymes, such as polysaccharide monooxygenases (LPMOs), which enhance hydrolysis by reducing enzyme supplementation [123]. LPMOs are copper-dependent enzymes capable of breaking glycosidic bonds in polysaccharides, such as cellulose, xyloglucan, glucomannan, xylan, starch, and chitin [124]. LPMOs are believed to oxidize crystalline cellulose, creating more reducing/non-reducing ends for cellulases to attack [125]. Replacing a fraction of supplemented cellulases with LPMOs has been shown to increase the hydrolysis yield of steam-exploded birch by 25–30% [126]. Despite the apparent advantages of LPMOs, aldonic acids which are produced during the oxidation of polysaccharides by LPMOs can inhibit enzymes and microbes [127]. More research is needed to determine if LPMOs are advantageous for the production of bioethanol. The enzymatic hydrolysis process has been scaled up and used in the industrial-scale plants operated by Beta Renewables, Abengoa, POET, Raizen, GranBio, and DuPont [17, 19, 21, 128].

The next stage in the cellulosic ethanol process is the fermentation stage, in which sugars produced during enzymatic hydrolysis or solubilized during the pretreatment stage are converted to ethanol by microorganisms. The lack of organism that efficiently converts all the hexoses (glucose, galactose, and mannose) and pentoses sugars (xylose and arabinose) to ethanol is another obstacle to the viable production of cellulosic ethanol. Hence, fermentation research has focused on identifying wild or genetically engineered yeast and bacteria capable of fermenting both hexoses and pentoses at productive yields [129–134]. Despite the promising results obtained from engineered organisms, there are issues that need to be addressed, for example, incomplete pentose conversion, low reaction rates, and low microorganism tolerance to ethanol and inhibition by compounds produced during pretreatment [39, 135].

In the final stage of the process, ethanol is separated and concentrated to obtain fuel grade ethanol. Ethanol can be recovered from the fermentation broth by distillation, adsorption, or filtration using an entrainer, molecular sieves, or membranes [136–139]. The solid residue obtained from the distillation stage is normally proposed to be used as a solid fuel to produce heat and steam for the process [35, 140, 141]. However, these residues may be suitable to produce more valuable products [142, 143].

### Production and impact of inhibitors

The enzymatic hydrolysis and fermentation reactions can be inhibited by several compounds. Inhibitors can be naturally present in biomass or can be formed during pretreatment. Plants deploy inhibitors to protect themselves against pathogens that utilize cellulases to gain access to the plant cells. Some of these inhibitors include hemicellulose’s hydrolysis products, pectin and its hydrolysis products (uronic acids), and phenolic compounds (gallic acid, trans-cinnamic acid, 4-hydroxybenzoic acid, syringaldehyde, and vanillin) [144]. For example, acetic acid is formed from the hydrolysis of acetyl groups in hemicellulose, while formic and levulinic acids are generated during the degradation of sugars. Similarly, furfural and 5-hydroxymethylfurfural (5-HMF) are formed by dehydration of pentoses and hexoses, respectively, under thermal and acidic conditions [81]. Arora et al. [145] reported that furfural and 5-HMF reduce glucose recovery by 5 to 81% after enzymatic hydrolysis. Production of inhibitors also varies depending on the pretreatment applied, for example, acetic acid, furfural, and 5-HMF are produced in the DA pretreatment, but are not generated by the AFEX pretreatment [146]. In accordance, with the above, van der Pol et al. [147] reported that the synergetic effect of coumaric acid, formic acid, and acetic acid, formed during the alkaline pretreated lignocellulose, is a key inhibitory parameter in the enzymatic hydrolysis, while furfural is a key inhibitor formed in the acid pretreatment of lignocellulose.

The strength of the inhibition or deactivation effect depends on the type of enzyme, the microorganism from which the enzyme is derived, and the type and concentration of the inhibitory compounds present. Kumar and Wyman reported that xylobiose and higher xylooligomers inhibit the enzymatic hydrolysis, while xylose, xylobiose, and xylotriose presented progressively greater effects on hydrolysis rates [90]. Consistently, Ximenes et al. [144] reported that pectin, xylooligosaccharides, and xylose significantly inhibit cellulose hydrolysis, while vanillin was reported to have the most significant inhibitory effect. Syringaldehyde and trans-cinnamic acid moderately inhibit hydrolysis, while 4-hydroxybenzoic acid was the least inhibitory compound. In some cases, tannic, gallic, hydroxy-cinnamic, 4-hydroxybenzoic acids, and vanillin have been reported to reduce the cellulose enzymatic hydrolysis yield by 20 to 80% [144]. In the fermentation stage, ethanol yield and volumetric productivity decrease with the increasing concentrations of acetic acid, formic acid, and levulinic acid [148]. In contrast, furfural and 5-HMF have been shown to decrease the volumetric productivity, but not to influence the final yield of ethanol [148].

Multiple approaches have been proposed to reduce the detrimental effect of inhibitors. One way to reduce the detrimental impact of inhibitors in the hydrolysis and fermentation reactions is detoxification of the hydrolysates [149]. Detoxification processes involve the treatment of hydrolysates with alkalis, sulfites, laccases, etc., or the adsorption of inhibitors onto carriers like activated carbon and other synthetic resins [150]. For example, Christopher et al. [151] evaluated the detoxification of acid pretreatment liquor using adsorbent (ADS 400 and ADS 800) and ion-exchange (A-27MP and A-72MP) resins. Of the tested resins, ADS 800 removed 85% and 60% of furfural and HMF, respectively. Furthermore, ADS 800 was reused for up to six cycles after regeneration without losing its adsorption properties. A major downside of implementing detoxification processes is the related increase in capital and operating costs. Thus, an ideal pretreatment should work on multiple feedstocks, increase biomass digestibility, generate no or minimal inhibitors, and be energy and chemical efficient [39, 105, 152]. Although some technologies closely meet these criteria, rigorous analysis that ponders the pretreatment impact on bioethanol’s downstream stages and production costs is needed to demonstrate their commercial viability [39, 153].

Despite the negative effect of lignin on the bioethanol production, lignin is a valuable material that can be used as a low-grade fuel, raw material to produce carbon fiber [154], precursor for antidiabetic drugs [155], and reinforced material for abrasive tools [156]. High-purity lignin, with ash content < 0.1% and sulfur content < 1%, can be obtained using supercritical treatment, deep eutectic solvents, or ethanol-based organosolv process, such as those demonstrated by Lignol Innovations in Canada. Lignol Innovations has been acquired by Fibria Cellulose S.A., a Brazilian company, and its technology is underdeveloped [157]. Another promising product derivate from lignocellulose is bio-oil. In 2012, Fibria paid US$20 million to become a partner of Ensyn, a USA company that owns the technology to produce bio-oil [158]. Ensyn patented the RTP^®^ technology, a commercial thermal process that produces bio-oil from cellulosic biomass. The bio-oil is used for heating and cooling purposes, and as a refinery feedstock to produce renewable “drop-in” gasoline, diesel, food flavorings, and fragrances. Bio-oil yields are typically 70 to 75 wt% from dried wood residues [159]. In 2016, Fibria reported that it was planning to build a bio-oil plant in Jacareí, São Paulo, or Aracruz, Espírito Santo, Brazil, where the company operates various pulp mills [158]. In 2017, Fibria performed industrial testing of bio-oil processing and continued to fine-tune the product [160].

## Opportunities to produce bio-based chemicals alongside cellulosic ethanol

The global production of bio-based chemicals and polymers is estimated to be around 50 million tonnes per year (mtpy) [161]. However, most chemicals and polymers are still produced from petroleum. The commercial production of bioproducts has been restricted due to the petroleum’s low price and optimized processes. The fluctuation in oil prices and the increasing demand for environmentally friendly products has boosted the interest in chemicals and polymers derived from renewable resources. Moreover, co-production of chemicals, materials, and food may generate the necessary added value to commercialize the cellulosic ethanol.

To organize and analyze the hundreds of chemicals and polymers that can be obtained from cellulosic biomass, recent studies have used a classification method based on biorefinery platforms (e.g., carbohydrates, syngas, lignin, pyrolytic liquid) [161, 162]. These platforms are key intermediates between raw materials and final products, and can link different processes and biorefineries. In the oil and gas industry, all produced chemicals and polymers are derived from a small number of key building blocks: methanol, ethylene, propylene, butadiene, benzene, toluene and xylene [163]. Likewise, in 2004, the US Department of Energy issued a report listing 12 promising bio-based chemicals from a sugar-based platform. These compounds were considered the potential building blocks for the future [163]. Later in 2010, this list was reviewed and updated [164]. In 2012, the International Energy Agency (IEA) Bioenergy published a report that highlighted bio-based chemicals with immediate potential for commercialization. Listed products were selected based on their strong market growth, industrial investment, and demonstration programmes [161]. In a report for the European Commission Directorate-General for Energy, a more limited (94) number of bio-based products, either in the development pipeline stage with supporting industry interest, or already commercialized with the potential to grow, were identified [162]. In 2007, the US Department of Energy published a report evaluating opportunities to convert lignin into power, macromolecules, or aromatics, such as methanol, cyclohexane, styrene, phenol among others [165]. Since lignin constitutes up to 30% of the weight and 40% of the fuel value of biomass, lignin represents a valuable opportunity to increase the commercial viability of a biorefinery. Agrawal et al. [154], presented an insight into possible products and uses of lignin (e.g., phenol, guaiacol, vanillin, and ethylated kraft lignin). However, technologies to convert lignin to macromolecules and aromatic chemicals are under development and represent long-term opportunities. Thus, only the use of lignin as power or fuel represents a near-term opportunity. While more platforms, such as pyrolysis oil [166, 167], syngas [168–171], or algae [172], are being developed, this study will focus on matured technologies for producing bio-based chemicals that can be integrated into a cellulosic ethanol process. Therefore, we will focus on bio-based chemicals with a Technology Readiness Level (TRL) of at least 8, which are in the manufacturing level [162].

### 1,2-Butanediol

**Fig. 2:**
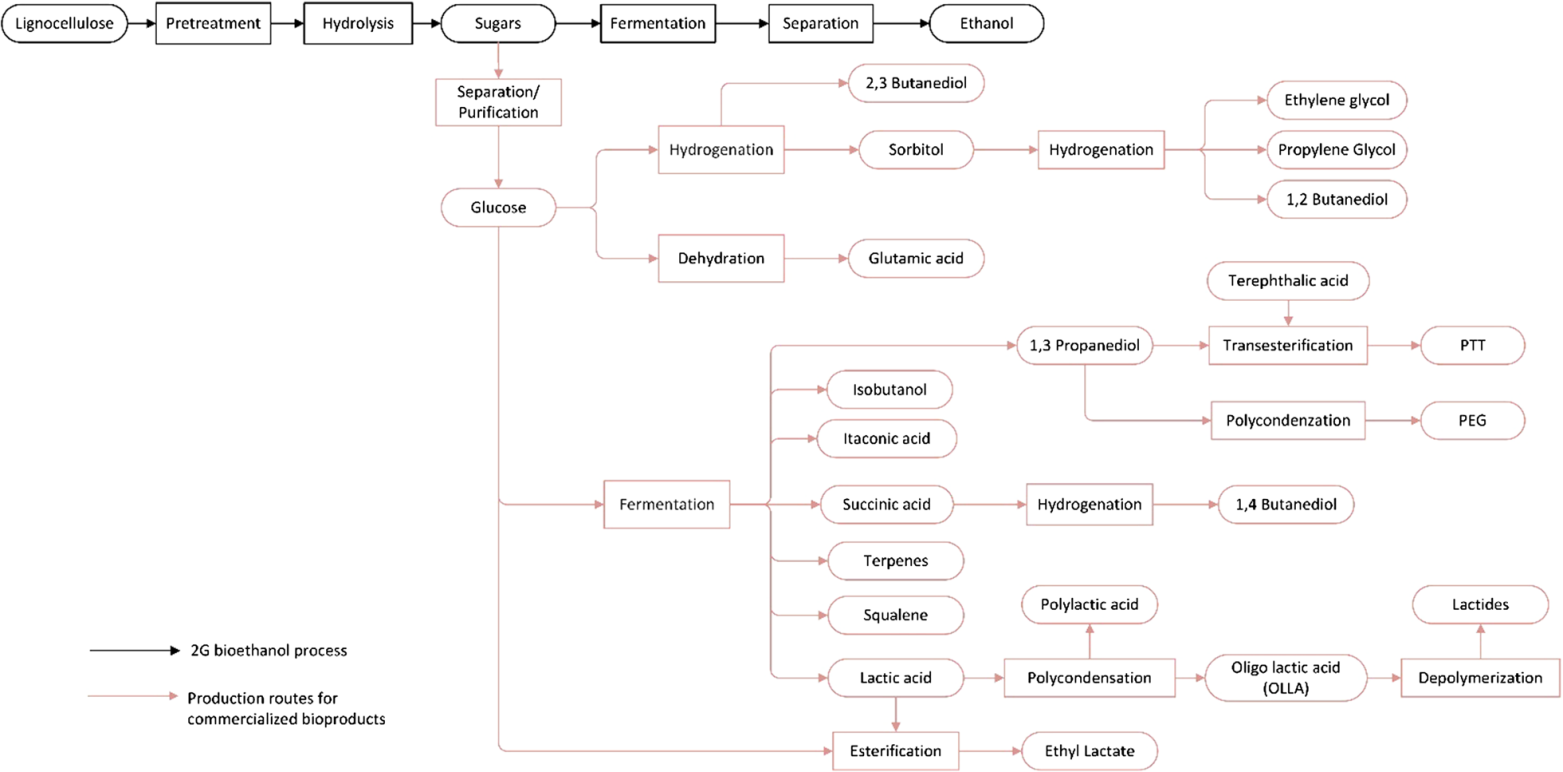
Process diagram for the production of second-generation bioethanol from lignocellulose. Production routes to produce biochemicals from glucose that are industrially produced (red lines)

The compound 1,2-butanediol (1,2-BDO) or 1,2-butylene glycol, can react with a dicarboxylic acid, for example, phthalic acid, or adipic acid, for use as a polyester polyol or a plasticizer. It may be used to produce adhesive resins or as a solvent, coolant, refrigerant, hydraulic fluid, or fine chemical raw material [173]. It is estimated that the butanediol market is approximately $4 billion per year (2016) with a volume of 1.5 mtpy, which is expected to grow to more than $7.5 billion per year and 2.7 mtpy by 2020 [174]. Traditionally, 1,2-BDO is manufactured through the catalytic or steam cracking of gas (ethane, propane, butane) and/or naphtha [175]. In the catalytic cracking route, high-boiling-point fractions of hydrocarbons are upgraded to high octane gasoline. In contrast, steam cracking converts a variety of hydrocarbon feedstocks to light olefins and aromatic products. A third pathway to produce 1,2-BDO uses sorbitol (Fig. 2), generated from corn starch sugars, to produce polyol products. Sorbitol can be hydrogenated to glycols using a catalyst. Gu et al. [176] used a series of Ru catalysts using activated carbon and carbon nanotubes as supports to compare their activities and selectivity in sorbitol hydrogenolysis. Reported yields, on a carbon basis, ranged between 24.8 and 34.6% for propylene glycol (PG), 16.7–25.6% for ethylene glycol (EG), 4.3–8.7% for glycerol, and 0.4–1.4% for 1,2-BDO, depending on the catalyst used [176]. By adding cerium to Ni/Al_2_O_3_ catalysts, Ye et al. [177] enhanced the conversion of sorbitol to glycols by up to 40.2%. The selectivity of 1,2-BDO achieved by the addition of cerium into the catalysts ranged from 0 to 2.9% (carbon basis). The conversion yield and selectivity to sorbitol varied depending on the amount of cerium added and the catalyst preparation method used.

In 2004, Global Biochem (HK, China) and International Polyol Chemicals, Inc. (IPCI), from OR, USA, built and started operation of a plant capable of producing 2200 tonnes of EG, 5200 tonnes of 1,2-BDO, and other polyols from 10,000 tonnes of sorbitol per year [178]. In recent years, Global Biochem is producing a number of corn-based polyols: EG, 1,2-PG, 1,2-BDO, 2,3-butanediol (2,3-BDO), and bio-based resins at the province of Jilin, China [179]. Due to poor market condition, Global Biochem suspended most of its polyols production in March 2014, but continued to sell its polyol chemicals inventory. Global Biochem announced that by making a provision of polyol chemicals in 2015, the polyol chemicals segment recorded gross profit of approximately $0.5 million (2015: $2.2 million), with a gross profit margin of 79.6% (2015: 27.7%) during 2016 [180]. The technology to generate 1,2-BDO from sorbitol is technically mature, and while it is possible to enhance the hydrogenolysis selectivity and yield via catalyst optimization, the production of 1,2-BDO is limited by the price of fossil-based polyols and market demand. Production of 1,2-BDO alongside cellulosic ethanol is technically possible. However, the introduction of a process to produce sorbitol from hydrolyzed sugars is required. Alternatively, 1G and 2G bioethanol could be co-produced in a biorefinery arrangement, as shown in Fig. 2, allowing the use of the glucose stream, generated from the starch/sugar biomass, to produce sorbitol and 1,2-BDO.

### 1,3-Propanediol

Also known as trimethylene glycol, 1,3-propanediol (1,3-PDO) has promising properties for synthetic reactions, particularly as a monomer for the polycondensation reaction to produce polyesters, polyethers, and polyurethanes. The global value of the 1,3-PDO market was USD 310 million in 2014 and it is likely to reach USD 620 million by 2021 as shown in Table 1 [181]. Global 1,3 propanediol market demand was 146 kilo tons in 2014 and is expected to reach 225.9 kilo tons by 2022 [182, 183]. In 1995–1996, after Shell and DuPont commercialized a 1,3-PDO-based polyester (polytrimethylene terephthalate, PTT), 1,3-PDO evolved from a fine to a bulk polymer [184]. DuPont produced 1,3-PDO through the pro-Degussa technology, at Wesseling, Germany, which uses acrolein obtained via the catalytic oxidation of propylene [184, 185]. Acrolein is hydrated at moderate temperature and pressure to 3-hydroxypropionaldehyde which is then hydrogenated to 1,3-PDO over a rubidium catalyst under high pressure (90 bar) [186]. In contrast, Shell uses ethylene oxide obtained via oxidation of ethylene. Ethylene oxide is transformed in a hydroformylation process to 3-hydroxypropanal, like DuPont’s process, but at very high pressure (150 bar). 3-Hydroxypropanal is extracted and subjected to hydrogenation using nickel as a catalyst, under high pressure [187]. In DuPont’s process, the yield does not exceed 65%, while Shell’s process achieves a yield of 80%. As ethylene oxide has a lower price than acrolein, Shell’s process is economically more favorable. Prices for 1,3-PDO are suggested to be around $1.76/kg (2008) [188] and, $2.20/kg (2005) [189], and $1.45/kg [190].

**Table 1:** Summary of the chemicals and materials analysed

Compound	Classic production route	Bio-based route	Price (per ton)	Market (per year)	Market volume (tonnes per year)	Uses	Challenges for integration
1,2-Butanediol	Catalytic or steam cracking of gas (ethane, propane, butane) and/or naphtha [175]	Hydrogenation of sorbitol [176]	$2666 [174]	$4 billion [174]	1.5 million [174]	Production of adhesive resins or as a solvent, coolant, refrigerant, hydraulic fluid, or fine chemical raw material. [173]	Relies on the successful production sorbitol
1,3-Propanediol	Pro Degussa’s technology [184, 185], hydrogenation of 3-hydroxypropionaldehyde [186]	Fermentation of sugars [198, 199, 203]	$1450 [190]	$310 million [181]	146,000 [182]	Production of polyesters, polyethers, and polyurethanes [182, 183]	Separation and purification of the cellulosic sugars stream Optimization of microorganism
1,4-Butanediol	Reppe process [198], hydrogenation, and hydrolysis [206, 208]	Hydrogenation of SA [209], and fermentation of sugars [210]	$2660 [205]	$5690 million [538]	2 million [205]	Manufacture of polymers, solvents, and chemicals. [206, 207]	Relies on SA Optimization of microorganism and catalysts
2,3-Butanediol	Chlorohydrination and hydrolysis [539]	Catalytic hydrogenation [223] and fermentation of sugars [225–229]	$10,000 to 50,000 [222]	$43 billion [220, 221]	32 million [220, 221]	Production of printing inks, perfumes, fumigants, moistening and softening agents [219]	Separation and purification of the cellulosic sugars stream
Acetaldehyde	Dehydrogenation or oxidation of ethanol, addition of water to acetylene, partial oxidation of hydrocarbons, and direct oxidation of ethylene [251–253]	Oxidation of ethanol [252, 255]	$1005 [540]	$1.26 billion [250]	1.28 million [541]	Manufacture of acetic acid, perfumes, dyes, and drugs, as a flavoring agent and as an intermediate in the metabolism of alcohol [249]	Competitive prices against fossil-based equivalent
Acetic acid	Carbonylation with Rh-catalyzed Monsanto or Ir-catalyzed Cativa process [258]	Catalytic oxidation of ethanol [261, 263], fermentation of ethanol or sugars [264]	$617 [162]	$8373 million [162]	8.3 million [161]	Applications include: foam rubber, wood gluing, emulsifiers, cement coatings, and desalination membranes [142]	Separation and purification of the cellulosic sugars stream Competitive prices against fossil-based equivalent
Acetic anhydride	AcOH dehydration [270], acetone cracking [271], acetaldehyde oxidation [272], and methyl acetate carbonylation [273]	AcOH dehydration [270]	$797 [542]	$635.9 million [543]	2.7 million [269]	Use in the preparation of modified food starch and acetylation of monoglycerides, and in the manufacture of pesticides and herbicides [268]	Relies on AcOH Competitive prices against fossil-based equivalent
Acetone	Thermal decomposition of calcium acetate, dehydrogenation of isopropyl alcohol or cumene oxidation [264, 277]	Fermentation of sugars [277] and ABE process [264, 281]	$1210 [205]	$7700 million [162]	6.1 million [544]	Production of acrylic plastics, signs, lighting fixtures and displays, and Bisphenol A (BPA), and as a solvent in multiple products, such as paints, cleaning fluids, and adhesives [276]	Produced alongside ethanol and butanol Addressed inhibitors and toxic compounds
Butanol	Oxo reaction of propylene [280]	ABE process [264, 281]	$1463 [205]	$4200 million [162]	3.4 million [545]	Use in the manufacture of adhesives, sealant chemical, paint additives, coating additives, plasticizer, and cleaning products [280]	Produced alongside ethanol and acetone Detailed market analyses needed to define competitiveness against ethanol
Ethanol	Direct or indirect catalytic hydration of ethylene, homologation of methanol, and carbonylation of methanol and methyl acetate [546]	Fermentation of starch, sugar, and other carbohydrates [130]	$514 [547]	$64.52 billion [548]	80.88 million [549]	Manufacture of adhesives and sealant chemicals, beverage ingredients, food products and use as fuels and fuel additives [546]	Competitive prices against fossil-based equivalent Elevated production costs
Ethyl acetate	Fisher esterification, dehydrogenation of ethanol, and Tischenko reaction [302]	Dehydrogenation of ethanol [303]	$1434–1507 [301]	$1.33 billion [550]	3.2 million [300]	Used in the production of inks, adhesives, car care chemicals, plastic, and as a synthetic fruit essence, flavour and perfume in the food industry [299]	Competitive prices against fossil-based equivalent
Ethyl Lactate	Esterification of LA with fossil based ethanol [312], and esterification of ammonium lactate [314]	Esterification of LA with bio-based ethanol [312]	$3300–4400 [312]	$81 million [551]	1.2 million [161]	Potential to replace conventional petroleum-based solvents such as toluene, methyl ethyl ketone, and *N*-methyl-pyrrolidone [198]	Relies on LA and ethanol High dependency on demand and price of raw materials
Ethyl tert-butyl ether	Catalytic reaction of fossil based isobutene and ethanol [324, 325]	Catalytic reaction of fossil based isobutene and bioethanol [324, 325] [327]	$856–906 [552]	$2.13 billion [553]	3 million [553]	Use as gasoline additive [321]	Relies on isobutene and ethanol Future demand Competitive prices against fossil-based equivalent
Ethylene	Steam cracking of hydrocarbons [142]	Dehydration of ethanol [328]	$1370 [162, 205]	$140,000–203,000 million [162, 205]	146.5 million [554]	Use to manufacture plastics, e.g., HDPE, LDPE, LLDPE, PVC, and PET [161]	Relies on ethanol Depends on the ethanol price and stock
Ethylene glycol	Hydrolysis of ethylene oxide. [142, 336]	Hydrogenation of sorbitol [337]	$1144 [205]	$28.58 billion [555]	34.8 million [555]	Manufacture of antifreeze, hydraulic brake fluids, industrial humectants, printer’s inks, and in the synthesis of safety explosives, plasticizers, synthetic fibers [335], and MEG [161]	Relies on sorbitol Competes against fossil-based equivalent
Ethylene propylene diene monomer	From fossil based ethylene using Ziegler–Natta olefin polymerization [346]	From bio-based ethylene using Ziegler–Natta olefin polymerization [346]	$2000 [556]	$4203 million [556]	1.32 million [557]	Use in the automotive, construction industry, and in the manufacture of oil additives [342]	Relies on ethylene Requires favorable rubber market
Furfural	–	Acid hydrolysis of pentosan [353]	$2200 [351]	$625.5 million [287]	270,000 [198]	Apply in the production of specialist adhesives, and as flavor compound [350], it is a key bio-based platform chemical that can be used to replace oil-based chemicals [142]	High degradation of cellulose during furfural processing Further R&D needed
Furfuryl alcohol	–	Hydrogenation of furfural [162]	$1500 [558]	$376.9 million [558]	300,000 [559]	Use as an additive or solvent in the production of resins, as a chemical intermediate to manufacture lysine, vitamin C, lubricants, and dispersing agents [362]	Relies on furfural Successful separation of pentose- and hexose-sugars
Glutamic acid	–	Fermentation of sugars [142, 373]	$1400 [560]	$8.5 million [561]	200,000 [142]	Use as thickener, humectant, cryoprotectant, drug carrier, biodegradable fibers, highly water absorbable hydrogels, biopolymer flocculants, and animal feed additives [372]	Volatility of corn and coal prices Separation and purification of the cellulosic sugars stream
Isobutanol	Hydrogenated of butyraldehyde [386], LP OxoSM Process [387],, and Reppe carbonylation [387]	Fermentation of sugars, [389] and consolidated bioprocessing (CBP) [392]	$1530 [205]	$775.0 million [562]	500,000 [205]	Precursor of various isobutyl esters, chemical intermediate, pharmaceuticals, and automotive paint cleaner additive [383], was well as being use as a fuel [384]	Separation and purification of the cellulosic sugars stream
Itaconic acid	Distillation of citric acid [398]	Fermentation of sugars [398]	$1900 [162]	$79.0 million [162]	40,000 [563]	Production of lubricant additives, surface active agents, dye intermediates, plastics, synthetic rubber, and MMA [162]	Niche market with low demand Downstream conversion to MMA not commercial Commercial production stopped
Lactic acid	Hydrolysis of lactonitrile by H2SO4 or by HCl [408]	Fermentation of sucrose [408]	$1300–2300 [407]	$2.22 billion [564]	120,000 [407]	Apply in the food and beverage sector as a preservative and pH adjusting agent, and as a starting material in the production of lactate ester [161]	Technical barriers similar to 2G ethanol. Separation and purification of the cellulosic sugars stream
Lactide	Depolymerization of OLLA. [418]		–	$781.81 million [565]	–	Use as an additive for the conservation of milk and meat-based products, as a pH regulator for tofu, soybeans, and dairy products, and as a combustion improvement agent for coal and oil [416]	Relies on LA
Lysine	Fermentation of sugars [161, 426, 427]		$1200 [566, 567]	$745.2 million [568]	1.7 million [423]	As supplement to optimize animal growth [422]	Removal of inhibitors in the cellulosic sugars stream
Microfibrillated cellulose	–	Successive refining follow by enzymatic hydrolysis and homogenization [435]	–	$232 million [569]	10,000 [440, 449]	Use in the manufacture of nanocomposites, packaging, coating, and dispersion technology [436, 439]	Large energy consumption
Polyethylene	Dehydration of fossil based ethanol to ethylene and subsequent polymerization [454]	Dehydration of bio based ethanol to ethylene and subsequent polymerization [454]	$1676 [162, 205]	$163 billion [570]	200,000 [162, 338]	Manufacture of plastic bags, plastic films, geo-membranes, bottles, and tubes [162, 205]	Relies on ethylene production Price difference between fossil and bio-based PE
Polyethylene glycol	Anionic ring opening polymerization of ethylene oxide [457], and polycondensation fossil based 1,3-PDO [458]	Polycondensation of bio-based 1,3-PDO [199, 458]	$2000 [571]	$150 million [572]	2.2 million [573]	Suitable to produce copolymers, e.g., linear, branched, star-shaped, and comb-like PEGs [457]	Relies on the success of bio 1,3-PDO Development of efficient microorganisms
Polylactic acid	Poly-condensation of fossil based lactic acid [461], and ring-opening polymerization (ROP) of dimeric lactide [462]	Poly-condensation of bio-based lactic acid [461]	$2300 [574]	$950.7 million [575]	180,000 [576]	May replace conventional synthetic polymers, especially in packaging. It is also used as insulation foam, for automotive parts, and fibres [162]	Depend on the commercial production of bio-LA Need competitive prices against fossil-based PLA
Polytrimethylene terephthalate	Transesterification using fossil based 1,3-PDO [465]	Transesterification using bio-based 1,3-PDO [465]	$2033 [189]	–	194,120 of bio-based PTT [577]	Use as thermoplastic fibers or film [199, 467]	Relies on the success of bio 1,3-PDO Requires that the industry moves towards replacing conventional polyesters
Propylene glycol	Hydration of propylene oxide, catalytic hydrogenolysis of glycerol [198]	Hydrocracking of sorbitol [142, 471, 472], hydrogenolysis of xylitol [163], and hydrogenation of LA or lactates [142]	$1530 [205]	$3.91 billion [578]	2 million [579]	Used in the production of unsaturated polyester resins, coolants and antifreeze, aircraft de-icing fluid, heat transfer fluids, paints, and coatings [142]	Due to the abundant production of glycerol, PG production will be most likely based on glycerol
Sorbitol	–	Hydrogenation of glucose [142, 471, 472], fermentation of fructose and glucose [488], and one-pot conversion [489]	$650 [162]	$107 million [162]	164,000 [162]	Used as sweetener, thickener, humectant, excipient, and dispersant in food, cosmetic, and toothpaste [142]	Separation and purification of the cellulosic sugars stream Isolation of glucose from the cellulosic sugars stream
Squalene	Extracted from shark liver oil [505]	Fermentation of glucose [506, 510]	$250,000 [503]	$ 166.1 million [580]	2400 [504]	Applications in the manufacture of fine chemicals, magnetic tape, emollient in cosmetics and pharmaceuticals, as well as an additive in animal feed [503]	Isolation of glucose and removal of inhibitors
Succinic acid	Hydrogenation of maleic anhydride or maleic acid [198]	Fermentation of sugars [514, 518]	$2500 [514]	$131.73 million [581]	50,000 [582]	Niche applications such as personal care products and food additives to large volume applications such as biopolymers, plasticizers, polyurethanes, resins, and coatings [162]	Develop of efficient and inhibitor resistant strain Isolation of glucose
Terpenes	Extracted or steam distilled [522], as a by-product of naphtha or oil cracking in the production of ethylene [524]	Microbial fermentation [524, 525]	$7000 [583]	$650 million [523]	247,100 [583]	Used to create fine perfumes, to refine the flavor and aroma of food and drinks, and to produce medicines of plant origin [522]	Isolation of glucose and removal of inhibitors
Xylitol	Hydrogenation of xylose [529]	Fermentation of xylose [532]	$3900 [162]	$624 million [162]	190,900 [584]	As a diabetic sweetener xylitol is used in the food (confectioneries and chewing gums), odontological, and pharmaceutical sectors [162]	Efficient isolation of xylose from the cellulosic sugar stream

Recent interest in the production of bio-based materials via biological process has boosted the research on microbial 1,3-PDO. While there are multiple microorganisms capable of fermenting sugars to glycerol or glycerol to 1,3-PDO, none can directly ferment sugars to 1,3-PDO [191]. Thus, an approach to generating microbial 1,3-PDO is through the use of two organisms, one that ferments sugars to glycerol and another that ferment glycerol to 1,3-PDO. Haynie and Wagner [192] patented a process for the conversion of carbohydrates to 1,3-PDO using *Saccharomyces cerevisiae* for glycerol production and a range of organisms for the conversion of glycerol to 1,3-PDO. As sugars, such as glucose, are typically less expensive than glycerol, research has been focused on the development of biological approaches to directly convert sugars to 1,3-PDO. One approach involves the co-fermentation of glycerol and sugar. Since glucose represses the 1,3-PDO pathway in native 1,3-PDO producers and glucose fermenters do not ferment glycerol to 1,3-PDO, genetically engineered organisms capable of co-fermenting glycerol and glucose are being developed. To achieve this goal, three strategies are being considered. The first strategy involves the introduction of the gene that allows the formation of glycerol from sugars or intermediates of glycolysis into a strain that produces 1,3-PDO from glycerol. For example, the expression of the glycerol-3-phosphatase that converts glycerol-3-phosphate to glycerol, into other microorganisms, would link the glucose metabolism and 1,3-PDO formation [193, 194]. In the second approach, genes that allow conversion of glycerol to 1,3-PDO are introduced into a bacterium producing glycerol from sugars. Nakamura et al. [195] developed a recombinant *S. cerevisiae* by integrating GDHt and PDOR gene of *K. pneumoniae* into the chromosome of *S. cerevisiae*. The recombinant *S. cerevisiae* fermented glucose to 1,3-PDO achieving a final concentration of 0.53 g/L. The third approach involves the introduction of both genes into an organism which could not convert glucose to glycerol nor glycerol to 1,3-PDO. For example, Chotani et al. [196] developed a strain of *E. coli* containing genes from *Saccharomyces* and *K. pneumoniae* for glycerol and 1,3-PDO production, respectively.

Cellulosic biomass price is lower than the price of sugar/starch biomass, and thus, technologies for the conversion of cellulosic hydrolyzed sugars to 1,3-PDO are being investigated. Xin et al. [197] fermented lignocellulosic hydrolysate (glucose, xylose, and arabinose) with glycerol using *Clostridium diolis* DSM 15410 strain. The results showed that co-fermentation of hydrolyzed sugars and glycerol increased the yield of 1,3-PDO by 22% compared with the fermentation of glycerol alone. Comparing the fermentation of glycerol with corn stover hydrolysate and with a mixture of sugars, mimicking the sugar profile of corn stover hydrolysate, showed that the hydrolysate by-products do not have obvious negative effects on the 1,3-PDO biosynthesis. Thus, production of 1,3-PDO using lignocellulose hydrolysate and glycerol has great potential for developing a cellulose-based biorefinery.

As mentioned, 1,3-PDO is currently manufactured by Shell and DuPont via chemical synthesis. In contrast, DuPont Tate & Lyle Bio Products have produced microbial 1,3-PDO from corn derivate sugars since 2006 at a 63,500-kilotonne per year (ktpy) 1,3-PDO plant in Loudon, Tennessee, USA [198, 199]. Four years later, DuPont Tate & Lyle Bio Products increased the plant capacity by 35% [200, 201]. More recently, in 2018, DuPont Tate & Lyle declared that due to the strong market demand for renewably sourced propanediol, the company implemented an expansion project to increase the plant capacity by another 25% which is expected to be completed in mid-2019 [201, 202]. In DuPont Tate & Lyle Bio Products’ process, 1,3-PDO is generated via fermentation of corn sugar by a genetically modified and nonpathogenic strain of *E. coli* K-12 (Fig. 2). After fermentation, *E. coli* K-12 is deactivated with a high-temperature water stream in a heat exchanger. The fermentation biomass is then separated out through a series of membrane separation steps. 1,3-PDO is separated using a three-step process involving: (i) ion exchange to remove charged impurities (ii) evaporation to remove water, and (iii) four distillation stages to remove impurities [203]. Production of bio-based 1,3-PDO requires 40% less energy than the typical petroleum-based route, giving the bio-based route a significant advantage [198]. In France, METabolic EXplorer is producing 1,3-PDO at a pilot scale by fermenting raw glycerol from vegetable or recycled oils [204]. In a 2G ethanol process, 1,3-PDO could be produced using a portion of the hydrolysate stream generated. For this approach to succeed, the impact of the by-products generated during the pretreatment and hydrolysis stages must be mitigated. Moreover, to integrate the mature 1,3-PDO technology into a cellulosic ethanol process, it would be necessary to implement separation/purification processes that generate a clean stream of glucose from the hydrolyzed stream, as shown in Fig. 2. An alternative approach would involve the development of engineered microorganisms capable of generating 1,3-PDO from a mixture of sugars.

### 1,4-Butanediol

As a bulk chemical, 1,4-butanediol (1,4-BDO) is used in the manufacture of polymers, solvents, and chemicals. 1,4-BDO is a large volume chemical that has a global market approaching two million tons per year. 1,4-BDO has a price of $2660 per tonne (2010–2014) [205], and is also used in the manufacture of automotive plastics, sneakers, soccer balls, and spandex for apparel [206, 207]. About 45% of the world’s 1,4-BDO is converted to tetrahydrofuran (THF), and approximately 25% of produced 1,4-BDO is used to manufacture polybutylene terephthalate (PBT). The next largest application of 1,4-BDO is the production of gamma-butyrolactone (GBL), which is used as a solvent and intermediate in the synthesis of herbicides [198].

The first commercial route to 1,4-BDO, the Reppe process, has two stages. In the first stage, formaldehyde and acetylene react to form 1,4-butynediol which is then hydrogenated in the second stage to produce 1,4-BDO [198]. In 1979, Mitsubishi Chemical, in Japan, developed a non-acetylenic route based on the oxidative acetoxylation of butadiene with acetic acid, followed by hydrogenation and hydrolysis to1,4-BDO [208]. In 1990, Arco Chemical developed a route to 1,4-BDO from propylene oxide (PO). This process begins with the isomerization of PO to allyl alcohol, followed by hydroformylation with synthesis gas (H_2_ + CO) to 4-hydroxybutyraldehyde. In the last step, 4-hydroxybutyraldehyde is hydrogenated to 1,4-BDO [208]. In the mid-1990s, Davy Technology, developed a route to 1,4-BDO in which maleic anhydride (MAN) is converted to its methyl or ethyl diester. The diester is then hydrogenated to 1,4-BDO. Similarly, BP Chemical developed a process in which MAN is directly hydrogenated to 1,4-BDO and THF [206, 208]. The largest producers of 1,4-BDO include BASF, Dairen Chemical Corp., Lyondell Basell, Ashland (formerly ISP), and Xinjiang Markor Chemical Industry. 1,4-BDO is mainly produced from fossil feedstocks (coal, oil, or natural gas) by processes that are energy-intensive and GHG emitters. Therefore, an environmentally friendly process for 1,4-BDO production has been under development.

The bio-based 1,4-BDO manufacture process takes place via hydrogenation of succinic acid (SA) or direct fermentation of sugars using metabolic engineered strains, as shown in Fig. 2. Hydrogenation of SA to 1,4-BDO occurs via a two-step hydrogenation reaction. SA is first transformed into γ-Butyrolactone (GBL) by hydrogenation, and then 1,4-BDO or THF is formed through hydrogenation of GBL. The catalytic performance of various noble metals such as Pd, Pt, Rh, Ru, and Re, has been investigated. To enhance 1,4-BDO production, Re-based bimetallic catalysts, such as Re–Pt/C, Re–Pd/C, Re–Pd/TiO_2_, and Re–Ru/C, have been tested [209]. Production of 1,4-BDO via fermentation is attractive due to the mild operating conditions required by the bioprocess. Nonetheless, 1,4-BDO is not a compound produced naturally by any known microorganism. Thus, microorganisms need to be modified to produce bio-based 1,4-BDO. *E. coli* has been engineered to generate bio-based 1,4-BDO at high levels from a variety of different carbohydrate feedstocks. An example of a successful metabolic engineering project is the production of 1,3-PDO in *E. coli*, developed by Genencor and DuPont, which has been commercialized. Yim et al. [210] engineered a strain of *E. coli* capable of producing 18 g/L of 1,4-BDO. The organism produced 1,4-BDO from glucose, xylose, sucrose, and biomass-derived mixed sugar streams demonstrating that a metabolic engineering approach to strain design can allow the production of valuable biochemicals via fermentation. Burgard et al. [207] engineered an *E. coli* strain that produces 1,4-BDO from dextrose, sucrose, and cellulosic biomass sugars. The engineered *E. coli* operated over 50 runs at a commercial scale to produce over 3629 tonnes of bio-1,4-BDO. Technologies to develop novel strains and processes are continuously improving, and thus, production costs are expected to decrease.

Myriant and BioAmber are working with different catalyst companies to develop processes to produce 1,4-BDO. In 2012, BioAmber (USA) scaled up its hydrogenation catalyst technology under license from DuPont and converted multi-ton quantities of bio-SA into 100% bio-based 1,4-BDO, THF, and GBL [211, 212] BioAmber planed to build a 100 ktpy 1,4-BDO plant in North America [198]. BioAmber produced bio-based SA at its plant in Pomacle, France [198], and Sarnia, Canada [213]. using glucose from agricultural sugars [214]. However, BioAmber closed its succinic plant in 2018 due to bankruptcy [215]. LCY Biotechnology (USA) was approved by courts in Canada and the USA to acquire the bio-based succinic plant [211]. Myriant (USA) partnered with Davy Process Technology Ltd. (UK) to license a process to produce 1,4-BDO using Myriant’s bio-SA [216]. Myriant officially changed its name to GC Innovation America on August 1, 2018, stating its continuing compromise to the production of biochemicals [217]. In 2008, Genomatica (USA) presented a fermentation route to 1,4-BDO using *E. coli* and commodity sugars as feedstock. By 2013, Genomatica, in partnership with DuPont Tate & Lyle, demonstrated the technical viability of producing microbial 1,4-BDO by manufacturing more than 5 million pounds of 1,4-BDO in 5-week. In 2013, BASF licensed the Genomatica technology to produce renewable polymers [198]. In 2015, Genomatica and Cargill announced a collaboration to accelerate the manufacture of bio-based 1,4-BDO [216]. While the production of 1,4-BDO via fermentation is environmentally attractive, further optimization and research are required to achieve a commercial production. Therefore, production of bio-based 1,4-BDO is most likely going to be first manufactured via the hydrogenation of bio-based SA. Nonetheless, the investment made by multiple companies, such as DuPont, Genomica, Davy Process Technology, to produce SA via fermentation [218], shows the interest of the industry to pursue biological pathways. Therefore, co-production of 1,4-BDO and 2G ethanol is technically viable, and its commercialization would depend on the optimization of microorganism and catalysts to produce first SA and consequently 1,4-BDO, respectively.

### 2,3-Butanediol

2,3-Butanediol (2,3-BDO) or 2,3-butylene glycol is an isomer of butanediol which is used to manufacture printing inks, perfumes, fumigants, moistening and softening agents, explosives, plasticizers, foods, and pharmaceuticals [219]. 2,3-BDO can be dehydrated to methyl ethyl ketone, an excellent organic solvent for resins and lacquers, and to butadiene for the manufacture of synthetic rubber. Moreover, 2,3-BDO can be dehydrogenated into acetoin and diacetyl which are flavoring agents used in dairy products, margarines, and cosmetics. Commercially, the key downstream products of 2,3-BDO have a global market of ~ 32 million tonnes per annum, valued at ~ $43 billion [220, 221]. The cost of 2,3 BDO has been reported to be $10,000 to 50,000 per ton [222]. During the World War II, the shortage of 1,3-butadiene boosted the 2,3-BDO research, culminating with the pilot-scale manufacture of 2,3-BDO and its conversion to 1,3-butadiene [219]. Nonetheless, the development of less expensive pathways to produce 1,3-butadiene from petroleum stopped the 2,3-BDO research.

2,3-BDO can be produced by catalytic hydrogenation of saccharides with hydrogen at elevated pressures and temperatures, Fig. 2. Hirth et al. [223] reported that by increasing temperature to approximately 225 °C or higher, selectivity can be shifted toward C2, C3, C4, and/or C6 polyols, such as 1,3-PDO, glycerin (1,2,3-propanetriol), 2,3-BDO, 1,4-BDO, 1,2-ethanediol, and optionally further partially hydrogenated sugar alcohols in smaller quantities. Recently, microbial production of 2,3-BDO has attracted attention as a promising technology to achieve a low-carbon economy and an environmentally friendly industry [224]. Research on the biochemical production of 2,3-BDO has focused on the fermentation of glucose [225–228], and sucrose [229]. Among the multiple microorganisms capable of accumulating 2,3-BDO, *Klebsiella pneumoniae* and *Paenibacillus polymyxa*, are among the most efficient [230]. Due to the changing and volatile prices of starch sugars [37, 231], research on microbial 2,3-BDO has explored alternative raw materials such as lignocellulose [232–235] and glycerol [236, 237]. For example, Cao et al. [238] pretreated corn cob with dilute ammonia (10%) to remove lignin and alkaline extractives. The pretreated substrate was hydrolyzed using dilute hydrochloric acid (1%, w/v) and 80 g/L of corn cob cellulose (using only cellulose) was fermented by *Klebsiella oxytoca* in a simultaneous saccharification and fermentation (SSF). A concentration of 25 g/L and 7 g/L of Butanediol and ethanol were produced, respectively, after 72 h. In 2010, Cheng et al. [239] used *Klebsiella oxytoca* ACCC 10370 to produce 2,3-BDO from acid hydrolyzed and detoxified corn cob. Cheng’s work is relevant because the hydrolysate composition used (xylose, glucose, arabinose, cellobiose, galactose, mannose, and acetate) is similar to that of the lignocellulose hydrolysate. Results showed that fermentation was inhibited by the high concentration of acetate. Glucose, cellobiose, mannose, and galactose were not detected at the end of fermentation. In contrast, xylose achieved 97% consumption and arabinose was partially consumed (30%) [239]. A maximal 2,3-BDO’s concentration of 35.7 g/l was obtained after 60 h of fed-batch fermentation. These results indicate that a fraction of the hydrolysate produced during the 2G bioethanol process may be suitable to generate 2,3-BDO. Nonetheless, it is important to consider the impact of by-products on microorganisms. Lee et al. [240] showed that phenolic compounds are highly toxic, inhibiting cell growth and reducing 2,3-BDO production and enzyme activity. Similarly, furan derivatives and weak acids were shown to have a detrimental impact on fermentation. In contrast, no significant effects were reported for acetic acid and formic acid. In line with this, Joo et al. [241] reported the negative influence of furans and phenolic compounds on 2,3-BDO production and cell growth. Thus, strategies to enhance 2,3-BDO production, such as genetic engineering of 2,3-BDO producers [233, 242, 243] and development of separation process, are needed [229, 244–246].

Global Biochem is currently producing 2,3-BDO through the hydrogenation of corn-based sugar in the province of Jilin (Xinglongshan, Dehui, and Changchun), China [179, 247]. Other companies involved in the manufacture of 2,3-BDO from corn are Novepha and Zhangjiagang Glory Biomaterial, also in China [162, 248]. Due to the abundance of shale gas, ethylene manufacturing has shifted feedstock from naphtha to shale gas. Since butadiene is a by-product of the ethylene manufacturing, the supply of butadiene has been restricted and large price fluctuations have occurred over the past years [198]. As a result, the demand for bio-derived 2,3-BDO for the production of butadiene has increased. Despite the advantages of microbial 2,3-BDO, more research and industrial efforts are needed to scale-up this technology. Production of 2,3-BDO via hydrogenation of sugars is an immediate available technology that can improve the economic viability of cellulosic bioethanol. Even though more information on process design, operating conditions, and market prices is necessary to determine the viability of co-producing 2,3-BDO and cellulosic ethanol, the catalytic hydrogenation of saccharides, used by Global Biochem, Zibo Shuangyu Chemical, and Cargill, among others, is a mature technology capable of adding value to a cellulosic biorefinery.

### Acetaldehyde

Acetaldehyde is used in the manufacture of acetic acid, perfumes, dyes, and drugs, as a flavoring agent and as an intermediate in the metabolism of alcohol. It is an important raw material in the production of paint binders in alkyd paints and plasticizers for plastics, and in the manufacture of construction materials, fire-retardant paints, explosives, and acetic acid [249]. Acetaldehyde market was valued at USD 1.26 billion in 2016 and is projected to reach USD 1.80 billion by 2022 [250]. China is the world’s largest consumer of acetaldehyde. In 2016, the country accounted for almost half (45%) of global consumption for acetaldehyde. India is the second largest consumer accounting for about 14% of world consumption [250].

**Fig. 3:**
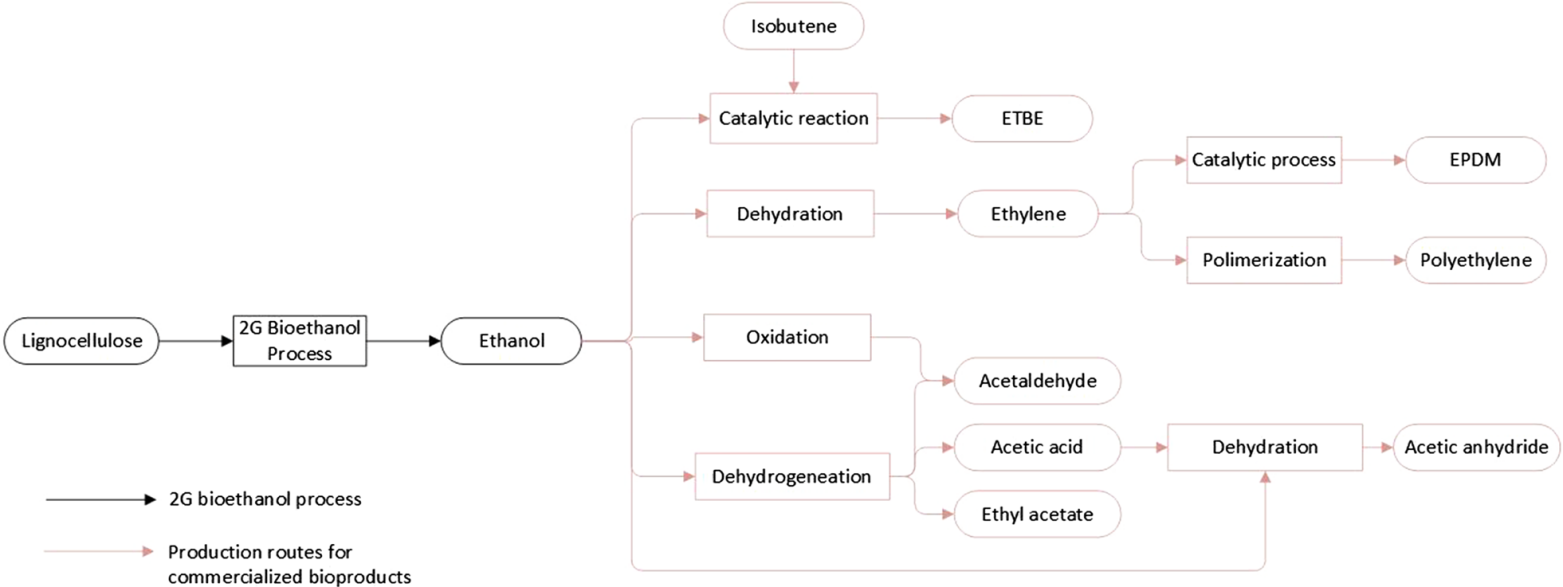
Process diagram for the production of second-generation bioethanol from lignocellulose. Production routes to produce biochemicals from ethanol that are currently produced industrially (red lines)

Manufacture of acetaldehyde is carried out via the dehydrogenation or oxidation of ethanol (Fig. 3), addition of water to acetylene, partial oxidation of hydrocarbons, and direct oxidation of ethylene [251]. Fossil-based ethanol has been the preferred raw material for the production of acetaldehyde, whereas bio-based ethanol is used on a small scale [252]. In the dehydrogenation process, ethanol vapor (260–290 °C) is passed over a catalyst consisting of copper sponge or copper activated with chromium oxide in a tubular reactor, achieving a conversion of 25–50% per run [253]. Acetaldehyde is then washed out from the exhaust gas with alcohol and water. Pure acetaldehyde is distillated while ethanol is separated from water and higher-boiling-point products to be fed back to the reactor. The final acetaldehyde yield is about 90%, and the by-products obtained include butyric acid, crotonaldehyde, and ethyl acetate [252]. In the oxidation process, ethanol is oxidized catalytically with oxygen, or air in the vapor phase in the presence of a catalyst, such as copper, silver, and their oxides or alloys [253, 254]. Information about alternative processes for the production of acetaldehyde can be found in the review written by Eckert et al. [252].

Biomass-based acetaldehyde is currently produced by Sekab, a Nordic producer of ethanol and ethanol derivatives, such as acetic acid and ethyl acetate. Sekab manufactures acetaldehyde from 1G and/or 2G bioethanol via the catalytic oxidation of ethanol. The process takes place using renewable bioenergy in closed loop systems [255]. Sekab reports that the difference in CO_2_ emissions between fossil- and biomass-derived acetaldehyde is significant, 5.7 kg and 0.75 kg CO_2_ per kg of produced acetaldehyde, respectively [249]. Sekab operates a chemical plant capable of producing 42 ktpy of acetaldehyde in Sweden [256, 257]. Sekab’s operations point to the maturity of the ethanol to acetaldehyde technology. Thus, production of acetaldehyde can be implemented in the cellulosic ethanol process as an integrated stage or stand-alone plant operated by a third party. Co-production of acetaldehyde and ethanol could add flexibility to a cellulosic biorefinery, allowing the adjustment on production rates for each product, depending on the ethanol and acetaldehyde selling prices.

### Acetic acid

Acetic acid (AcOH) is a valuable compound used as raw material in the production of multiple polymers. More than 65% of the acetic acid produced worldwide is converted to vinyl acetate or cellulose-based polymers, e.g., vinyl acetate monomer (VAM), poly(vinyl acetate PVAc), ethylene and vinyl acetate (EVAc) or poly(vinyl butyral PVB). Some of these applications include foam rubber, cable insulation, wood gluing, emulsifiers, cement coatings, and desalination membranes [142]. AcOH has an installed world capacity of 8.3 mtpy [161], a price of $617/tonne, and total sales of MM$8373 per year from which 10% comes from bio-based AcOH [162]. Industrially, acetic acid is produced from methanol carbonylation using the Rh-catalyzed Monsanto or Ir-catalyzed Cativa process [258]. The Monsanto process operates under mild conditions (150–200 °C, 30–60 bar), using iodide rhodium catalyst. Monsanto delivers high selectivity based on methanol (ca. 99%) and carbon monoxide (> 85%) [259]. In contrast, the Ir-catalyzed Cativa process uses iodo carbonyl ruthenium complexes or their osmium analogues to promote the iridium-catalyzed carbonylation of methanol to acetic acid [260]. Additionally, AcOH may be produced in the liquid phase from aqueous ethanol using supported gold catalysts (Fig. 3). Christensen et al. [261] used a batch reactor at 180 °C and supported gold catalysts to achieve a selectivity of 86% for AcOH. Similarly, Volodymyr et al. [262] obtained AcOH from aqueous ethanol solutions over Cu/ZnO–ZrO_2_–Al_2_O_3_ catalyst at 250–320 °C and atmospheric pressure. A selectivity of 80–90% and an ethanol conversion of 60–80% were obtained while processing 14–37 mol % aqueous ethanol solutions. In the gas phase, AcOH has been produced using a multi-component catalyst Mo_0.61_V_0.31_Nb_0.08_O_x_ mixed with TiO_2_ colloids. The multi-component catalyst achieved 95% selectivity to AcOH at 100% ethanol conversion [263]. Catalytic production of AcOH can be carried out using either fossil- or biomass-based ethanol. Biological routes to produce AcOH, such as the oxidative fermentation of ethanol using *Acetobacter* or the direct fermentation of sugar to AcOH, are still under development [264].

Sekab currently produces 24 ktpy of AcOH from bio-based ethanol at a chemical plant in Örnsköldsvik, Sweden [256]. As mentioned earlier, Sekab produces 1G and 2G ethanol that are used as raw material to produce acetaldehyde, EA, and AcOH [257]. Jubilant Life Science manufactures bioethanol from sugarcane molasses at its facilities located in India (Uttar Pradesh and Maharashtra). Jubilant Life Science has an annual capacity of 52,000 tonnes of ethanol that is sold and used in the production of value-added end-products such as AcOH [265]. In 2004, Songyuan Ji’an Biochemical Co., Ltd., built a plant capable of producing 150 ktpy of AcOH from bioethanol [266]. Since then, the company was restructured as Songyuan Laihe Chemicals Co., Ltd., and is now focused on the production of microbial butanol [267]. Using a patented biocatalytic process, ZeaChem generates cellulosic sugars that are converted to 2 and 3 carbon organic acids and acetates, such as AcOH and propionic acid, at its demonstration scale facility in Boardman, Oregon, USA [266]. Similar technologies could be introduced into a cellulosic ethanol process to convert a portion of the hydrolysate to AcOH. Nonetheless, the technological and economic viability of these technologies needs to be proved. A more immediate alternative, a fraction of the ethanol produced in a cellulosic ethanol process could be converted to AcOH through a chemical catalytic reaction, adding flexibility to produce different product rates and adapt to market changes.

### Acetic anhydride

Acetic anhydride (Ac_2_O) is an esterification agent used in the preparation of modified food starch and acetylation of monoglycerides. In the agrochemical industry, AcOH is used as a solvent and as a key ingredient to manufacture pesticides and herbicides [268]. The global acetic anhydride market reached a volume of 2.7 million tons in 2018 and is further expected to reach a volume of around 3.4 million tons by 2024 [269].

The AcOH dehydration process was one of the first processes used to generate Ac_2_O. This process involves the pyrolysis of AcOH to ketene and water with subsequent trapping of the ketene gas with added AcOH to generate Ac_2_O (Fig. 3) [270]. Alternatively, Ac_2_O can be manufactured via acetone cracking, in which acetone is cracked to ketene which later reacts with AcOH to form Ac_2_O [271]. Another option is via acetaldehyde oxidation, in which oxygen or air is employed to oxidize acetaldehyde in the presence of cobalt acetate catalyst promoted by copper acetate. The peracetic acid obtained from this reaction reacts with AcOH to generate Ac_2_O [272]. Methyl acetate carbonylation is the most successful coal-based chemical process. In this process, AcOH and methanol generate methyl acetate. Through the methyl acetate carbonylation reaction, methyl acetate is converted to Ac_2_O. The possible catalysts used in the methyl acetate carbonylation are rhodium chloride trihydrate, methyl iodide, chromium metal powder and alumina support or nickel carbonyl complex with tri-phenyl phosphine, methyl iodide and chromium hexa-carbonyl [273].

Industrially, only Jubilant Life Science, the seventh largest global manufacturer of Ac_2_O, produces bio-based acetic anhydride. At Jubilant Life Science’s facilities in Gajraula and Nira, India, 1G ethanol is used to generate AcOH which is then transformed to Ac_2_O [274]. In 2007, Jubilant reported that the Gajraula’s plant operated at 80,000 tonnes/year acetic acid plant and 33,000 tonnes/year acetic anhydride [275]. More recently, in 2018, it was reported that Jubilant was planning to bring onstream a 50,000 tonne/year acetic anhydride project in 2019. This would translate to an additional production of 65,000 tonnes/year of acetic acid. To produce Ac_2_O in a cellulosic ethanol biorefinery, it would be necessary to first convert 2G ethanol to AcOH. As either fossil- or biomass-based ethanol can be used to generate AcOH, the economic viability of producing bio-based Ac_2_O would depend on the capacity of the oil and gas industry to cover the growing demand of Ac_2_O and other ethanol derivatives, as well as on the production cost of bio-based ethanol.

### Acetone–butanol–ethanol

Acetone is a largely manufactured chemical with total market sales of MM$7700/year, in 2015, from which 3.2% of the produced acetone is obtained from biomass and $5.75 billion in 2016 [162]. Acetone, $1210/tonne (2010–2014) [205], is an intermediate feedstock in the production of acrylic plastics, signs, lighting fixtures and displays, and bisphenol A (BPA), which is a raw material in the manufacture of polycarbonate and epoxy resins. Acetone is also used as an intermediate in pharmaceuticals and as a solvent in multiple products, such as paints, cleaning fluids, nail polish remover, and adhesives [276]. The first processes used to produce acetone were based on the thermal decomposition of calcium acetate or the fermentation of corn starch or molasses. The large availability of propylene in the 1960s led to the development of acetone routes based on the dehydrogenation of isopropyl alcohol or cumene oxidation [277]. Today, nearly 90% of the acetone is produced via cumene oxidation. In this process, benzene reacts with propylene in the presence of phosphoric acid-based catalysts or zeolite catalysts to generate cumene [264]. Cumene is then oxidized to cumene hydroperoxide, which is then cleaved in the presence of sulfuric acid to phenol and acetone. The decarboxylative ketonization of AcOH, catalyzed by several dispersed metal oxides (e.g., CeO_2_, MgO, MnO_2_, CdO, and La_2_O_3_), is an alternative for the production of acetone [278, 279].

Butanol has a total market sales of MM$3750 to MM$4650 per year, in which 20% of the market is made up of bio-based butanol [162]. Butanol price has been reported to be around $1463 per tonne (2010–2014) [205]. Butanol is used in the manufacture of adhesives, sealant chemical, paint additives, coating additives, plasticizer, and cleaning products [280]. The main commercial source of 1-butanol is n-butyraldehyde, which is produced from the Oxo reaction of propylene. The mixture of n- and iso-butyraldehyde obtained from the Oxo reaction is either separated, or the mixture of isomeric aldehydes is hydrogenated directly and the n- and iso-butanol product separated by distillation [280].

Alternatively, acetone, butanol, and ethanol can be co-produced via the fermentation of sugars in a process known as acetone–butanol–ethanol (ABE) process [264, 281]. The ABE fermentation is carried out in two stages: In the growth stage, acetic and butyric acids are produced, while the second stage is characterized by acid re-assimilation into ABE solvents. Carbon dioxide and hydrogen are by-products of the ABE fermentation [282]. One of the most important strains used in the ABE production is the *Clostridium* genus, for example, *C. acetobutylicum, C. beijerinckii, C. saccharoacetobutylicum, C. aurantibutyricum*, and *C. sporogenes* [283]. *C. beijerinckii* and *C. acetobutylicum* are reported as the most efficient and promising species for commercial and bench applications. These microorganisms can ferment un-hydrolyzed starch and a wide range of simple sugars [284–286]. The main barrier to feasible ABE fermentation is the fact that more than 5.4 tonnes of corn is required to produce one ton of butanol [287]. In a conventional plant, corn starch accounts for up to 79% of the overall solvent production cost, while energy requirements contribute to 14% of the overall cost [288]. Therefore, challenges for ABE fermentation include the need for cheaper feedstocks, improvement in microorganisms’ performance, development of more sustainable solvent recovery, and water recycle processes. Extensive research on the use of low-cost lignocellulose as feedstock in the ABE process is being carried out. An important barrier to the efficient ABE fermentation of lignocellulose derivate sugars is the inhibitory effect of compounds present in the lignocellulose hydrolysate, e.g., hydroxymethylfurfural, furfural, and lignin derivatives [287, 289]. Moreover, butanol is highly toxic to the fermenting microorganisms [290]. Thus, a variety of fermentative microbes and strategies to improve strain tolerance to inhibition are being investigated [291]. For example, an iterative process of genetic diversification followed by selection was applied to find a mutant yeast from *Pichia stipitis* with increased tolerance to pentose [292]. Considering that the lignocellulosic-derived hydrolysate can be rich in hemicellulose derived sugars, it is important to develop pentose sugar-resistant organisms. This approach was demonstrated in China by Songyuan Laihe Chemicals, which operates a 600-tpy pilot plant capable of fermenting sugars contained within the hemicellulose fraction of corn stover [282]. Cathay Industrial Biotech, with facilities in Shandong and Jilin, China, is currently developing cellulosic inhibitor-resistant strains capable of fermenting both hexo- and pento-sugars [293]. Another challenge for the ABE fermentation is the efficient and economic recovery of products. Since concentration of ethanol at a commercial scale is typically 5–9% [294], and the final concentration of products after ABE fermentation is 2–4% [294], costs of separation and purification have an important impact on the process’ feasibility. Solvent recovery using conventional distillation is robust and proven, but energy intensive. Thus, non-conventional methods are required to reduce energy requirements and associated costs. The integration of the solvent recovery and fermentation stage is an attractive strategy to reduce costs but also to relieve butanol toxicity. Therefore, the gas stripping is a technique that can be applied for in situ butanol recovery during the ABE fermentation, alleviating the end-product inhibition and improving both solvent titer and productivity [294]. Other methods for solvent recovery include liquid–liquid extraction, adsorption, pervaporation, reverse osmosis, and aqueous two phase separation [282, 287].

**Fig. 4:**
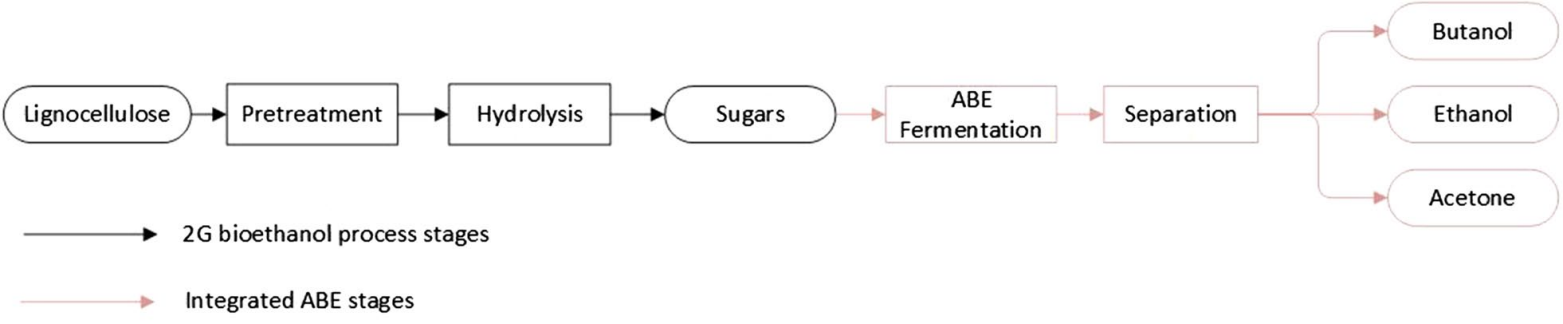
Process diagram for the production of acetone–butanol–ethanol (ABE) from lignocellulosic biomass. 2G ethanol process stages (black lines) and integrated ABE stages (red lines)

ABE production using *Solventogenic clostridia* was one of the first large-scale industrial microbial processes for chemical production. In 1950–1960s, the ABE process ceased completely in Europe and North America due to the cheaper petrochemical synthesis method. In China, ABE process was in operation until the end of the last century when butanol prices dropped [295]. Nonetheless, due to unstable oil prices and increasing environmental problems, production of biobutanol is resurging. China leads efforts to re-commercialize the ABE fermentation process. In 2008, the annual production of solvents in China was around 210,000 tonnes. In the last decade, multiple companies in Asia, such as Cathay Industrial Biotech, Jiangsu Lianhai Biological Technology, Laihe Rockley Bio-Chemicals, Lianyungang Lianhua Chemicals, Shi Jinyan, Songyuan Ji’an Biochemical, Tongliao ZhongKe, and Tianyuan Starch Chemical, have produced bio-based acetone and butanol [162]. In 2014, Cathay Industrial Biotech, a major player in the biofuel and biochemical industry, announced a project to build a 200-ktpy microbial butanol plant with acetone, ethanol, corn starch, and particle feed as by-products [296]. However, in 2015, Cathay Industrial Biotech abandoned its initial public offering (IPO) plans and idled its bio-butanol production in China [297]. Unfortunately, with the recent drop in oil prices and the relatively high corn starch prices, multiple biobutanol plants that started operations in 2008 have stopped production [267]. In contrast, Lignicell Refining Biotechnologies continued operating a 40 ktpy biobutanol plant in Songyuan, China, by switching its feedstock from corn starch to lignocellulose (mixed corn stover and corn cob), as shown in Fig. 4. This is the only commercialized lignocellulosic bio-based butanol and acetone plant in the world [267]. In 2014, Green Biologics acquired the assets of Central MN Ethanol Co-op LLC in Little Falls, MN, USA, including a 65-ktpy ethanol plant. The plant was adapted to produce approximately 30 ktpy of acetone and butanol using corn starch and has the flexibility to utilize wood-based lignocellulosic feedstocks [298]. While the production of butanol from lignocellulose has reached industrial scale, its profitability, in the long run, remains to be proven. Nonetheless, ABE fermentation remains one of the most promising biological processes, especially in a biorefinery frame. A key characteristic of the ABE fermentation is that acetone, butanol, and ethanol are produced at a ratio of 1:6:3, respectively, using *C. acetobutylicum* [287]. Therefore, the integration of the ABE fermentation into a cellulosic ethanol process switches the idea that 2G bioethanol would be the foundation of a lignocellulose-based biorefinery. As a result, an extensive market analysis would be required to define which biochemical would be the most profitable.

### Ethyl acetate

The chemical ethyl acetate (EA) is an organic compound used in the production of inks, adhesives, car care chemicals, plastic, and as synthetic fruit essence, flavor and perfume in the food industry [299]. The world production of EA increased by more than 80% between 2004 and 2011 and went beyond 3.2 million tonnes in 2013. The global market for EA is growing at about 4–5% per year [300], with a price of $1434–1507 per tonne in the US [301]. EA can be produced via esterification of ethanol with acetic acid (Fisher esterification), directly from ethanol by dehydrogenation, via the dimerization of acetaldehyde in the presence of aluminum, sodium alkoxide, or solid bases like alkaline earth oxides (Tishchenko reaction) [302]. The dehydrogenation of ethanol is attractive because large amounts of ethanol are expected to be available as feedstock in the near future. In this reaction, EA is synthesized from liquid ethanol using a Cu/Cr_2_O_3_ catalyst at 220 °C and 15 atm. The selectivity to EA is > 95% with H_2_ as the only by-product [303]. Published studies have evaluated the use of alternative catalysts: copper/copper chromite, and copper metal supported and/or promoted by different oxides such as Al_2_O_3_, Cr_2_O_3_, ZnO, ZrO_2_, and SiO_2_ [303–307]. Depending on the adopted catalyst and operating conditions, acetaldehyde or EA is obtained as main products. Other by-products, mainly derived from acetaldehyde, may be generated [306].

Industrially, EA is mainly produced from either oil- or fossil-based ethanol. Nonetheless, in India, Dhampur Sugar Mills Limited produces refined sugar, bioethanol, biochemicals, and energy from sugarcane at a commercial scale [308]. Dhampur Sugar Mills Limited owns and operates five integrated sugarcane complexes located in India (Dhampur, Asmoli, Mansunpur, Rajpura, and Meerganj) which generate 300,000 litres of 1G ethanol and 140 tonnes of EA per day [309]. In Örnsköldsvik, Sweden, Sekab operates a chemical plant capable of producing 42,000 tonnes of acetaldehyde, 36,000 tonnes of EA, and 24,000 tonnes of AcOH annually [256]. SEKAB manufactures EA using 1G and/or 2G ethanol as raw material [257]. Recently, Helm AG signed an offtake agreement for the sale and marketing of bio-based EA produced at Greenyug’s planned facility in Columbus, Nebraska, which will be owned and operated by Greenyug’s subsidiary, Prairie Catalytic LLC [310]. Furthermore, Jubilant Life Science, Laxmi Organic, and Zeachem are producing bio-based EA [161, 162, 311]. A large number of EA manufacturers and industrial players interested in the production of EA from bioethanol, indicate that the technology to manufacture EA from ethanol is mature. Since bio-based EA is a chemical equivalent of the fossil-based EA, its access to the market will be quick and with low risks. However, for the bio-based EA industry to grow, bio-EA’s production cost must be competitive with that of fossil-based EA. At the current low oil prices, and relatively high 1G bioethanol price, bio-based EA is attractive due to its environmental advantages. Consequently, to expand the use of bio-based EA, its production cost needs to be reduced. Considering lignocellulose’s low price, production of EA from cellulosic ethanol may offer economic advantages, especially if the production cost of cellulosic ethanol is considerably reduced. Technically, the process to generate EA could be implemented at the end of the 2G ethanol process, right after the separation stage. At this point, fuel grade bioethanol is available to be dehydrogenated to EA, as shown in Fig. 3. Thus, EA could be integrated into a cellulosic ethanol process, or operated as a stand-alone process by a third party. Co-production of EA and cellulosic ethanol could add flexibility to the process by allowing adjustments on the production rates depending on market changes.

### Ethyl lactate

Ethyl lactate (EL) is a lactic acid (LA) derivative with superior properties to many conventional petroleum-based solvents such as toluene, methyl ethyl ketone, and N-methyl-pyrrolidone [198]. It can be blended with methyl soyate derived from soybean oil to create custom-tailored solvents for various applications. Selling prices for EL range between $3.30 and $4.40/kg [312]. In contrast, conventional solvents prices range from $2.00 to $3.75/kg [313]. Since LA is a precursor of EL, advances in LA fermentation, separation, and conversion have driven down EL’s retail costs as low as $1.87/kg [312]. EL has an installed world capacity of 1.2 million tonnes per year [161]. Experts have suggested that EL can replace conventional solvents in more than 80% of their applications. However, since the boiling point of EL is 151–155 °C, much higher than for most fossil-based solvents, products (e.g., paint, glue, etc.) containing EL might need to be redesigned. From the industry perspective, this substitution is often seen as complicated as the development of a new product [161].

The conventional production of EL involves the esterification of LA with ethanol catalyzed by an acid catalyst [312]. Thus, EL can be produced using fossil-based LA, and synthetic ethanol from ethylene or acetic acid. Similarly, lactic acid and ethanol derived from biomass can be used to generate a bio-based EL, as shown in Fig. 3. It was reported that all EL is currently produced from ethanol and LA made from renewable sources (e.g., starch or sugar) [198]. Typically, EL is synthetized through the esterification of ethanol and LA until equilibrium followed by EL removal by distillation. To overcome the equilibrium limitation, excess ethanol is added to shift the equilibrium toward EL conversion [312]. Alternatively, EL can be synthetized from ammonium lactate by coupling solvent extraction with esterification. Dimethyl sulfoxide, *N*-methyl pyrrolidine, and triethyl phosphate are some of the solvents evaluated for the extraction esterification process [314]. Arkema (France) patented two processes for the continuous production of EL: The first consists in extracting a mixture of EL, ethanol, water, and different heavy products from the reaction medium at partial LA conversion rate. Subsequently, the mixture is fed to a reduced-pressure flash separation, producing an overhead stream that is processed in a fractional distillation column. An EL purity higher than 95% was reported for this process [315]. The second patented process uses a continuous extraction of a near-azeotropic water/ethanol gas mixture, which is then dehydrated using molecular sieves. An EL purity higher than 97% was claimed using this arrangement [316]. A process to produce EL directly from cellulose using the mesoporous Zr-SBA-15 silicate catalyst in a supercritical mixture of ethanol and water has been also reported. The process achieved a yield of around 33% of EL at optimal conditions: 260 °C in supercritical 95:5 (w/w) ethanol/water solution [317].

Galactic (Belgium), which produces 45 ktpy of LA from sugar beet, also manufactures EL, under the brand name of Galaster™ EL, via esterification of natural LA with ethanol [162]. Galactic operates a facility in Milwaukee, Wisconsin, USA, with a capacity of 15 ktpy capacity of EL [198]. Another company producing EL is ADM (USA), which has an annual production of 1800 MMgy of ethanol [312, 318]. NatureWorks (Cargill-Dow) operates a LA and “green” solvents, such as EL, facility in Blair, Nebraska, USA [198]. Another company, Vertec BioSolvents (USA) is a formulator and reseller that provides EL under the brand name of VertecBio™ EL. Applications targeted by Vertec Biosolvents include conventional solvents that are under environmental scrutiny such as methylene chloride, methyl ethyl ketone, and *N*-methyl pyrrolidone [161]. The technology to produce EL from LA and ethanol is considered mature with room for improvement, especially in the separation stage. Nonetheless, the economic viability of producing EL would be defined by its demand and the cost of LA and ethanol. The use of lignocellulose-derived LA and ethanol to produce EL is technically possible. However, 2G ethanol production is not currently viable, and while production of LA is technically advanced, further economic analyses are needed to define its economic viability. Therefore, the growing LA market and the successful replacement of fossil solvents with EL will define the feasibility of manufacturing bio based EL.

### Ethyl *tert*-butyl ether

In the 1970s, the severe environmental air pollution related to the automotive emissions resulted in the regulation of the automobile exhaust compounds. Reduction on exhaust compounds can be achieved by the reformulation of fuels using additives. Before the 1970s, tetraethyl lead was used as an antiknock agent to increase the octane rating. However, the use of tetraethyl lead was terminated with the Clean Air Act of 1970 [319]. Methyl *tert*-butyl ether (MTBE) was then introduced into gasoline [320]. In 1996, a US Geological Survey study reported that MTBE was frequently found in the urban groundwater supplies sampled. Due to MTBE’s negative environmental impact, ethyl tert-butyl ether (ETBE) was then introduced as additive [321]. As a result, ETBE is used as an additive in several European countries, including France, Netherlands, Germany, Spain, and Belgium [322]. In 2010, Japan started blending 7% bio-ETBE into automobile fuel in accordance with the Kyoto Protocol Achievement Plan [323]. ETBE production capacity increased from 1.8 million tonnes to 3.6 million tonnes from 2005 to 2007 [322].

In the industry, ETBE is produced using liquid phase isobutene and ethanol as the reactants and is usually catalyzed by macroporous sulfonic acid resins at a temperature below 80 °C under pressurized conditions of 6 atm. A series of separation processes are applied to obtain a higher-purity ETBE, while excess ethanol is recycled [324, 325]. Ethanol can come from a renewable source, such as wheat, beet, or lignocellulose, while isobutene is derived from crude oil or natural gas (Fig. 3). The isobutene sources include cracked stocks from refineries and steam crackers, or from chemical plants via dehydrogenation or dehydration processes [326]. The Brazilian company Braskem produces ETBE partially from ethanol, at an industrial unit in the Triunfo Complex in Rio Grande do Sul since 2007, and at two units in the Camaçari Complex in Bahia since 2009. Production of 1 T of ETBE using bioethanol (43%) and isobutene (57%) prevents 783 kg of CO_2_ emissions [327]. While an active research on alternative technologies to produce ETBE is currently being conducted, e.g., gas-phase reaction, use of *tert*-butyl alcohol (TBA) instead of isobutene, hybrid process, or pervaporation membrane hybrid process [319], the Braskem’s process is considered to be mature and readily applicable to a 2G ethanol process as shown in Fig. 3. Therefore, cellulosic ethanol can be used to generate bio-ETBE. Economic analyses, considering future market demand, are required to define the viability of producing ETBE in a cellulosic ethanol plant.

### Ethylene

The importance of ethylene comes from the wide range of high-volume plastics derived from it, e.g., polyethylenes (high-density polyethylene (HDPE), low-density polyethylene (LDPE), and linear low-density polyethylene (LLDPE)), polyvinylchloride (PVC), and polyethylene terephthalate (PET) [161]. Ethylene, $1370 per tonne (2010–2014), has a total market sale of MM$140,000–203,000 per year, including bio-based ethylene (0.2% of total market) [162, 205]. The largest consumer of ethylene is polyethylene, e.g., HDPE and LDPE, while other major consumers are mono ethylene glycol (MEG) and polyvinyl chloride (PVC). In the early twentieth century, ethylene was produced from ethanol, but, due to the unbeatable price of oil, production shifted to the petrochemical route. Fossil-based ethylene is produced via the steam cracking of hydrocarbons (ethane, propane, naphtha, or gas oil) at 750–950 °C [142]. About 99% of the global ethylene is synthesized using this method [328]. In this reaction, the individual product yield depends on the feedstock used, for example, cracking ethane produces almost no co-products, but cracking naphtha results in substantial amounts of propylene, butadiene, and benzene. While the steam cracking is a mature technology, optimization opportunities are under development, e.g., in 2014, ExxonMobil announced that it was directly cracking crude oil in its Singapore-based steam cracker facility [329].

Alternatively, ethylene can be produced via dehydration of ethanol. A promising route to generate ethylene from biomass is by the combination of the Bio-Synfining™ process with steam cracking [330]. Bio-Synfining™, a technology developed by Syntroleum Corporation (Tulsa, Oklahoma, USA), which catalytically converts triglycerides and/or fatty acids from fats, algae, and vegetable oils to synthetic paraffinic kerosene or diesel and renewable naphtha. In 2014, Renewable Energy Group (REG) launched the Synthetic Fuels Division which applies the Bio-Synfining™ technology in a 75 Million gallons per year (MMgy) nameplate capacity biorefinery [329, 330]. In contrast, ethylene production via vapor-phase dehydration of ethanol generates ethylene with > 99% conversion and > 99% selectivity. Currently, alumina-based catalysts are used in most of the industrial ethylene reactors, which operates at 300–500 °C, 0.1–0.2 MPa, and a space velocity of 0.1–1 h^−1^ [328]. Since the production of 1 tonne of ethylene requires 1.7 tonnes of ethanol, a single ethylene plant would be a considerable consumer of ethanol [161].

Braskem is the first commercial producer of ethylene from bioethanol. Braskem uses low cost sugarcane ethanol produced in Brazil to manufacture ethylene. In 2010, Braskem commissioned a 200 ktpy plant to produce ethylene [331], which is distributed in the Asia-Oceania region by Toyota Tsusho, while Tetra Pak, Nestlé, and Johnson & Johnson use Braskem’s ethylene in their products and packaging [162]. Solvay Indupa uses sugarcane to generate ethylene and bio-based PVC as final product. Solvay Indupa operates a 220 ktpy of PVC facility in Bahia Blanca, Argentina, and a 290 ktpy plant in Santo André, São Paulo, Brazil [332]. The Dow Chemical Company and Mitsui operate a 350-ktpy ethanol to ethylene plant in Brazil. Recently, Mitsui sold its entire shares of the Santa Vitória Açúcar e Álcool Ltda, a green chemicals venture, to the Dow Chemical Company.

Taylor et al. [162] reported that in India and Brazil, where ethanol feedstocks are relatively inexpensive and easily accessible, bio-ethylene production costs are close to those for fossil-based ethylene ($900–1100/tonne). Bio-ethylene’s prices in Brazil and India (sugarcane) are typically $1200/tonne, while in China (sweet sorghum feedstocks) they are around $1700/tonne. In the USA and Europe (mainly corn), bio-ethylene costs were reported at about $2000/tonne and $2600/tonne, respectively. Moreover, ethylene produced from sugarcane is estimated to save about 60% of fossil energy and reduce by 40% the GHG emissions compared to the petrochemical production. Bio-ethylene from corn and lignocellulose save less energy and GHG emissions because the related processes produce less electricity. If all bioethanol currently produced for the transport sector (~ 61 million tonnes) were converted to bio-ethylene, it would meet about 25% of current global demand. Nonetheless, industrial sectors, such as the transportation fuels, the power generation, and the chemical industry, might compete for the availability of biomass feedstock [333]. Thus, the development of cheap and sustainable processes for the conversion of lignocellulose is crucial to increasing the resources of sustainable biomass. Mohsenzadeh et al. [334] performed an economic analysis on the conversion of starch derivate bioethanol to ethylene, concluding that the impurities in the ethanol feed do not affect the quality of produced ethylene, significantly. Thus, extensive purification steps are not required. However, the economic evaluation showed that the process is not profitable at the current prices for raw materials and products. Since the cellulosic bioethanol production is currently in the demonstration stage, the idea of immediately co-producing ethylene and ethanol from lignocellulose, as shown in Fig. 3, appears unattractive. However, in Brazil and India, which possess a mature sugar industry, production of ethylene and 2G bioethanol from sugarcane bagasse may be more feasible and applicable.

### Ethylene glycol

Ethylene glycol (EG) price has been reported to be around $1144 per tonne (2010–2014) [205], and it is used in the manufacturing of antifreeze in cooling and heating systems, hydraulic brake fluids, industrial humectants, printer’s inks, stamp pad inks, and inks for ballpoint pens, as well as in the synthesis of safety explosives, plasticizers, synthetic fibers, and synthetic waxes [335]. Moreover, mono ethylene glycol (MEG), a form of EG, can be co-polymerized with terephthalic acid to produce poly(ethylene)terephthalate (PET), which is commonly used for the production of plastic bottles and textile fibers. As Coca-Cola Company and Pepsi introduced PET bottles containing 100% renewable MEG, the demand for EG has increased, creating a specific market for bio-based EG [161]. EG is manufactured via the hydrolysis of ethylene oxide (EO). EO is produced by the oxidation of ethylene in the presence of oxygen or air and a silver oxide catalyst. The crude EG mixture produced from the hydrolysis of EO is fed to evaporators to remove water. Fractional distillation under vacuum is used to separate MEG from diethylene glycol (DEG) and triethylene glycol (TEG) [142, 336].

Alternatively, sorbitol can be hydrogenated, in the presence of a catalyst (e.g., ZrO_2_-supported bimetallic Pd–Cu), to polyol products, such as ethylene glycol, as shown in Fig. 2 [337]. Currently, Global Biochem produces 2.2 ktpy of EG, as well as propylene and other polyols from corn starch-based sorbitol [178]. In India, India Glycols Limited has set up an EG plant in technical collaboration with Scientific Design Inc, US [161, 338]. In 2013, Greencol Taiwan Corporation (GTC) completed a facility capable of producing 100 ktpy of bio-MEG in Kaohsiung, Taiwan. Produced bio-MEG is supplied to PET manufacturers in Asia. GTC’s plant converts sugarcane bioethanol, supplied by Petrobras Biocombustível (143,000 m^3^/year), to ethylene using Petron Scientech’s technology [336]. In China, Novepha Company Limited manufactures EG from corn bioethanol [336]. Thus, to convert sorbitol to EG in a cellulosic biorefinery, the hydrogenation process to generate sorbitol from cellulosic sugars needs to be implemented first. Additionally, pathways to produce EG directly from sugars are being investigated. Zhao et al. [339] reported a semi-continuous setup for producing EG from aqueous glucose and dual-functional catalysts. Among the variety of tungsten-based catalysts tested, AMT–Ru/AC reached the highest EG yield (60%). Using a bifunctional nickel tungsten carbide catalysis, Ooms et al. [340] converted concentrated glucose solutions (up to 0.2 kg/L) to EG without loss in selectivity by gradually feeding the sugar solution. The authors proposed that glucose is converted via a retro-aldol reaction into glycol aldehyde, which is further transformed into EG by hydrogenation. The main by-products obtained were sorbitol, erythritol, glycerol, and 1,2-propanediol. Recently, Braskem (Brazil) and Haldor Topsoe (Denmark) have signed a technological cooperation agreement to develop a pioneering route to produce MEG from sugar. The agreement calls for the construction of a demonstration plant in Denmark, with operation slated to begin in 2019. The project is based on the conversion of sugar into MEG at a single industrial unit, via the Monosaccharide Industrial Cracker (MOSAIK), a solution for cracking sugars to an intermediary product which can be further converted to MEG using Haldor Topsoe’s patented processes and catalysts. The overall goal of the partnership is the start-up of a commercial plant in 2023 [341]. While conversion of cellulosic derivate sugars to EG is in an early stage, it is a promising technology to generate value added compounds from lignocellulose.

### Ethylene propylene diene monomer

Ethylene-propylene-diene monomer (EPDM) is mainly used in the automotive and construction industry, as well as in the manufacture of oil additives [342]. According to Lanxess, approximately 5 kg of EPDM rubber is used in a car. EPDM is characterized by very low density, and good resistance to heat, oxidation, chemicals, and weathering, as well as offering good electrical insulation. Moreover, due to the growing automotive and construction industries in China, the demand for EPDM rubber in China, is expected to grow by 5 to 7% annually over the next 4 years [343].

EPDM has been manufactured using vanadium-based Ziegler–Natta catalyst systems. In the 1950s, Ziegler and Natta discovered the ability of a mixture of a transition metal salt mixed with alkyl aluminum to perform olefin homo- and copolymerization. Conventionally, EPDM and ethylene propylene methylene (EPM) copolymers are produced in solution or slurry using Ziegler–Natta catalysts, such as vanadium oxytrichloride (VOCl3), Vanadium acetylacetonate [V(acac)3], and vanadium tetrachloride (VCl4), in combination with an aluminium-based co-catalyst such as diethyl aluminumchloride (DEAC) and/or ethylaluminum sesquichloride (EASC) and/or ethylaluminum dichloride (EADC) [344]. In the solution process, the gaseous monomer (ethylene) is added under pressure to an organic solvent such as hexane, while the EPDM stays in solution as it forms. By contrast, in the slurry or suspension process, the reaction takes place in a solvent in which the resultant EPDM is not soluble. It has been reported that removing the catalyst residue from the polymer is more difficult in the slurry process. However, some reports suggest that the amount of catalyst required in the slurry process is so low that catalyst removal is not necessary [345]. EPDM is typically produced by solution polymerization using the acidic catalyst system VOCl3 and EASC [344]. The discovery of the cationization of the metal center, which may enhance the ability of the M–C bond, gave birth to the cationic Ziegler–Natta. Today, the Ziegler–Natta olefin polymerization is still under intense research in the academic and industrial sectors [346].

Since 2011, the German specialty chemicals group Lanxess produces the world’s first bio-based EPDM rubber under the name Keltan Eco [347]. Lanxess uses the Keltan ACE technology, a catalyst process that reduces the amount of energy required for production, does not need catalyst extraction, produces no catalyst waste, and has the advantage of eliminating chlorine residues which enhance EPDM’s heat aging properties [343, 348]. Keltan Eco is made of ethylene derived from Brazilian sugarcane supplied by Braskem [342]. In 2016, Lanxess reported concerns on the global slowdown and specifically on the Brazilian downturn in car production, which has had a negative impact on the tire production and in the rubber supplier to the industry. In response, Lanxess reported that its facility at Triunfo, Brazil, now mainly produces emulsion styrene butadiene rubber (ESBR) [349]. Technologies to convert ethanol to ethylene and ethylene to EPDM have reached commercial scales. Therefore, the production of EPDM from cellulosic ethanol can be immediately implemented (Fig. 3). Nonetheless, based on the latest Lanxess’ actions and the rubber market state, production of EPDM is currently not economically favorable.

### Furfural

Furfuran is used in the recovery of lubricants from cracked crude, in the production of specialist adhesives, and as a flavor compound [350]. Furthermore, furfural is a key bio-based platform chemical that can be used to replace oil-based chemicals such as furan, furfuryl alcohol, hydroxy furans, furoic acid, 2(5H)-furanone, furfuryl amine, difurfuryl diamines, furanacrylic acid, furylidene ketones, methyl furan, 2-hydroxymethyl-5-vinyl furan, and 2,5-bis(hydroxymethyl) furan [142]. In 2014, the price of furfural was reported to be around $2198/tonne [351, 352]. Since furfural is not produced from fossil feedstocks, current production methods from biomass do not displace production from petroleum [198]. Furfural is produced by acid hydrolysis of the pentosan contained in woody biomass [353]. The major raw materials for the industrial production of furfural are corncobs and sugarcane bagasse. The solid residues, mainly lignin and cellulose, are dried and used as fuel [354]. The first industrial production of furfural was carried out via a batch process developed by the Quaker Oats Company in the early 1920s in Iowa, USA [353]. The current technologies used for furfural production have not been improved significantly since the 1980s [355]. Furfural plants operate at less than 50% yield, need a lot of steam, generate plenty of effluent, and have a high operating cost. Hence, production in the developed world has declined, while production in developing regions is increasing, for example, the simple low cost Chinese plants [350]. As a result, China is the world’s main producer of furfural, in which furfural is produced from corncobs in the Northern provinces. Many small plants and several large ones exist, particularly in the Shandong Province [350]. The other two major commercial producers are Illovo Sugar from the Republic of South Africa and Central Romana from the Dominican Republic [162]. The typical furfural process has two stages: reaction and purification. Biomass is treated in a dilute acid process to generate a pentosane-rich solid [350]. Treated biomass is fed to a series of reactors to be hydrolyzed to pentose sugars which are then dehydrated to furfural using sulfuric acid as the catalyst. Subsequently, the vapor stream from the reactor, which consists of furfural (about 6 wt%), by-products (4 wt%), and water, is liquefied to make secondary steam before being purified [353]. The purification stage is carried out using a distillation process that is very energy intensive due to the heterogeneous azeotrope between furfural and water (35.46 wt% furfural) [356]. Despite its disadvantages, distillation is still used to generate around 80% of the furfural supply, mainly due to its low capital investment, easy implementation, and inexpensive raw materials [353].

Global production of furfural is estimated in 270 ktpy, with three nations accounting for 90% of production: China (accounting for about 70% of the production), the Dominican Republic, and South Africa [198]. Westpro modified the batch Chinese Huaxia Furfural Technology that was largely used to produce furfural. Westpro’s process uses fixed-bed reactors and continuous dynamic refining, which achieves high yields of furfural, including by-products, at low production costs [350]. Huaxia/Westpro plants operating in China consume 25–35 tons of steam per ton of furfural at about a 50% yield of theoretical pentosan content. These plants are estimated to make $1–2 million in profits each year with a significant by-products contribution to revenue [350]. In 1975, Central Romana expanded its furfural plant, based on sugarcane bagasse, to a total capacity of 41 ktpa (La Romana, Dominican Republic). Later in 1995, Central Romana obtained a plant in Geel, Belgium, to produce furfuryl alcohol from furfural [357]. Illovo Sugar produces 20 ktpy of furfural from sugarcane bagasse applying a distillation process for the separation stage at its facility in Sezela, South Africa [357]. Furfural demand in the USA is not expected to grow significantly: thus, the primary market driver for furfural in the USA may be in the production of jet and diesel fuel blendstocks [198].

Until now, the joint production of bioethanol and furfural has not been possible because 40 to 50% of cellulose degrades during the furfural process. However, pathways to co-produce furfural and 2G ethanol are currently under development. For example, Vedernikovs et al. [358] achieved furfural yields of 75% by injecting small amounts of strong acid and applying salts to ensure differential catalysis of hydrolysis and dehydration reactions. By applying a two-step hydrolysis process, cellulose degradation was reduced, providing enough material to sustain bioethanol co-production. Nevertheless, the process’ high temperatures may reduce enzymatic hydrolysis yields. Additionally, furfural carryover could inhibit fermentation. On the other hand, the former Canadian company Lignol Energy Corporation, acquired by the Brazilian Fibria Celulose SA., applied an ethanol–organosolv process to fractionate wood chips and produce furfural from pentoses and other valuable chemicals from the extracted lignin. The downside of using ethanol as a solvent was that it resulted in low furfural concentrations [359]. Therefore, alternative solvents must be explored to maximize furfural production [355]. In a recent study, Farzad et al. [360] performed an economic analysis of the production of ethanol, ethanol-lactic acid, and ethanol-furfural. In this analysis, furfural was produced in the presence of hydrochloric acid catalyst from the hemicellulose fraction isolated in a pretreatment process. Tetrahydrofuran (THF) was added to extract furfural in a two-phase reaction–separation system. The organic solid phase is separated from the aqueous phase by a downstream decanter and sent to the distillation stage for product recovery. The study showed that co-production of ethanol–lactic acid had the highest profitability. In contrast, the furfural production through a biphasic process contributed significantly to the environmental burdens and cost, making it imperative to improve the yield of furfural while avoiding solvent consumption [360]. Research on the production of furfural from cellulosic biomass is undergoing. In 2014, Cai et al. [361] used metal halides with tetrahydrofuran (THF) to enhance the co-production of furfural and 5-HMF from biomass, achieving high yields of furfural (95%) and 5-HMF (51%). More recently, Nhien et al. [353] evaluated a hybrid purification process that combines extraction and distillation to produce furfural from cellulosic biomass. In this study, the authors evaluated various extracting solvents and their economic impact on the process. The results showed that butyl chloride is the most suitable solvent as it saves up to 19.2% of the total annual cost and reduces total CO_2_ emissions by 58.3% when compared to the traditional distillation process. The most immediate opportunity to co-produce ethanol and furfural would be via the 1G ethanol industry. Since the most common feedstock for the commercial production of furfural is corn cobs and sugarcane bagasse, solid residues from corn ethanol facilities and sugar mills could be converted to furfural. In this scenario, cellulosic biomass can be hydrolyzed to sugars which can be then converted to furfural. Nonetheless, for this pathway to succeeded, it is necessary to develop efficient processes to separate pentose from hexose sugars. Another pathway to produce furfural from cellulosic biomass is via the mentioned single-phase conversion of biomass to furfural. While this strategy can reduce capital costs, further research and development is needed to validate and scale up this technology. Thus, the furfural and cellulosic ethanol integration will not be immediate, since it requires extensive research, optimization, and detailed techno-economic analyses to define routes to reduce costs.

### Furfuryl alcohol

Furfuryl alcohol is widely employed in the chemical industry as an additive or solvent in the production of resins, as a chemical intermediate to manufacture lysine, vitamin C, lubricants, and dispersing agents as well as food additives and ingredients [362]. Hydrogenation of furfural produces furfuryl alcohol, which may be further hydrogenated to tetrahydrofurfuryl alcohol (THFA) [162]. World production of furfural is estimated at around 270 ktpa, of which 60–70% is used for the production of furfuryl alcohol [161]. Furfuryl alcohol can be produced from both vapor- and liquid-phase hydrogenation of furfural. The hydrogenation of furfural is conducted at high temperature and pressure, employing a Cu–Cr catalyst, which exhibits a moderate activity toward furfuryl alcohol. Copper chromite has been used in the furan industry for the selective hydrogenation of furfural to furfuryl alcohol for decades [363]. The selectivity of furfural to furfuryl alcohol over copper chromite pretreated at 300 °C was determined to be 70% [362]. Nonetheless, Cu–Cr catalyst’s greatest disadvantage is its high toxicity which causes severe environmental pollution [363]. Therefore, the design of active and selective catalytic systems presents great challenges and is being actively studied [364, 365]. Hydrogenation of furfural in the liquid phase has been studied using catalyst based on Ni, Co, Ru, and Pd, optionally with a second metal or promoter to improve the activity and selectivity [366, 367]. Catalysts based on Ni or Co modified with Cu, Fe, Ce, or heteropoly acids have reached 98% selectivity at almost total conversion: however, these catalysts cannot be reused [368–370].

TransFurans Chemicals is the leading manufacturer of furfuryl alcohol. It operates the world’s largest furfuryl alcohol facility (Geel, Belgium) with an annual output of 40,000 tonnes. This hydrogenation plant has operated since 1972, supplying foundry resin manufacturers with furfuryl alcohol derivate from furfural produced from sugarcane bagasse at Central Romana Corporation’s facility [371]. Other furanic resins such as Biocarb and Biorez formulations and specialty furfural-based chemicals are also manufactured by TransFurans Chemicals [368, 371]. In China, Zibo Shuangyu Chemical produces 5 ktpa of furfuryl alcohol applying the liquid-phase hydrogenation method [371]. Since the furfural market is expected to reach nearly $1.1 billion by 2021 from $625.5 million in 2016 [287], research on furfuryl alcohol is also expected to increase. Nonetheless, production of furfuryl alcohol through the commercialized furfural hydrogenation in a cellulosic ethanol process is limited by the successful separation of pentose and hexose sugars. While production of furfural, hydroxymethylfurfural integrated into a 2G bioethanol process, it can be implemented into a biorefinery that produces 1G ethanol and uses the residual cellulosic waste to generate 2G ethanol and/or furfural, and consequently furfuryl alcohol, in separated processes, adding flexibility to the entire biorefinery.

### Glutamic acid

Glutamic acid is used to produce C5 compounds and their corresponding polymers. The polymer form of glutamic acid, poly-γ-glutamic acid (γ-PGA), has been successfully commercialized. γ-PGA is water-soluble, biodegradable, edible, and non-toxic toward humans and the environment. Therefore, γ-PGA and its derivatives can be used as thickener, humectant, cryoprotectant, drug carrier, biodegradable fibers, highly water-absorbable hydrogels, biopolymer flocculants, and animal feed additives [372]. Monosodium glutamate (MSG) is another largely commercialized glutamic acid derivate. The sodium salt of l-glutamic acid is a popular flavor enhancer and an additive for foods [373]. Certain strains of bacteria, such as *Brevibacterium* and *Corynebacterium,* can produce glutamic acid from different carbon sources: glucose, ethanol, and glycerol [142]. Glucose or hydrolyzed starch is the usual carbon source [373]. The global production capacity of glutamic acid by 2015 was estimated to be around 200 ktpy, in which most of the production was achieved through fermentation using the *coryneform* bacteria [142]. In an early study, Su and Yamada [374] screened various microorganisms capable to producing l-glutamic acid from glucose and nitrogen sources. Five strains belong to the *genus Brevibacterium* were found to have exceedingly high l-glutamic acid productivity. In the 1950s, Dr. Kinoshita discovered that *Corynebacterium glutamicum* is a superior amino acid producer [375]. Since then, a number of fermentation techniques have been used to produce glutamic acid. Tatsuya et al. [376] described a continuous process that increased the production of l-glutamic acid by about twofold the productivity achieved by the fed-batch method (40 h culture time). The outcome of continuing the cultivation for 40 h is such that the yield of l-glutamic acid was 56% and the productivity was 5 g/L/h. Moreover, when compared with the cell recycling culture method, the continuous process maintained the activity of producing l-glutamic acid for a longer time. Recent efforts to improve the glutamic acid fermentation process involve the development of immobilized cell reactors that allow microorganisms recycling. Amin and Al-Talhi [377] entrapped *C. glutamicum* into carrageenan gel beads and used it in the batch, fed-batch, and continuous production of l-glutamic acid from nutritionally enriched sugarcane molasses. However, repeated batch fermentation runs were unsatisfactory. The best results were obtained when the immobilized cell bioreactor was operated in a continuous mode, achieving up to 73 g/L of l-glutamic acid with a yield of 75.7%. Despite the large efforts to enhance the fermentative process, the main limitation to produce glutamic acid at a large-scale is the complex and numerous downstream stages, such as precipitation, conventional filtration, acidification, carbon adsorption, and evaporation. These treatments are essential to obtain high purity glutamic acid but highly costly. Thus, a membrane-based process is envisioned to eliminate the need for separate purification units and to reduce production costs [378].

The world’s largest MSG and xanthan gum producer, Fufeng Group Limited, produces and auto supplies glutamic acid for the production of MSG and xanthan gum at Shandong Province, Shaanxi Province, Inner Mongolia Autonomous Region, and Xinjiang Uygur Autonomous Region of the People’s Republic of China [379]. Fufeng Group ferments cornstarch syrup to generate glutamic acid, threonine, starch sweeteners (maltose, and crystallized glucose), and pharmaceutical amino acids. In 2015, the Fufeng Group produced 767 and 955 ktpy of glutamic acid and MSG, respectively [380]. In the same year, Fufeng Group also announced a new technology for the production of MSG, which reduces the consumption of sugar and liquid ammonia. By 2009, the top eight MSG producers (Fufeng, Meihua, Lianhua, Linghua, Xinle, Yipin, Sanjiu, and Aosang) held about a 49% share of the MSG market and a 70% share of the glutamic acid market. Fufeng, Meihua, and Lianhua had 35.6% of the MSG market and 51.2% of the glutamic acid market in China in 2009 [381]. The risks for these companies lie on the volatility of corn and coal prices and the strict environmental control by the Chinese government. For example, corn kernel accounted for 53% of Fufeng’s FY09 cost of sales [381]. Therefore, the use of cheaper raw materials may reduce the dependency of the glutamic acid industry on the corn sector. A solution to reduce this dependency is through the use of hydrolyzed sugar derivate from lignocellulosic biomass. Das et al. [382] evaluated the production of glutamic acid from pure glucose and palm waste hydrolysate by fermentation with *Brevibacterium lactofermentum* ATCC 13869. Palm waste hydrolysate was prepared by enzymatic saccharification of treated palm press fibers. The product yield obtained for pure glucose was 70 g/L, whereas, for palm waste, hydrolysate was 88 g/L. The higher yield was attributed to the fact that this organism can convert sugars, other than only glucose, present in the hydrolysate. While these results are promising, research on the production of glutamic acid from lignocellulose is very limited. Thus, production of glutamic acid could be integrated into the 1G ethanol process, and into a 2G ethanol process, if glucose can be efficiently separated from the hydrolysate (Fig. 2). Further research and economic analyses are needed to scale up technologies to produce cellulose-based glutamic acid.

### Isobutanol

Isobutanol, 2-methyl-1-propanol or isobutyl alcohol, has a market of 500 ktpy, and it is used as a raw material for isobutyl acrylate, coating resins, isobutyl acetate, and paint thinners. Isobutanol has an estimated price of $1530 per tonne (2010–2014) [205] and it is used as a precursor of various isobutyl esters, chemical intermediate, solvents for paints and coating, pharmaceuticals, and automotive paint cleaner additive [383]. In addition, isobutanol can be blended with gasoline in higher concentrations and used in today’s cars and fuel infrastructure [384]. Since isobutanol can be generated from biomass, it is an attractive alternative to bioethanol. Moreover, isobutanol can also be converted into hydrocarbons to make “green gasoline,” diesel, and jet fuel [385].

From the early 1940s until the early 1980s, isobutanol was produced via the hydroformylation of propylene to butyraldehyde, which was further hydrogenated to isobutanol using a cobalt catalyst system. Different isomeric ratios of butanol were obtained depending on the pressure, temperature, and type of catalyst used. Disadvantages of this process include poor conversion, low selectivity, and high operating pressures [386]. Aiming to solve these issues, the “low-pressure Oxo” process (LP OxoSM process) was developed at the beginning of 1971 by Johnson Matthey’s Process Technologies with its license partner The Dow Chemical Company. Using rhodium-based catalysis, the LP OxoSM process offered major economic advantages and technical simplicity. About two-thirds of the world’s butyraldehyde are now produced via the LP OxoSM process [387]. An alternative process for the production of isobutanol was the Reppe carbonylation, in which propylene, carbon monoxide, and water react under pressure in the presence of a catalyst. This reaction generates a mixture of butyraldehyde and isobutyraldehyde, this last compound is reduced to isobutanol. Due to its high operating cost, this process was not commercialized, [387]. Since 1-propanol, isobutanol, and n-butanol have higher energy density and lower hygroscopicity than ethanol, these alcohols are attractive as gasoline additives or substitutes. However, no native organisms have been identified to produce these alcohols in substantial quantities [388]. A synthetic approach to producing higher alcohols from non-fermentative pathways in *Escherichia coli* has been developed. For example, Atsumi et al. [389] used an *E. coli* strain (JCL260) as a host for isobutanol synthesis. Genes involved in the by-product formation from pyruvate were deleted in JCL260 to increase pyruvate availability for isobutanol synthesis. As a result, 22 g/L of isobutanol was produced in 112 h with a yield of 86% of the theoretical maximum. Isobutanol production can be limited by various factors depending on the selected strain and process. Since isobutanol is toxic to the cell, improving the microorganism’s tolerance is a primary concern to achieve high product titers [388]. An isobutanol-tolerant *E. coli* strain (SA481) was evolved from JCL260 and showed superior growth characteristics compared to the JCL260 strain when cultivated at 6 and 8 g/L of isobutanol [390]. In addition to *E*. *coli*, isobutanol has been produced using *C. glutamicum* through CO_2_ fixation via photosynthesis [391]. An alternative approach that is under development is the consolidated bioprocessing (CBP) where microorganisms hydrolyse and ferment sugars into biofuel within a single process. Minty et al. [392] developed synthetic fungal–bacterial consortia for biosynthesis of valuable products from lignocellulosic feedstocks. The authors achieved titers up to 1.88 g/L of isobutanol and yield up to 62% of theoretical maximum. Further exploration of microbial cellulose utilization is necessary to develop and upscale the CBP system.

In September 2010, Gevo (Englewood, CO, USA) acquired a facility located in Luverne, Minnesota, USA, and subsequently retrofitted it to produce isobutanol. The plant operates in a side-by-side mode, producing both ethanol and isobutanol. Gevo produces isobutanol by fermentation and is currently optimizing its technology. Gevo expects the earnings before interest, taxes, depreciation, and amortization (EBITDA) profit margin for isobutanol to be approximately $0.50 to $1.00 per gallon [393]. Since 2011, Gevo operates a biorefinery at South Hampton Resources’ facility in Silsbee, Texas, USA. In this facility, Gevo converts isobutanol into hydrocarbon products such as jet fuel, isooctane, and ingredients for polyester. The facility has an input capacity of approximately 5–10 thousand gallons of isobutanol per month [394]. During 2011, Gevo announced a ground-breaking agreement with The Coca–Cola Company to create renewable paraxylene from plant-based isobutanol [395]. As a result, in 2013, Gevo added the capability to produce paraxylene to its biorefinery. Moreover, Gevo reported a revenue of $5.6 millions and a gross loss of $3.8 million in 2017. The company described plans to convert its biorefinery, which produce approximately 100,000 gallons of isobutanol, to exclusively produce isobutanol and hydrocarbon products [396]. Butamax Advanced Biofuels (Wilmington, DE, USA), a joint venture of BP and DuPont, developed a bio-isobutanol technology to convert sugars from various biomass feedstocks such as corn and sugarcane [397]. Butamax has purchased Nesika Energy (Scandia, KS, USA), which operated a corn-based ethanol plant in Scandia, Kan, USA. Butamax plans to convert part of the Scandia facility to make isobutanol and the facility is expected to be in place by 2019 [397]. Gevo has adapted its technology to convert cellulosic sugars derived from wood waste into renewable isobutanol (Fig. 2), which is then further converted into Gevo’s Alcohol-to-Jet (ATJ) fuel. The Northwest Advanced Renewables Alliance (NARA) in WA, USA, supplied the sugars that were derived from forest residuals to Gevo, which produced cellulosic isobutanol at its demonstration facility in St. Joseph, MO, USA. Next, isobutanol was further converted to ATJ in Gevo’s biorefinery in Silsbee, TX, USA [397]. Co-production of ethanol and isobutanol from lignocellulose is possible; however, its economic viability is to be demonstrated. While Gevo’s technology to produce isobutanol from corn starch-based sugars is commercially available and under optimization [393], the production of cellulosic isobutanol is at a demonstration scale. Thus, production of cellulosic isobutanol requires further optimization before it can be commercialized.

### Itaconic acid

Itaconic acid, also known as methyl succinic acid, is a granulated light-yellow powder that has the potential to be a key building block for deriving commodity and specialty chemicals. Itaconic acid is mainly used in the production of lubricant additives, surface active agents, dye intermediates, plastics, synthetic rubber and resins, and chemical fibers. With an estimated price of $1900/tonne, the global itaconic acid market in 2015 was 41 ktpa with sales of $79 million [162]. As only a few end-use applications with high-volume markets have been identified, but not developed until recently, itaconic acid has only a niche market [163]. Itaconic acid can potentially replace acrylic acid (in the superabsorbent polymers), acetone cyanohydrin (in the production of methyl methacrylate), maleic anhydride (in the production of unsaturated polyester resin), and sodium tripolyphosphate (in the production of phosphate-free detergent builders) [213]. The most promising application for itaconic acid is in the production of methyl methacrylate (MMA), which is the most important ester of methacrylic acid and it is also the monomer for polymethyl methacrylate (PMMA) polymers and copolymer. If the price of itaconic acid was competitive with the price of acetone cyanohydrin, it could be the preferred raw material for the MMA production. The projected market for itaconic acid is estimated in 408 ktpa with a value of $567.4 million for 2020, a feasible scenario for MMA production. If the price of itaconic acid is not competitive for MMA production, the market size of itaconic acid in 2020 will not exceed 197 ktpy with a value of $315 million [213].

Itaconic acid was first produced by distillation of citric acid. However, since the 1960s, itaconic acid has been produced by fermentation of carbohydrates, mainly commercially produced using *Aspergillus terreus* via submerged fungal fermentation, Fig. 2 [398]. Glucose is widely used in the production of itaconic acid. Fermentation of other sugars to itaconic acid results in low yields: 18%, 31%, and less than 1% using arabinose, xylose, and lactose, respectively [399]. Fermentation times for itaconic acid generation range from 2 to 14 days, with an optimum of 7 days at around 37 °C [400–402]. Intensive research to reduce the production cost of itaconic acid is undergoing. Yahiro et al. [403] mutated the *A. terreus* strain IFO6365, capable of producing 82 g/L of itaconic acid within 6 days of fermentation. Li et al. [398] expressed the CAD gene from *A. terreus* in *A. niger* to produce itaconic acid using *A. niger*. However, the performance of the genetically modified microorganism was found not to be beneficial for itaconic acid production. Itaconic acid fermentation using immobilized microorganisms has also been investigated. By immobilizing *A. terreus* on polyurethane foam, Vassilev et al. [404] obtained an average yield of 15.1 g/L itaconic acid in four repeated batch fermentations.

In the past, itaconic acid was mainly manufactured in the USA, China, Japan, and France. Cargill and Pfizer were market leaders, while French Rhodia and Japanese Iwata Chemicals had strong market positions. Currently, the main producers of itaconic acid are in China: Qingdao Kehai Biochemistry (global leader), Alpha Chemika, Zhejiang Guoguang Biochemistry, and Jinan Huaming Biochemistry. Nonetheless, WEASTRA reported that even when Chinese companies claim to be producers of itaconic acid, in reality, most of them have closed down their production due to the low demand and became only distributors of itaconic acid [213]. Except for Chinese producers, the Indian manufacturer Alpha Chemika is also a leader of the market, with a capacity of 8 ktpy [213].

The highest itaconic acid yield is obtained when glucose is used as a substrate. However, to reduce the production cost of itaconic acid, alternative low-cost raw materials should be considered. Cheaper materials than glucose, such as starch, molasses, hydrolysates of corn syrup or wood, have been investigated to produce biochemicals. However, the literature on the conversion of cellulosic sugars to itaconic acid is very limited. Jiménez-Quero et al. [405] reported a maximum itaconic acid yield of 0.14% when performing a liquid-state fermentation of corn cob hydrolysates (1.9% total glucose) using *A. oryzae*. The authors reported that two strains of *A. terreus* (named DSM 826 and DSM 62071) could not grow at all in wheat bran and corn cobs hydrolysates. In contrast, a yield of 0.11 mg itaconic acid per gram of biomass (corn cobs), the highest reported in the literature for simultaneous solid-state fermentation without sugar supplements, was achieved using *A. oryzae* [406]. Considering the itaconic acid’s niche market, the fact that downstream conversion to MMA is not yet commercial, the need to lower production costs, and the required research to use cellulosic sugars, the production of itaconic acid as part of a 2G ethanol process is not attractive. Similar to the 1,2 butanediol case, a biorefinery that generates 1G and 2G bioethanol may beneficiate from using a fraction of the sugar-based crops (e.g., corn starch or sugarcane molasses) to produce itaconic acid.

### Lactic acid

Lactic acid (LA) is a carboxylic acid that has been extensively studied and successfully commercialized. It is present in many foods both naturally or as a product of microbial fermentation, for example, in yogurt, buttermilk, sourdough breads, and other fermented foods [407]. LA has also been used in the food and beverage sector as a preservative and pH adjusting agent. In the pharmaceutical and chemical industries, it is used as a solvent and a starting material in the production of lactate ester [161]. By 1990, LA’s worldwide production volume was approximated to 40 ktpy, while the current worldwide production (including polymer uses) is estimated to be around 120 ktpy. LA’s price depends on the market and ranges between $1.30 and $2.30/kg [407].

LA can be produced via fermentation of glucose and sucrose by lactic acid bacteria, as shown in Fig. 2, or chemical synthesis, which have been used for commercial production in the past. In the chemical synthesis, hydrogen cyanide is added to acetaldehyde in the presence of a base at high pressures in the liquid phase to generate lactonitrile. The crude of lactonitrile is recovered by distillation and hydrolyzed, either by concentrated H_2_SO_4_ or by HCl, to LA and ammonium salt. Next, LA is esterified with methanol to produce methyl lactate, which is distilled and hydrolyzed with water in the presence of an acid catalyst to produce methanol and lactic acid. This process results in a racemic mixture of _DL_-lactic acid [408]. The chemical route had major limitations due to the generation of by-products, inability to produce a stereoisomer of either _D_-(−)- or _L_-(+)-lactic acid, and high manufacturing costs [407]. Consequently, the fermentation process is preferred, and about 90% of lactic acid in the world is produced by this method [409]. The _D_-(−)-isomer is often blended with the _L_-(+)-isomer to create polylactic acid (PLA) copolymers with desirable properties like a thermal stability that exceed those of a pure, single isomer PLA product. PLA is expected to be the biggest driver for growth in lactic acid demand [198]. Commercially, production of LA uses different carbohydrates as raw materials: glucose, sucrose, lactose, and starch/maltose derived from different feedstocks, such as sugar beet, molasses, whey, and barley malt [408]. The commercial production processes use homolactic microorganisms such as *Lactobacillus delbrueckii, L. amylophilus, L. bulgaricus*, and L. *leichmanii*. Mutant fungal strains of *A. niger* are also used [407]. Fermentation is conducted in a batch or fed-batch mode, with a reaction time of 2 to 4 days that results in lactate yields of approximately 90% (wt). Excess calcium hydroxide/carbonate is added to the reaction to neutralize the acid, maintaining the pH around 5 to 6, and produce a calcium salt of the acid. The calcium lactate-broth is filtered, carbon treated, evaporated, and acidified with sulfuric acid to convert the salt into lactic acid and insoluble calcium sulfate, which is removed by filtration. The filtrate can be further purified using carbon columns, ion exchange, and evaporation to produce technical-grade LA. To obtain the high-purity, heat-stable product required for the stearoyl lactylates, polymers, solvents, and other value-added applications, technical-grade LA is esterified with methanol or ethanol, and the resulting ester is recovered by distillation, hydrolyzed with water, evaporated, and the alcohol recycled [407].

In the USA, Sterling Chemicals manufactured LA as a by-product of the acrylonitrile process using the chemical route. In Japan, Musashino Chemical used this technology for some of its production, but has recently switched to a fermentative process [407]. The Dutch company Corbion Purac is the world leader in lactic acid production operating 5 plants in the USA, the Netherlands, Spain, Brazil, and Thailand. The last one is the largest plant, with a capacity of 100 ktpy [161]. Recently, Total Chemicals (Houston, USA) and Corbion Purac formed a 50/50 joint venture, Total Corbion PLA, to produce and market polylactic acid (PLA) polymers. Total Corbion PLA planed to build a PLA polymerization plant with a capacity of over 75 ktpy at Corbion’s site in Thailand, which already has a lactide monomer production unit [410]. At the end of 2018, Total Corbion PLA announced the start-up of its PLA bioplastics plant which has successfully produced Luminy^®^ PLA resins [411]. Galactic (Belgium) produces LA and lactides from multiple plants in Europe (30 ktpy), Asia, and America (15 ktpy) [162]. Since 2012, Jungbunzlauer has been operating a lactic acid plant in Marckolsheim, France [410]. Moreover, Taylor et al. [162] reported other LA producers: Glycos Biotechnologies (~ 0.1 ktpa, USA), Henan Jindan Lactic Acid Technology (100 ktpa—the largest in Asia), Chongqing Bofei Biochemical Products (~ 75 ktpa, China), Unitika-Terramac (5 ktpa, Japan), Nantong Jiuding Biological Engineering (1 ktpa, China), Shanghai Tong-jieliang Biomaterial (0.3 ktpa, China), Piaoan Group (10 ktpa in planning, China), Toray Industries (5 ktpa, South Korea), Teijin Limited (1.2 ktpa, Japan), Mitsui Chemical (Japan), and Purac-Toyobo (Japan).

The main barrier to lower the production cost of LA is the cost of raw materials, e.g., starch and refined sugars. Therefore, low-cost and non-food lignocellulosic materials may allow the reduction in LA production costs. However, similar to the 2G ethanol process, lignocellulose requires a series of processing steps to convert structural carbohydrates to fermentable sugars. The conventional process to produce LA from lignocellulose is comparable to that of 2G bioethanol: pretreatment, enzymatic hydrolysis, fermentation, and separation. Thus, cellulosic LA faces similar barriers: recalcitrant lignocellulose, co-fermentation of hexo- and pento-sugars, and costly separation process. Fermentation of lignocellulose-derived sugars from materials, such as cottonseed, corncob, stalks, wood, beet molasses, or sugarcane bagasse, has been reviewed by Abdel-Rahman, Tashiro, and Sonomoto [412]. The review addresses the fermentation optimization by pH control, reactor type, and engineered strains. As the research on fermentation of lignocellulose-derived sugars continues, new process arrangements and strains are being developed. For example, by using the thermophilic strain *Bacillus sp.* NL01, Ouyang et al. [413] fermented corn stover-derived glucose under open condition without sterilization in a batch reactor. A concentration of 56.37 g/L LA was obtained from the lignocellulosic hydrolysate, which contained solid residues. In a fed-batch fermentation, 75.03 g/L LA was obtained from the lignocellulosic hydrolysate supernatant with a yield of 74.5%. *Lactobacillus paracasei* 7BL has shown a high tolerance to inhibitors and the ability to produce optically pure _L_-lactic acid after the interruption of ldhD gene achieving a high titer of _L_-lactic acid (215 g/l) by fed-batch strategy. In addition, 99 g/L of LA with high yield (0.96 g/g) was obtained by using non-detoxified wood-derived sugar hydrolysate [414]. Ahring et al. [415] fermented clarified corn stover hydrolysate using a strain of *Bacillus coagulans* (strain AD) in a continuous fermentation. Maximum LA yield was found to be 1.09 g/g biomass sugars at pH 6.0 and a residence time of 24 h. A major barrier to viable lignocellulosic LA production is the inhibitory effect of the by-products formed during the pretreatment stage. Research on lignocellulosic LA is far advanced, but its industrial feasibility is to be demonstrated. Although pilot plant tests and economic analyses are needed to implement this process into a cellulosic biorefinery, its advanced technology and growing market point out to a fast industrialization process.

### Lactide

Lactide can be converted into a wide range of intermediates such as acrylic acid, propylene glycol, 2,3-pentane-dione, acetaldehyde, pyruvic acid, and lactide. Lactide can be also used in the food industry as a preventive agent against thermal decomposition of bisphenol; food additive for the conservation of milk and meat-based products; pH regulator, or coagulation agent for tofu, soybeans, and dairy products. In the industry, lactide is used as a reagent for chemical reactions without water molecule production (amidation, transesterification, ring opening polymerization), destabilizing agent in the production of porous ceramics, anti-yellowing agent for textiles, and combustion improvement agent for coal and oil [416]. Lactide is a cyclic ester of two lactic acid molecules and the most important building block in the production of PLA, one of the key drivers for lactic acid market growth [417]. As mentioned early, LA has two enantiomeric forms, while lactide has two asymmetric carbon atoms, so it can be found in three steroisomeric forms: l-lactide in which possesses the L (or S) configuration; d-lactide in which possesses the D (or R) configuration; and meso-lactide in which one asymmetric atom has the L configuration and the other has the D configuration. Enantiomeric lactide, especially l-lactide, is used to produce polymers [418]. Each of the mentioned lactides is synthesized by depolymerization of the corresponding oligo (lactic acid) (OLLA) obtained by polycondensation of lactic acid (Fig. 2). Lactide is generated through the back-biting mechanism involving the –OH terminals of OLLA as the active site. This reaction is catalyzed by tin powder, tin halides, tin metal, tin carboxylates, tin alkoxides, compounds involving Sn, Zn, Al, and Sb ions, among others. This monomer can be easily purified by vacuum sublimation to remove water and acid impurities [418].

Commercially, Total Corbion PLA, a joint venture between Total Petrochemicals and Corbion, manufactures lactide monomers: PURALACT^®^, and PURALACT L [419]. Total Corbion PLA produces lactic acid, lactic acid derivatives and lactides (including lactide resins for high performance PLA bioplastics). As mentioned, Corbion operates 5 plants in multiple countries [162] and a new PLA polymerization plant in Thailand [420]. While lactide is mainly used in the production of PLA, it also has multiple industrial applications. Thus, lactide production from cellulosic LA could benefit the economy of a cellulosic biorefinery. As a LA derivative, lactide successful integration into a biorefinery would depend on the viability of producing cellulosic LA. However, if 1G bioethanol and 2G bioethanol are successfully co-produced, starch- or molasses-derived sugars could be used to generate LA, lactides, and PLA, while lignocellulose-derived sugars could be used to cover the ethanol demand.

### Lysine

l-Lysine is an essential amino acid that is not available in sufficient amounts in feed-stuffs to meet the nutritional requirements of animals and humans [421]. Animal feed, which is typically based on corn, wheat or barley, is poor in lysine. Thus, lysine is supplemented to optimize animal growth [422]. With the increasing consumption of white meat in the world, the demand and marked of l-lysine have also grown. However, the production of biomass-based nitrogen-containing bulk chemicals is less developed compared to oxygenated bulk chemicals such as glycols. Global lysine market in animal nutrition was estimated at over 1.7 million tonnes in 2014 and is likely to reach over 2.7 million tonnes by 2023 [423].

In the 1950s at Kyowa Hakko Kogyo Co. in Japan, the bacteria *C. glutamicum* was found to produce large amount of glutamic acid and lysine [161]. *C. glutamicum* has been optimized mostly by repeated random mutation and selection [424]. Using classically derived strains, conversion yields of up to 50% of the theoretical maximum and lysine·HCl titer above 100 g/L have been achieved [425]. Classical mutagenic procedure has improved the production yield; however, the unavoidable accumulation of side-mutations has resulted in growth deficiencies, low stress tolerance, and by-product formations [426]. A milestone in lysine research was the sequencing of the *C. glutamicumand* genome, which provided a large understanding on the biology of the microorganism and the metabolic production of lysine [421]. Ohnishi et al. [427] developed a high lysine-producing mutant of *C. glutamicumand* by “genome breeding” that produced 85 g/L of lysine within 28 h. More recently, Becker et al. [428], developed a strain of *C. glutamicumand* by metabolic engineering of the wild type. By implementing 12 defined genome-based changes in genes encoding central metabolic enzymes, the engineered *C. glutamicum* strain was able to produce lysine with a yield of 0.55 g per gram of glucose, a titer of 120 g/L lysine, and a productivity of 4.0 g/L/h in fed-batch culture. Research on lysine-producing microorganisms is in constant growth and with the development of new genetic techniques, more efficient microorganisms are expected to be developed.

Industrial production of lysine involves the fermentation process and downstream processing. The most commonly raw materials used to produce lysine are cane molasses, beet molasses, sucrose, and dextrose (from hydrolyzed starch). One of the routes used for the downstream process comprises vacuum filtration, evaporation, and spray drying. Alternatives for downstream processing vary depending on the lysine final preparation. In the past, the fermentation broth was separated by ion exchange, followed by addition of HCl, evaporation, and drying [422]. The resulting crystalline lysine-HCl is less hygroscopic than the corresponding sulfate salt and was the major lysine form produced [425]. However, different lysine preparations have been manufactured: liquid lysine (50% purity), granulated lysine sulfate (40–50% purity), or liquid lysine sulfate (20–30% purity) [422]. Evonik is the only company that produces and markets all four essential amino acids for modern animal nutrition, including Biolys^®^ (source of l-lysine), MetAMINO^®^ (dl-methionine), ThreAMINO^®^ (l-threonine), and TrypAMINO^®^ (l-tryptophan) [429]. Evonik produces Biolys^®^, a source of l-lysine, in its 280 ktpy plant in Nebraska, USA, using agricultural products as raw material [429]. In 2016, Evonik commissioned a new plant to produce Biolys^®^ in the Brazilian town of Castro in the state of Paraná. The new plant has an annual production capacity of 80,000 tonnes and started to operate in 2017 [430]. Like other bio-based chemical processes, carbon source represents the major cost in the production of lysine. During fermentation, *C. glutamicum* uses glucose from starch hydrolysis or fructose and sucrose from molasses. Almost all studies on the metabolic production of lysine have been focused on glucose. Therefore, most of our knowledge about the physiology of *C. glutamicum* is based on the metabolism of glucose [431]. *C. glutamicum* can grow aerobically on a variety of sugars (e.g., glucose, fructose, sucrose, ribose, or maltose), alcohols (myo-inositol and ethanol), or organic acids (acetate, propionate, pyruvate, L-lactate, citrate, and L-glutamate) as sole or combined carbon and energy sources [432]. Moreover, this microorganism has shown to withstand pretreatment-derived inhibitors like furfural, hydroxymethylfurfural, and 4-hydroxybenzaldehyde [433]. The downside is that *C. glutamicum* wild type is unable to utilize the pentose sugars xylose and arabinose. Therefore, research on the utilization of cheaper carbon resources, such as lignocellulose, has explored the use of engineered microorganisms capable of using pentoses, which account for about one-fourth to one-third of lignocellulosic hydrolysates [434]. Gopinath et al. [432] prepared a recombinant pentose-utilizing strain derived from the l-lysine-producing *C. glutamicum* strain DM1729. The recombinant strain grew to higher biomass concentrations and produced more l-lysine than the control strains, which utilized only the glucose fraction. Glucose was co-utilized with arabinose and xylose by the recombinant strain when the substrates were present as pure chemicals and when present in acid hydrolysates from agricultural residues. The recombinant strain produced up to 6.14 ± 0.3 g/L l-lysine on media containing rice straw or wheat bran-acid derived hydrolysate. These results revealed the potential use of agricultural waste materials as alternative feedstock for lysine production. Growth and substrate utilization were slower in media based on the acid hydrolysates, which might be explained by the presence of growth inhibitors. Therefore, a more immediate route to implement lysine production into a cellulosic biorefinery would be by reducing the inhibitors in the hydrolyzed. This can be achieved through a detoxification process involving the adsorption of inhibitors onto carriers like activated carbon and other synthetic resins. For example, Christopher et al. [151] fermented detoxified acid pretreatment liquor to generate lysine using a mutant strain of *C. glutamicum*. At 72 h of fermentation, the engineered *C. glutamicum* grown on detoxified pretreatment liquor producing 4.39 g/L l-lysine. Thus, production of lysine as part of a cellulosic ethanol process, similar to the itaconic acid case (Fig. 1), could be carried out by two routes: using glucose derivate from molasses and/or starch or from cellulose. To efficiently convert cellulosic derivate sugars to lysine, a detoxification process must be implemented, which will increase the capital cost of the venture. Therefore, detailed economic analyses and optimization of separation/purification processes are required to determine the potential benefits of producing lysine from cellulosic sugars.

### Microfibrillated cellulose

Cellulose can be converted to different micro- and nanostructures with a variety of physical properties, depending on the origin of cellulose and production method. Cellulose particles with at least one dimension in the nanoscale (1–100 nm) are referred to as nanocellulose [435–437]. Osong et al. noted, in a review about microfibrillated and nanofibrillated cellulose, that there is still a lot of confusion regarding the terminology and nomenclature of nanocellulose [438]. Depending on the production conditions, nanocellulose from biomass can be classified into two categories: (i) cellulose nanocrystals (CNC) or cellulose whiskers, and (ii) microfibrillated cellulose (MFC) and nanofibrillated cellulose (NFC)—sometimes also referred to as MFC due its micrometer range length, or cellulose nanofibers. Due to various terminologies used to describe cellulose nanomaterials, various organizations have initiated a process to standardize the nanocellulose terminology. Nonetheless, this work has not been finalized yet.

MFC was introduced in 1983 when cellulose with lateral dimensions in nanometer range was produced by passing a softwood pulp aqueous suspension several times through a high-pressure homogenizer. During this process, networks of nanofibrils are produced due to high shearing forces [435, 436]. Microfibrils are 10–100 nm thick with a length of several µm, and can be regarded as nanofibers. Their strength, flexibility, and aspect ratio open possibilities to utilize MFC in large-scale applications, e.g., nanocomposite, packaging, coating, and dispersion technology [439]. In the paper industry, the addition of MFC in with papermaking suspensions improves the overall strength of paper, reduces its porosity, and decreases density [436]. MFC’s applications in polymer reinforcement and anti-microbial films are expected to hit the market soon, and thus, the overall MFC’s market is projected to be worth $250 million in North America by 2020 [440].

MFC production methods usually comprise intensive mechanical treatment. However, according to the degree of processing and raw material, pretreatments are performed before mechanical defibrillation. Several processes can be used to produce MFC, e.g., successive refining, enzymatic hydrolysis, again refining, and finally homogenization [435]. Pretreatments of cellulosic fibers promote the accessibility of hydroxyl groups, increase the inner surface, alter crystallinity, and break cellulose hydrogen bonds, boosting fiber’s reactivity [441]. Mechanical treatments can be divided into refining and homogenizing [442], microfluidization [443], grinding, [444], cryocrushing [445], and high intensity ultrasonication [446]. Depending on the kind of feedstock, mechanical treatments alone have the disadvantage of consuming large amounts of energy [447]. For example, Eriksen et al. [447] reported that pretreatment processes with enzyme or chemical can reduce energy consumption to an amount of 1000 kWh/ton from 20,000 to 30,000 kWh/ton of cellulosic fibers. However, Spencer et al. [448] estimated that the energy required to produce MFC from bleached kraft hardwood pulps by micro-grinding, no pretreatment, was 1550 kWh/ton. Thus, while pretreatments may reduce energy demand, post-treatments, mainly surface chemical modification, are performed to endow MFC with new properties or to conserve their intrinsic characteristics. The MFC surface chemical modification can include treatments such as physical adsorption, molecular grafting, or polymer grafting [435].

In the industrial sector, Borregaard (Norway), through its proprietary technology, Exilva, constructed the world’s first commercial-scale production facility for Exilva MFC in Sarpsborg, Norway. The plant has a capacity of 10,000 tons of 10% paste (1000 dry tons) per year and started operation in 2016. The factory uses Norwegian spruce as raw material. Additionally, Borregaard operates a demonstration plant with a capacity of 45-63 tons of 10% paste per year [440, 449]. FiberLean Technologies, a Joint Venture between Imerys (France) and Omya (Switzerland), focuses in the industrialization of nanocellulose. FiberLean produces FiberLean^®^ MFC, a composite of MFC and mineral. Application of FiberLean^®^ MFC in the paper industry typically allows replacing 10-15% fiber. FiberLean operates a plant with a capacity of 8 ktpy of MFC, equivalent to 40 ktpy of FiberLean^®^ MFC composite [440]. FiberLean^®^ MFC is claimed to be established and proven to be a cost-efficient way for papermakers to become more competitive [440]. Kruger Biomaterials Inc., a Canadian company, operates, since 2014, a commercial plant in Trois-Rivières, Canada, with an annual production capacity of up to 6000 tonnes of FiloCell, cellulose filaments, derived from FSC^®^-certified kraft wood pulp. Kruger Biomaterials’ process requires no chemicals or enzymes and produces no effluent. The yield from this process is claimed to be 100% [450]. In 2017, Stora Enso (Finland) declared to invest a total of EUR 9.1 million in the consumer board mills in Imatra and Ingerois, Finland, and Fors, Sweden, to continue the commercialization of microfibrillated cellulose (MFC) and to accelerate product development. The plants are scheduled to start production by the end of 2017 and expected to reach full production within 3 to 5 years [450]. In Japan, Chuetsu opened a commercial cellulose nanofiber (CNF) plant with a capacity of 100 tonnes/year its Kawachi plant in Kagoshima prefecture. In December 2016, Nippon Paper announced a demonstration plant at Fuji Mill (Fuji, Shizuoka). In July 2017, the company announced the completion of the construction of a demonstration plant for cellulose nanofiber (CNF)-reinforced plastic at the Fuji Mill (Fuji, Shizuoka). The facility produces CNFRPs by mixing CNF and plastics. The company also produces CNF at the Ishinomaki mill since April 2017. The facility is the world’s largest for CNF with a capacity of 500 tons per year of TEMPO ((2,2,6,6-tetramethylpiperidin-1-yl)oxyl) oxidized CNF [451].

**Fig. 5:**
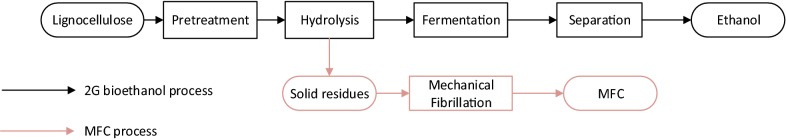
Diagram for the co-production of 2G ethanol and MFC from lignocellulose

The solid residues produced after the enzymatic hydrolysis of lignocellulose during the production of 2G ethanol are normally proposed to be burned to produce steam and energy. Nonetheless, these residues contain a considerable amount of recalcitrant cellulose, and thus, they may be suitable as a raw material to produce MFC, as shown in Fig. 5. Moreover, extraction of MFC from these solid residues are expected to be smoother than from raw materials as these residues have been already pretreated and hydrolyzed. This concept has been explored in various publications where MFC was obtained from 2G bioethanol’s solid residue. For example, Zhu et al. [452] used commercial enzymes to fractionate the less recalcitrant amorphous cellulose from a bleached kraft eucalyptus pulp, resulting in a highly crystalline and recalcitrant cellulose (RC). The RC is difficult to hydrolyze to sugars but very suitable for producing nanocellulose through mechanical homogenization. The hydrolyzed sugars were fermented to ethanol with an efficiency of 92%. Enzymatically fractionated fibers were refined to nanocellulose with a diameter of about 20 nm and lengths of 500 nm or longer, defined by the authors as nanofibrillated cellulose (NFC). The produced nanocellulose had an average fiber length of about 200 µm, and was used to generate nanocellulose films which were optically transparent and with opacity as low as 12%. The films were mechanically strong and stiff, with tensile strengths and moduli of approximately 10 and 6 times higher than those of the film made from fibers that had not been nanofibrillated. In another study, Herzele et al. [453] isolated MFC derived from bleached pulp and from pulp, termed microfibrillated lignocellulose (MFLC), with high residual lignin content. The microfibrillated material was used as filler to produce cellulose-reinforced polycaprolactone nanocomposite film. Overall, the performance of MFLC filler resulted in more favorable nanocomposite tensile performance than the MFC derived from bleached pulp. While the technical feasibility of producing MFC from 2G ethanol’s solid residues has been demonstrated at bench scale, demonstration plants and economic analyses are required to assess the true benefit of co-producing MFC. Since the process to produce MFC is technically mature, and because it can be easily integrated into a 2G ethanol plant, production of lignocellulose-based MFC is an attractive and short-term opportunity to increase 2G ethanol’s profitability.

### Polyethylene

Polyethylene (PE) is primarily used in plastic bags, plastic films, geo-membranes, bottles, and tubes. Moreover, PE is also used in the automobile and pharmaceutical industries with a reported price of $1676 per tonne (2010-2014) in northwestern Europe [162, 205]. Its mechanical properties depend significantly on variables such as the extent and type of branching, crystal structure, and molecular weight. The main types of PE are high-density PE (HDPE), low-density PE (LDPE), and linear low-density PE (LLDPE) [161].

Polyethylene is usually made by dehydrating ethanol to ethylene and subsequently polymerizing the ethylene (Fig. 3). Fossil ethylene is derived from either modifying natural gas or from the catalytic cracking of crude oil [454]. PE from renewable raw materials can be made by dehydrating bioethanol to ethylene, and subsequently polymerizing the ethylene. Therefore, biomass such as sucrose, starchy, and lignocellulosic feedstock can be used to produce bio-based PE [455]. Fossil PE has a production volume of 88 million tonnes, while production of bio-based PE is about 200,000 tonnes [162, 338]. As a drop-in equivalent, bio-based PE has an identical chemical structure to fossil PE. Therefore, bio-based PE can be recycled using the current waste separation system, and processed into new bio-based PE products using conventional technologies [162]. A study on the production of bioplastics, such as polyethylene, from bioethanol-based ethylene concluded that the ethanol conversion and the reaction selectivity have a direct impact on the production cost [334, 455]. While bio-based PE production costs are not available publicly, the cost of bio-based PE is expected to depend on the production costs of bioethanol and biomass feedstock prices. Bio-ethylene cost in Brazil and India is typically $1200/tonne (from sugarcane) and, in China (using sweet sorghum), is around $1700/tonne. In contrast, in the USA, bio-ethylene cost (from corn) is reported at about $2000/tonne, and in Europe (from sugar beet) around $2600/tonne [162]. In a recent techno-economic analysis of a bio-ethylene plant using lignocellulose as feedstock, Mohsenzadeh et al. [334] concluded that impurities in the ethanol feed have no significant effect on the quality of the produced bio-ethylene. However, the economic evaluation showed that the process is not profitable at the current raw materials and products’ prices.

Braskem manufactures bio-based PE, from ethanol sugarcane, under the name of I’m green™ Polyethylene. Braskem’s polyethylene family, named I’m green™, includes HDPE and LLDPE. Starting in January 2014, the LDPE family was added to the product portfolio. Braskem’s green ethylene plant was commissioned in September 2010, and has an annual production capacity of 200 ktpy of I’m green™ Polyethylene [331]. In 2007, Dow and Mitsui formed a joint venture to build and co-own a 240 ktpa ethanol plant at Dow’s existing sugarcane operation in Santa Vitória, Brazil. The second phase of the project, a 350 ktpa of bio-based PE production plant, was put on hold [162]. In 2015, Mitsui sold its entire shares to the Dow Chemical Company [456]. With the PE’s technology at an industrial scale, the real barrier to the bio-based PE production is the price difference between fossil- and bio-based. As a result, bio-based PE has succeeded only in Brazil, where low-cost feedstock is available. Thus, the economic viability of producing PE from bio-ethylene will depend on the feedstock prices, bioethanol production costs, and the fossil-based PE’s prices.

### Polyethylene glycol

Ethylene is commercially oxidized to ethylene oxide (EO) and alternatively hydrolyzed to ethylene glycol (EG). Polyethylene glycol (PEG), also known as polyethylene oxide, is a linear or branched polyether terminated with hydroxyl groups. It can be synthesized by anionic ring-opening polymerization of ethylene oxide initiated by nucleophilic attack of a hydroxide ion on the epoxide ring [457]. Due to the unique ability of PEG to be soluble in both aqueous solutions and organic solvents, it is suitable to produce copolymers with different architectures, e.g., linear, branched, star-shaped, and comb-like PEGs [457]. For example, polytrimethylene ether glycol can be prepared by dehydration of 1,3-propanediol or by ring opening polymerization of oxetane, typically using an acid catalyst [457]. Moreover, PEG-based polymers can be developed via the PEGylation method, the process of covalent attachment of one or more PEG chains to another molecule [142]. Thus, PEG can be used in the medical sector, for example, as a polymer-based drug delivery [457].

PEGs can be produced via mild polycondensation of 1,3-propanediol (1,3-PDO) using an acid catalyst at 120–180 °C under an inert nitrogen reactor blanket (Fig. 2) [458]. Since 2008, DuPont has manufactured polyether polyol liquid glycols (PEGs) using bio-based 1,3-PDO as feedstock. DuPont’s 1,3-PDO is produced via corn sugar fermentation using a genetically modified *E. coli* developed by DuPont in partnership with Tate & Lyle. DuPont’s commercial 1,3-PDO plant (140 ktpy capacity) is located in Loudon, Tennessee, USA. This 1,3-PDO is shipped to DuPont’s First Mississippi subsidiary (Pascagoula, Mississippi, USA) and to Ontario, Canada, for polymerization to PEG [199].

As mentioned earlier, inhibitors are a major challenge to the viable conversion of sugars to chemicals. Thus, in a cellulosic biorefinery, 1,3-PDO could be produced from molasses and starch, similar to DuPont’s arrangement, or from cellulosic hydrolyzed sugars by implementing detoxification processes. Alternatively, microorganisms able to withstand high inhibitors concentration could be engineered to convert cellulosic derivate sugars to 1,3-PDO, which can be further converted to PEG. As our understanding about the fermentative pathways to produce bioproducts increases, development of engineered microorganisms is becoming more common. Nonetheless, the development and commercialization of microorganisms is still a lengthy process.

### Polylactic acid

Polylactic acid (PLA), or polylactide, is an aliphatic polyester made up of repeating lactic units. PLA is an attractive bio-based plastic derived from corn starch, tapioca roots, starch, sugarcane, and sugar beets, which can be metabolized both in vivo and in the environment [459]. PLA has modulus and tensile strength comparable to petroleum-based polymers. Therefore, PLA is attractive to replace conventional synthetic polymers, especially in packaging due to its high transparency. It is also used as insulation foam, for automotive parts, and fibers [162]. Some PLA’s drawbacks are its inherent brittleness, low thermal stability, low impact resistance, and moderate gas barrier properties. PLA also presents a relatively low crystallization rate and is prone to aging at room temperature [460]. While PLA has been proposed as an environmentally friendly alternative to polyolefins, PLA’s lifecycle involves significant energy input and if this energy derives from fossil resources, PLA shows no better carbon balance than polyolefins [461]. Nonetheless, consumer product companies like Newman’s Own, Whole Foods, and Walmart are pushing for the use of PLA for packaging applications [198].

PLA can be synthesized by polycondensation of lactic acid, as shown in Fig. 2, in which high molecular weights are achieved at very high conversions (> 99% conversion for a degree of polymerization of 100). In this reaction, the monofunctional impurities (e.g., ethanol or acetic acid) limit the molecular weights achievable. However, the use of highly pure lactic acid and reduction of the water formed azeotropically during the polycondensation can lead to high molecular weight PLA [461]. Mitsui Toatsu Chemicals (Japan) developed an azeotropic distillation process using a high-boiling-point solvent to drive the removal of water in the direct esterification process to obtain high molecular weight PLA [462]. By contrast, Cargill-Dow, a venture between Dow Chemical and Cargill, produces PLA by ring-opening polymerization (ROP) of the dimeric lactide. As mentioned, Lactide is prepared from lactic acid via linear oligomers as intermediates in the presence of Sn^II^-carboxylates or Sn^II^-alkoxides. Since the polymer properties are strongly dependent upon the stereostructure, impurities such as the meso-lactide are removed from the l-lactide by distillation or crystallization [462]. Finally, PLA high polymer is produced using a tin-catalyzed, ring-opening lactide polymerization in the melt, eliminating the use of costly and environmentally unfriendly solvents [462]. In April 2002, Cargill-Dow (tradename “NatureWorks”) started operation of a PLA plant with a capacity of 140 ktpa in Blair, Nebraska, USA. The plant uses starch isolated from corn as a raw material. The starch is hydrolyzed to glucose and converted to lactic acid for subsequent conversion to PLA. Meanwhile, in Japan, Mitsui produces PLA under the tradename LACEA [461]. Even though PLA downstream processing has improved, its production cost is still more expensive than the fossil alternatives. Moreover, fossil-derived plastics prices have fallen recently following the low crude oil prices, making the economic competitiveness of PLA more challenging [162].

The largest global commercial producer of PLA is the USA-based NatureWorks, originally a joint venture between Cargill and Dow Chemicals, which is now owned by Cargill and PTT Global Chemical [162]. In 2012, NatureWorks created AmberWorks with BioAmber to bring a PLA/polybutylene succinate (PBS) composite to market [213]. NatureWorks produce PLA resins under the Ingeo brand and have a commercial production plant in Nebraska, USA, (150 ktpa). In 2016, NatureWorks introduced Ingeo™ 3D860, a new PLA formulation for 3D printing designed to add impact resistance and heat resistance to finished parts printed with PLA filament [463]. On the other hand, Total and Corbion Purac plan to build a PLA polymerization plant with a capacity of over 75,000 tonnes at Corbion’s site in Thailand [410]. Corbion Purac has also announced a collaboration with Japanese Toyobo to produce Vyloecol, an amorphous PLA product for coating and adhesive applications, for the European market. Moreover, Supla Co., Ltd., will set up a 10 ktpa PLA polymerization factory in China, which will use Corbion Purac’s lactides as raw material [162]. A joint venture between Galactic and Total Petrochemicals, Futerro operates a 1.5 ktpa demonstration plant in Escanaffles, Belgium, which produces various PLAs [162]. Synbra Technology (Netherlands) developed, together with Sulzer Chemtech (USA) and Corbion Purac (Netherlands), a cost-effective polymerization process for high-quality PLA from a biorenewable resource. Applying this technology, Synbra Technology operates a 5 ktpa PLA resin plant in the Netherlands since 2011 and a 1 ktpa PLA pilot plant in Switzerland. Synbra Technology developed a PLA-based particle foam branded BioFoam^®^, which is made from renewable resources and is the world’s first particle foam to receive a Carbon Neutrality verification [464]. Uhde Inventa-Fischer (UIF) (Germany) constructed a pilot plant in 2010 to produce 0.5 ktpa of PLA in Guben, Germany and developed the PLAneo^®^ process for PLA production. After successful lab-scale polymerization, UIF operated a miniplant in Berlin in 2005. Then, in 2011 a 500 tpy pilot plant was built and operated in Guben, Germany. Later, UIF licensed its PLAneo^®^ technology for plants with annual capacities up to 100 ktpy. In June 2016, UIF signed its first contract for a PLA production plant with a capacity of 10 ktpy [464]. Being a LA derivative, PLA produced from lignocellulose depends on the feasible production of LA. Similar to the lactide case, production of PLA from cellulosic biomass is still under development.

### Polytrimethylene terephthalate

Polytrimethylene terephthalate (PTT) can be obtained by transesterification in the melt phase using 1,3-propanediol (1,3-PDO) and terephthalic acid with tetraisopropyl titanate as the catalyst (Fig. 2) [465]. Many polymer’s properties are depend on the odd or even numbers of methylene units in their main chains. Compared with other aromatic even-numbered polyesters, such as polyethylene terephthalate and polybutylene terephthalate, PTT as an odd-numbered polyester shows several advantages, such as good tensile property, resilience, outstanding elastic recovery, colored facility, high birefringence and luminous transmittance, and low dielectric losses at room temperature [466]. PTT is suitable for various applications, such as engineering thermoplastic fibers or film [467]. Therefore, multiple methods have been developed to increase its application: physical blending, copolymerization or transesterification, and hybridization with nanoparticles [465, 467, 468].

DuPont and Shell Oil used 1,3-PDO as a co-feed with purified terephthalic acid (PTA) to produce PTT, which could be used in rug and clothing textile applications as a fiber [199]. PTT was commercially produced by Shell Chemicals (Corterra^®^) and DuPont (Sorona, Biomax^®^ PTT). The production of PTT by Shell oil was based on the 1,3-PDO obtained by the hydroformylation of ethylene oxide. In contrast, DuPont and Tate & Lyle produced F at its plant in Kinston, North Carolina, USA, using 1,3-PDO derivate from renewable sources (corn sugar) [469]. DuPont remains in the PTT business, while Shell Oil exited the businesses for both 1,3-PDO and PTT [199]. In 2013, the Shenghong Group (China) started the construction of a 50 ktpy biological PDO and 20 ktpy PTT plant in Suzhou, Jiangsu, China, and is currently manufacturing 1,3-PDO and PTT [470]. PTT is emerging as an application segment and accounted for 71.8% of the total 1,3-PDO’s market volume in 2014 [470]. As the industry moves toward replacing conventional polyesters with PTT, the demand for 1,3-PDO and PTT will increase. Nonetheless, PTT production from lignocellulosic materials would be possible only if 1,3-PDO is successfully manufactured as part of a biorefinery at low costs.

### Propylene glycol

Propylene glycol (PG), or 1,2-propanediol, is used in the production of unsaturated polyester resins, coolants and antifreeze, hydraulic and brake fluid, aircraft de-icing fluid, heat transfer fluids, paints, and coatings [142]. Furthermore, PG is safe for human consumption, and thus, it is used in the production of fragrances, cosmetics, personal care products, food, flavorings, pet food/animal feed, and pharmaceutical formulations [161]. PG price in northwestern Europe was reported to be around $1530/tonne (2010-2014) [205], and its market is expected to grow at about 4.5% annually [198]. PG can be produced by hydrating propylene oxide (PO), hydrogenolysis of glycerol over mixed-metal catalysts, hydrogenolysis of xylitol or lactic acid (LA), or hydrocracking of sorbitol. Historically, PG has been produced by hydrating propylene oxide. In this process, the hydration reaction occurs in excess of water at 120 °C–190 °C and 21 atm. The resulting stream contains a mixture of mono-, di-, and tri-propylene glycols. Excess water is removed in evaporators and drying towers. Finally, glycols are purified using high vacuum distillation [198]. Due to their similar properties, bio-based PG is a drop-in replacement for conventional PG. Since glucose can be converted to sorbitol through hydrogenation [142, 471, 472], production of PG via hydrocracking of sorbitol could be integrated into a cellulosic ethanol process (Fig. 2). In 2004, Global Biochem and IPCI demonstrated the production of EG, propylene glycol, and other polyols from sorbitol at a pilot plant located in China [178]. An alternative route for producing PG is via hydrogenolysis of xylitol. While reasonable yields of EG (80%) and PG from xylitol have been reported, the main challenge for this process is to identify a low-cost xylose stream or develop a process capable of converting sugar mixtures [163]. Another route to produce PG is via hydrogenation of LA or lactates. This process is based on hydroperoxidation chemistry or chlorohydrin process which involves the use of hypochlorous acid. Introduction of ruthenium on activated carbon as the catalyst for the hydrogenation reaction resulted in 95% conversion of LA and a PG’s selectivity higher than 90% [142].

Glycerol is a by-product in the conversion of fats and oils to fatty acids or fatty acid methyl esters for biodiesel production. Due to the fast growth of the biodiesel industry, glycerol has been overproduced worldwide. As a result, the amount of glycerol produced via fermentation of sugar, hydrogenation of carbohydrates, or hydroperoxidation of LA is not relevant. Both industry and academia are concentrated on the development of routes for conversion of glycerol to other chemicals and polymers [142]. Conversion of glycerol into a family of derivatives, including EG, PG, acetol, and LA via catalytic hydrogenolysis is by far the most successful route to produce PG. In this reaction, glycerol is reduced at 200 psi H_2_ and 200 °C, over a copper chromite catalyst, resulting in PG’s selectivity of nearly 90% at 65% conversion [473].

Dow Chemicals currently operates a PG plant with a capacity of 254 ktpy in Stade, Germany [474]. In 2012, Dow Chemicals established a PG plant in Thailand, where propylene oxide (PO) from the adjacent hydrogen peroxide to propylene oxide (HPPO) facility is converted to PG [475]. In 2012, BASF and Oleon inaugurated a PG unit capable of producing more than 20 ktpy of glycerin-based PG at Ertvelde, Belgium [476]. The Pacific Northwest National Laboratory (PNNL) in Richland, Washington, USA, developed a process using rhenium-promoted catalysts which was later licensed by ADM (Chicago, USA) for scale-up and commercialization [198, 476]. ADM manufactures PG in a 100 ktpy facility (Decatur, Illinois, USA), where glycerol produced during the conversion of vegetable oils to biodiesel is used as raw material [477–479]. In China, Global Biochem manufactures 5.2 ktpy of PG using bio-based sorbitol as feedstock [178]. Due to the overproduction of the glycerol and maturity of the glycerol PG technology, the bio-based PG industry is expected to be based on glycerol. If the bio-PG market is consolidated, co-production of PG from lignocellulose-based sorbitol could be possible; however, it would have to compete with the large and mature glycerol-based PG producers. Industrial production of cellulose-based PG will be only possible if the conversion of lignocellulose-based sugars to sorbitol or xylitol is viable.

### Sorbitol

Sorbitol is a six-carbon sugar alcohol that is largely used as sweetener, thickener, humectant, excipient, and dispersant in food, cosmetic, and toothpaste [142]. Sorbitol is an attractive compound because a wide range of functional derivatives can be produced from it. For example, almost 15% of the world sorbitol is used for the industrial production of vitamin C (ascorbic acid) via fermentation [480, 481]. Through the cyclodehydration of sorbitol, 1,4-sorbitan and 3,6-sorbitan intermediates are generated; afterward, the dehydration of these intermediates yields isosorbide. Other commercial derivatives from sorbitol are PG, EG, glycerol, 1,4-sorbitan, and 2,5-anhydrosugars [163]. More information about the conversion of sorbitol to polymers can be found in the review performed by Isikgor et al. [142] Additionally, pathways for the conversion of sorbitol to gasoline-range alkanes are under development. In a novel process, called aqueous phase hydrodeoxygenation (APHDO), the hydroxyl groups of sorbitol are hydrogenated over a bifunctional metal-acid catalyst (e.g., Pt/SiO_2_–Al_2_O_3_) at 225 °C under the pressure of 3.96 MPa and then dehydrated into unsaturated species, followed by successive hydrogenation and reforming into liquid alkanes (C5–C6) with a selectivity of 58–89% [482, 483]. Further research to develop low-cost catalysts and increase yields is needed to commercialize the production of fuels from sorbitol [482–485].

Sorbitol is manufactured by several companies with a total production volume of 164 ktpy, with a price of $650/tonne, and sales of MM$107 per year [162]. Sorbitol is produced almost exclusively from biomass, mainly from corn, cassava, and wheat. In this process, starch biomass is converted to glucose through enzymatic hydrolysis. Later, the hydrogenation of glucose takes place, as shown in Fig. 2, at 130–150 °C with H_2_ at pressure ranging from 39 to 118 atm in the presence of a nickel or ruthenium catalyst (e.g., Raney nickel) [142, 471, 472]. The commercial process is based on batch technology which allows the complete conversion of glucose and ensures the fulfillment of the strict food industry requirements [163]. Since the commercial production of sorbitol is widely practiced and demonstrated yields are about 99%, essentially no technical development is needed [142]. Nonetheless, a future opportunity could be the development of a continuous process that achieves similar high conversion [486]. Alternative processes to produce sorbitol have been explored, for example, Silveria et al. [487] fermented fructose and glucose to sorbitol and gluconic acid in nearly quantitative yields using *Zymomonas mobilis*. The authors concluded that biotechnological production of sorbitol may be economically possible in at least some countries with low price biomass, such as sugarcane. Ladero et al. [488] reported that resting cells of an engineered *Lactobacillus plantarum* produced sorbitol from glucose with high efficiency (61 to 65% conversion), closed to the maximal theoretical (67%). Despite the efforts, fermentative routes are unlikely to replace in the short term the technically mature catalytic hydrogenation process, unless efficient organisms capable of ferment mixtures of sugars, in short times, are developed. Another approach, denominated one-pot conversion, involves the direct production of sorbitol from cellulose. Fukuoka and Dhepe [489] tested Pt or Ru catalysts under hydrogenolysis conditions in water to produce sorbitol from cellulose. The catalyst Pt/γ-Al_2_O_3_ resulted in the highest yield (25% to sorbitol and 6% to mannitol) at 190 °C and 49 atm H_2_ [489]. The authors proposed that cellulose is hydrolyzed by in situ generated acid sites to form glucose, which is then immediately reduced to sorbitol over the metal catalyst. Another pathway to generate polyols from cellulose involves a two-step process where the reaction takes place in hot water using Ru/C catalyst. The high temperature and pressure (245 °C and 59 atm H_2_) generate H+ from water, which, in a second step, hydrolyzes cellulose. The reaction achieved a 29.6% yield of sorbitol [490]. A recent approach involves the use of ionic liquids (ILs) which completely dissolves cellulose, facilitating the hydrolytic depolymerization [106]. By using a heterogeneous Pt or Rh catalyst with a homogeneous Ru complex in 1-butyl-3-methylimidazolium chloride under H_2_ pressure, Ignatyev et al. [491] achieved full cellulose conversion and 51% − 74% selectivity to sorbitol. While promising, the use of ILs is challenging due to ILs’ high boiling points and the fact that they decompose at elevated temperatures, hindering separation by distillation. Thus, the use of ILs is limited by the development of an efficient separation system. Another downside of ILs is their corrosive nature against the commonly used stainless steel equipment. Therefore, the one-pot technology needs further research to be integrated into a cellulosic ethanol process.

Roquette Freres is the largest sorbitol producer that, together with Cargill and Ingredion Incorporated, generates over 70% of the total market volume of sorbitol [142]. Since the 1950s, Roquette Freres has been producing sorbitol, under the trade name of NEOSORB^®^Sorbitol, on a large scale. Roquette Freres operates sorbitol plants in Illinois, USA; LianYun-Gang, China; and Ulsan, Korea [492]. Through research, Roquette identified new applications for sorbitol, such as humectant in cosmetology and as a sugar substitute in non-cariogenic confectionery. Recently, Roquette Freres has focused on the commercialization of sorbitol’s derivatives, such as POLYSORB^®^ isosorbide [493]. Cargill also produces sorbitol using the catalytic hydrogenation of maize-based glucose. Sorbitol is offered by Cargill as a free-flowing crystalline powder and as aqueous solutions for food and pharmaceutical applications [494]. Cargill produces sorbitol at various facilities: Castelmassa, Italy; Krefeld, Germany; Blair, Cedar Rapids, Dayton, Eddyville, Memphis, and Wahpeton, USA; and Martorell, Spain [495]. Ingredion Incorporated produces sorbitol at its facility in Mapleton, Winston-Salem, North Carolina, and Stockton, USA; Guadalajara, Mexico City, and San Juan del Rio, Mexico; as well as eleven plants in south America: Argentina, Brazil, Chile, Colombia and Peru [495]. While the process to convert glucose to sorbitol is mature and may offer economic benefits to a cellulosic biorefinery, its integration into a cellulosic ethanol process faces some challenges. Sorbitol could be produced from hydrolyzed glucose and/or unfermented glucose. In the cellulosic ethanol process, these streams contain multiple sugars (xylose, arabinose, galactose, mannose), oligomers, furfurals, lignin, proteins, enzymes, carbohydrates (e.g., cellulose, xylan), organic compounds, carbon dioxide, and microorganisms that may have a detrimental impact on the catalyst. Therefore, a series of separation/purification processes such as hydro-cyclone systems, filter presses, or ion exchange columns may be needed to be implemented [496]. The separation/purification equipment required would depend on the kind of pretreatment, enzymes, and fermentation microorganism used, as well as on the purity of the sugar stream entering the hydrogenation reactor. Since the individual hydrogenation of sugars has been studied and modeled in the past (xylose [497, 498], arabinose [499, 500], galactose [499], and mannose) [501], sugars may be isolated and hydrogenated. To implement this option, economic analyses are required to determine the most efficient and low-cost separation technologies. Alternatively, the mixture of hydrolyzed sugars could be hydrogenated in a single step. However, it is noteworthy that very few studies have explored the hydrogenation of sugar mixtures [499, 500, 502]. For example, Wisniak and Simon [502] reported that mixtures of glucose–fructose hydrogenate at a slower rate than each reagent alone. Thus, further research is needed to understand the impact of sugars ratio on the hydrogenation reaction rates and selectivity, as well as to select optimal conditions and catalyst type to maximize yields.

### Squalene

Squalene is produced in human skin and has excellent moisturizing properties. It has wide applications in the manufacture of fine chemicals, magnetic tape, and low-temperature lubricants, as well as an antistatic agent and emollient in cosmetics and pharmaceuticals. It is also used as an additive in animal feed. A 500 mg capsule of squalene was reported to cost 0.125 US$ in 2001 [503]. Squalene market size was estimated at 2400 tons in 2014, as per the latest research report by Global Market Insights, Inc [504].

Traditionally, shark liver oil was used to produce squalene. Since deep-sea sharks have large reserves of squalene, these sharks were caught specifically for their liver oil. With the introduction of deep-sea shark fishing quotas for the Northeast Atlantic in 2006, squalene prices increased, and shark-based squalene became 20–30% more expensive than plant-based squalene. As a result, Unilever and L’Oreal replaced the use of shark-based squalene with a plant-based version [505]. The first plant-based precursor dedicated to sterol biosynthesis, in the isoprenoid pathway, is squalene. At standard conditions, squalene is consumed for the synthesis of ergosterol, however; its levels in the yeast *Saccharomyces cerevisiae* are elevated in hypoxia or heme deficiency [506]. The pathway of squalene production in *Saccharomyces* spp. starts with the synthesis of mevalonate from acetate, and then mevalonate is converted to two activated isoprenes. Later, condensation of six activated isoprene units forms squalene and finally conversion of squalene to the 4-ring lanosterol [503]. Mantzouridou et al. [507] reported the accumulation of squalene in yeast cells under semianaerobic conditions (1.6 mg/g dry biomass), which was 40 times higher than that reported under anaerobic conditions (0.04 mg/g dry biomass). Other microorganisms have been reported to produce similar squalene accumulation size. Fan et al. [508] reported an accumulation of squalene of 0.53 mg/g dry biomass using the *Aurantiochytrium mangrovei* FB3 through medium optimization and the treatment with terbinafine, an inhibitor of squalene monooxygenase in the sterol biosynthetic pathway. Yue et al. [509] reported a maximum squalene content of 1.17 mg/g dry biomass reached during the next 3 h after methyl jasmonate treatment (12.32 g/L) at 48 h of cultivation using the microalga *Schizochytrium mangrovei*. A larger squalene production (70.32 mg/g dry biomass) was reported by Chang et al. [510] using an oleaginous yeast strain, *Pseudozyma* sp. JCC 207, grown under microaerobic conditions. Since the *Pseudozyma* species is not included in the qualified presumption of safety, a list published by the European Food Safety Authority, the optimization of squalene production is expected to focus on *S. cerevisiae* [511].

Amyris Inc. manufactures and sells commercial quantities of squalene to cosmetic ingredient buyer Soliance (France). Amyris uses an engineered microbe in its fermentation facility in Brazil to produce farnesene and by-products such as squalene from up to two million tons of crushed sugarcane per year [512]. In 2012, Amyris started operations at its plant in Brotas, Brazil, supplying 10% of the global squalane market. Amyris increased the plant production volume in 2013, covering 18% of the market [513]. Amyris has selected Dowell C&I Co., Ltd., a supplier of ingredients for the personal care industry, as its exclusive distributor of Neossance™ Squalane in the Republic of Korea [512]. One of the main barriers in the production of squalene is the low production yield [511]. As a result, margins are compromised and further process optimization is required. The economic viability of producing squalene from lignocellulosic glucose may be compromised by the potential inhibition of the pretreatment by-products on the microorganisms, and thus, the cost of the separation–purification equipment that may be required to generate a clean glucose stream from the hydrolyzed stream (Fig. 2). Since squalene has a high price per volume, only a small fraction of the hydrolyzed sugars would be required for its production and to increase the gains of the venture. Thus, economic analyses are needed to balance the expenses on separation and purification costs and the gains of selling squalene.

### Succinic acid

Succinic acid (SA) applications range from high-value niche applications such as personal care products and food additives to large-volume applications such as biopolymers, plasticizers, polyurethanes, resins, and coatings [162]. In 2012, fossil-based succinic acid production was approximated in 40 ktpy with a market value of $100 million. Bio-based succinic acid has a current market price of approximately $2860/tonne, while the fossil-based equivalent is valued at around $2500/tonne. As a platform chemical, SA’s market is expected to increase to a size of > 700 ktpy by 2020 [514]. SA is also a precursor of numerous chemicals such as adipic acid (a precursor for Nylon X), 1,4-BDO (a precursor for polyesters and Spandex), tetrahydrofuran (an important solvent and a precursor for poly[tetramethylene ether] glycol), *N*-methylpyrrolidone (NMP, an important solvent in chemical and lithium-ion battery industries), 2-pyrrolidone (a precursor for pharmaceuticals and vinylpyrrolidone), and other green solvents and chemicals [515].

Fossil-based succinic acid has predominantly been produced through the hydrogenation of maleic anhydride or maleic acid. Maleic anhydride and maleic acid are manufactured from the oxidation of n-butane, a direct product from petroleum refining or natural gas processing [198]. Current petroleum-based SA manufacturers include the Dutch company DSM; Israel-based Gadiv Petrochemical Industries; Japanese companies Mitsubishi Chemical Corp., Kawasaki Kasei Chemicals, and Nippon Shokubai; numerous producers in China including Anqing Hexing Chemical and Anhui Sunsing Chemicals; and smaller producers in India [516]. Bio-based succinic acid is produced through low pH yeast or bacterial fermentation of biomass-derived intermediates including commodity sugars, glycerol, and lignocellulosic sugars (Fig. 2). Commodity sugars are the feedstock for the current and planned commercial-scale processes. Microorganisms for the production of succinic acid have been studied, e.g., rumen bacteria such as *Actinobacillus succinogenes*, *Mannheimia succiniciproducens, Basfia succiniciproducens*, and other microorganisms such as *E. coli, Anaerobiospirillum succiniciproducens, C. glutamicum*, and *S. cerevisiae* [514]. More information on the use of metabolically engineered microorganisms for the production of SA can be found in a recent review performed by Ahn and co-workers [515]. Commonly used feedstocks are refined sugars (sucrose, glucose, and fructose), starch, sugar beet, and cane molasses. Due to their low cost, recent efforts are focused on the use of second-generation feedstocks, such as waste streams from agriculture, forestry, and paper milling. A major disadvantage of this strategy is the required process to release sugars from lignocellulose, as well as the impurities and sugar degradation products generated during pretreatment and hydrolysis, which can act as inhibitors [517]. Using corn stalk enzymatic hydrolysate as a substrate and *E. coli*, Hodge et al. [518] produced a concentration of 57.8 g/L of SA. In contrast, a lower SA concentration (42.2 g/L) was obtained when softwood hydrolysate was used as the substrate. Therefore, hydrolyzed streams need to be treated accordingly to reduce the impact of inhibitors in the fermentation stage. On the other hand, obstacles in the recovery and purification of SA are being solved using downstream processes such as vacuum distillation, single reactive extraction, and crystallization, in which purity as high as 99.5% is achievable [519].

Several companies have started the large-scale fermentative production of SA applying different producing strains. In 2008, BioAmber, a joint venture between US-based DNP Green Technology and the French research consortium Agro-Industrie Recherches et Développements, developed a SA plant in Pomacle, France, with an annual capacity of 2000 tonnes [198]. They constructed a 30 ktpa plant (with 20 ktpa expansion plans) in Sarnia, Canada, with joint venture partner Mitsui & Co [213]. BioAmber has developed licensing agreements with Cargill to adopt a yeast microorganism that is tolerant to low pH environments and able to utilize a range of lignocellulosic feedstocks (Fig. 2) [218]. Recently, BioAmber reported that sales of bio-SA in the first quarter of 2017 increased 46% when compared to the same quarter of 2016, reaching $2.1 million. The company is planning to build a second BioAmber facility in North America. The decision on whether locate the second plant in the USA or Canada is expected to be made during the third quarter of 2017 [520]. Reverdia, a joint venture between Roquette and DSM, was established in 2008. In 2012, the company started a 10 ktpy SA production facility in Cassano Spinola, Italy. The facility uses a *S. cerevisiae* yeast strain, Biosuccinium™, which is tolerant to low pH fermentation [198, 218]. The company reported that this process has reduced GHG emissions because it generates very little waste [521]. BASF/Corbion-Purac joint venture, Succinity, isolated a new member of the family *Pasteurellaceae* from bovine rumen and named it *Basfia succiniproducens*. This natural producer has a high yield of 0.75 mol of SA per mol of glucose and has been optimized through metabolic flux analysis and subsequent metabolic engineering. A 10 ktpy facility came online in mid-2014 at the Corbion Purac site in Montmeló, Spain [198]. Succinity has plans for a second large-scale 50 ktpa facility, the final investment decision for which will be made following a successful market introduction of the Montmelo plant [162]. Myriant, which is not a joint venture, partnered with ThyssenKrupp Uhde to commercialize bio-SA. In 2013, the partnered companies scaled and produced succinic acid from commodity sugar feedstocks at the ThyssenKrupp Uhde site in Leuna, Germany. Myriant utilizes a genetically modified *E. coli* for succinic acid production. This strain is reportedly utilized in the 14 ktpy SA facility that came online in Lake Providence, Louisiana, USA, in June 2013 [162]. The company reported plans to expand the capacity of this facility to 77 ktpy by late 2015. In agreement with China National BlueStar, Myriant agreed to scale up a 100 ktpy SA facility in Nanjing, China, but a targeted date has not been announced [198]. The advances in efficient downstream process, reduced feedstock cost, reduced GHG emissions, and the large potential market size have raised hopes for higher earnings and profits in SA production in the future. As a result, an active research is currently being performed to develop an efficient and inhibitor-resistant strain that can generate SA from lignocellulose-derived sugars. The possibility of an integrated biorefinery, producing 2G ethanol and SA is within reach with a proper optimization.

### Terpenes

Terpenes are responsible for the pleasant smell and pharmacological activities of conifer wood, palm trees, citrus fruits, eucalyptus, lemongrass, lilies, peppermint species, and many other plants or parts of those. Terpenes are extracted or steam distilled. These extracts and steam distillates, known as ethereal or essential oils are used to create fine perfumes, to refine the flavor and the aroma of food and drinks, and to produce medicines of plant origin (phytopharmaca) [522]. Terpenes and terpene derivatives represent a $650 million global market, according to Allylix [523]. Terpenes structure follows a general principle: 2-methylbutane residues build up their carbon skeleton [522]. About 30,000 terpenes are known in the literature, some examples of terpenes include isoprene (C5 hemiterpene), farnesene (C15 Sesquiterpenes), artemisinin (C15 sesquiterpenes), citral (C10 monoterpenes), carotenoids (C40 tetraterpenes), menthol (C10 monoterpenes), Camphor (C10 monoterpenes), and cannabinoids [524].

Terpenes can be manufactured from petrochemical sources and from terpene feedstocks. For example, isoprene is produced as a by-product of naphtha or oil cracking in the production of ethylene. Terpenes are also extracted in small quantities from natural sources. The downside of these methods is that they are expensive and non-environmental-friendly [524]. Given the demand for terpenes, more economical and eco-friendly methods to produce terpenes are needed. However, these methods must use inexpensive and non-toxic feedstocks [525]. Therefore, microbial fermentation is a potential alternative for the production of terpenes. Terpenes are involved in bacterial cell wall biosynthesis, and they are produced by some trees. Nonetheless, not all bacteria can produce terpenes and/or their precursors as metabolic products [524]. As a result, microorganisms that comprise one or more polynucleotides coding for enzymes in a pathway that catalyze the conversion of a carbon source to one or more terpenes have been developed. Chen and co-workers [524] developed a carboxydotrophic acetogenic recombinant microorganism capable of producing one or more terpenes and/or precursors thereof and optionally one or more products by fermentation of a substrate comprising CO (any gas containing a level of carbon monoxide). Garcez Lopes and Slovic [525] patented a genetically modified microorganism able to ferment a carbon source (sugarcane juice, hydrolyzed starch, hydrolyzed lignocellulosic materials, glucose, sucrose, fructose, or glycerol in any form or mixture thereof) to a terpene, such as isoprene and/or farnesene, and co-product [525]. The authors reported a ratio of grams of the produced isoprene and a co-product to grams of the fermentable carbon source of 0.01–0.98.

Founded in 2005, Allylix has developed a fermentation-based technology platform derived from glucose to produce renewable specialty chemicals, primarily terpenes, and their derivatives. Allylix (San Diego, USA) focused on the gene cloning, metabolic engineering, protein engineering, fermentation development, and purification methods for manufacturing organic chemicals. Allylix commercially produced terpene-based flavors and fragrances: nootkatone, a grapefruit fragrance, and valencene, an orange fragrance. Moreover, other low-cost terpene-based compounds are planned for commercialization, e.g., Epivone (epi-beta-vetivone) for use in fragrance applications [523]. By 2014, Evolva (Switzerland) acquired Allylix, with plans to market the compounds nootkatone and valencene [526]. Since over 1 million kilos of oranges are needed to extract 1 kilo of valencene, production of valencene via fermentation provides a more sustainable, economical, and reliable supply chain [526]. The Evolva’s most valuable asset is nootkatone, which has potential in the > $1 billion insect-repellent market [527]. Isobionics (Netherlands) has also developed yeast strains capable of producing nootkatone and valencene, and signed a distribution agreement with DSM in 2014 [528]. At this stage, it is not possible to know if Evolva or Isobionics has a cost advantage, but Isobionics/DSM might be blocked from the insect repellent market, since Allylix filed in August 2013 a worldwide patent [527]. Patented engineered modified microorganisms were reported to be capable of fermenting lignocellulosic-derived sugars. However, details on their efficiency or inhibitors resistance were not reported. Since public literature on the fermentative production of terpenes is limited, and successfully engineered microorganisms are patented, it is difficult to draw a conclusion on the potential manufacture of terpenes from lignocellulose. However, production of terpenes from glucose is already available; thus, if glucose can be separated from the hydrolysate stream, terpenes can be produced from lignocellulose as shown in Fig. 2.

### Xylitol

A sugar alcohol, or polyol, xylitol is an attractive diabetic sweetener which has similar taste to sucrose but contains 40% fewer calories. Xylitol has applications in the food (confectioneries and chewing gums), odontological (anticariogenicity, tooth rehardening, and remineralization), and pharmaceutical sectors [162]. In 2015, xylitol market was estimated at $624 million per year with a price of $3900 per tonne [162].

**Fig. 6:**
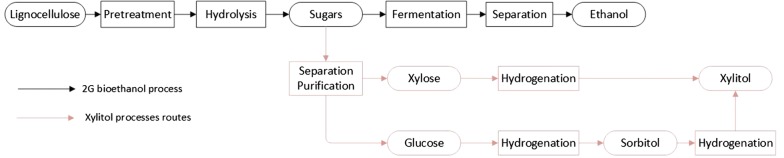
Process diagram for the production of 2G ethanol and integrated process options for the production of xylitol from (red line): xylose and glucose

The traditional production of xylitol involves direct chemical hydrogenation of xylose derived from xylan over a catalyst such as nickel, ruthenium, and rhodium at high pressure and temperature, as shown in Fig. 6, process A [529]. Yadav et al. [498] evaluated the activity of a Ru catalyst on a NiO-modified TiO_2_ support, Ru/(NiO–TiO_2_), in the liquid-phase catalytic hydrogenation of xylose to xylitol. The effect of NiO additive in the catalyst Ru/(NiO–TiO_2_) was reported to enhance the conversion, yield, and selectivity to xylitol. At high temperature (140 °C), the conversion of xylose was increased to an optimum level, but xylose to xylitol selectivity decreased due to the formation of by-products. In contrast, the hydrogenation of hydrolysate sugars from sugarcane bagasse using a ruthenium (Ru 2%/C) catalyst at mild conditions (80 °C and 20 atm), resulted in the transformation of 87% of monosaccharides into polyols, and 91% of the total xylose into xylitol (high selectivity, 98%), after 3 h [530]. There is no major technical barrier associated with the production of xylitol via hydrogenation [163]. The main bottleneck for conversion of lignocellulosic biomass to xylitol is the lack of efficient and low cost technologies for conversion of biomass into pentose sugars, for example, the high costs of pretreatment, separation, and purification processes, as well as expensive alkaline/acid catalysts and corrosive-resistant equipment. Other barriers include the degradation of hemicellulosic components and the generation of inhibitory/toxic compounds [52].

Alternatively, xylan can be hydrolyzed into xylose and fermented into xylitol. Xylan consists of the main chain of xylopyranosyl residues linked by b-1,4-glycosidic bonds. Its enzymatic hydrolysis mainly requires *endo*-b-1,4-xylanase (EC 3.2.1.8) that cleaves b-1,4-glycosidic bonds to produce xylooligosaccharides and b-xylosidase (EC 3.2.1.37), which, in turn, cleaves small xylooligosaccharides to produce xylose [531]. Several microorganisms have been developed to convert xylose to xylitol. Misra and co-workers [532] adapted a strain of *C. tropicalis* to produce xylitol from detoxified corncob hemicellulosic hydrolysate. The results showed that the strain resulted in a 1.22-fold increase in xylitol yield and a 1.70-fold enhancement in volumetric productivity, compared to the parent *C. tropicalis* strain. Lignocellulose is attractive as a feedstock due to its low cost, abundance, and the fact that it is a renewable material. To lower the production cost of xylitol, separation and detoxification processes must be optimized or avoidED. For example, detoxification by ion-exchange resins or activated charcoal adsorption removed totally or partly inhibitors, such as furfural and 5-hydroxymethylfurfural (HMF). However, detoxification introduces sugar losses (5 to 10%) and increases production costs. Thus, Ping et al. [533] evaluated the fermentative production of xylitol from detoxified and non-detoxified corncob hemicellulose acid hydrolysate by *Candida tropicalis* CCTCC M2012462. The authors reported that xylitol production with dilute acid hydrolysate medium did not seem to influence specific xylose reductase activity, the main enzyme in charge of converting xylose to xylitol. Nonetheless, there was a decrease in xylitol productivity with non-detoxified hydrolysate, which was attributed to the lower biomass concentration and lag-phase time. It appears that furfural and HMF, produced during pretreatment, have a major detrimental effect on the specific cell growth rate and biomass formation. The authors concluded that the biomass growth rate is essential for xylitol production, and recommended the development of yeast with tolerance to the inhibitory compounds present in the hydrolysates. To reduce the number of stages and operating equipment, Guo et al. [529] constructed a novel *C. tropicalis* for the direct conversion of corncob xylan to xylitol. The b-1,4-xylanase gene (atn) and b-xylosidase gene (atl) from *Aspergillus terreus* were used to engineere *C. tropicalis*/pAUR-atn-atl-3 (*C. tropicalis* PNL3) capable of co-expressing *endo*-b-1,4-xylanase and b-xylosidase. The xylitol yields obtained using the *C. tropicalis* PNL3 to ferment xylan and corncob hydrolysate were 77.1% and 66.9%, respectively. Therefore, the integrated pathway of xylitol production appears to be feasible and efficient. However, technological bottlenecks exist in the fermentability of lignocellulosic streams, for example, the costly separation of xylitol from the fermentation broth, detoxification process, and low conversion yields [162].

In the mid-1950s, Roquette (France) developed a process for manufacturing xylitol. In this process, starches are hydrolyzed to glucose and subsequently hydrogenated to sorbitol which is then transformed to xylitol, maltitol, mannitol, and arabinitol (Fig. 6, process B) [530]. Xylitol is currently manufactured and sold by Roquette under the name of XYLISORB^®^. Roquette has patented a new process for the production of xylitol, which comprises two reaction steps: the fermentative conversion of a hexose to a pentitol and the catalytic chemical isomerization of the pentitol to xylitol [534]. In China, multiple companies have reported to manufacture xylitol from corncobs: Futaste Pharmaceutical Co. (35 ktpy), Jining Hengda Green Engineering Co. (5 ktpy), Hangzhou Shouxing Biotechnology Co. (15 ktpy), Shandong Biobridge Technology Co. (6 ktpy), Tangyin Hung Industrial Co. (2 ktpy), Thomson Biotech Pte. (10 ktpy), Yucheng Lujian Biological Technology Co. (16 ktpy), Zhejiang Huakang Enterprise Co. (20 ktpy), and Shijiang Acid Chemical Co. (10 ktpy) [530]. DuPont produces 2 ktpy of xylitol (branded as XIVIATM) via the DuPont Wood Based (DWB) process at its facility in Thomson, USA [530] [535]. DuPont claimed that the carbon footprint of DWB xylitol is 90% lower than the xylitol produced by the biomass hydrolysis process (BHP), which is the conventional industry standard used by many of DuPont’s competitors [536]. In the BHP process, corncobs are acid hydrolyzed to generate a hemicellulosic hydrolysate, consisting of solubilized sugars like glucose, xylose, and arabinose. Xylose is extracted using a purification and filtration processes. Xylose is then converted to xylitol via hydrogenation, Fig. 6, process A. Finally, xylitol is recovered by separation and evaporation to yield crystallized xylitol. In the DWB process, the xylose facility is integrated with a pulp and paper plant. Pulp and paper plants produce a waste side stream, consisting of black liquor that is usually combusted to generate heat and electricity. Alternatively, the DWB process uses these side streams to generate xylitol. Since the xylose in this feedstock is already hydrolyzed, the DWB process does not require a hydrolysis process. The remaining side stream with reduced xylose and energy content is then incinerated for energy production. The DWB method was reported to require significantly less energy (85% lower), and has less impact on toxicity for both land (94% less) and water (99% less), and has less impact on ozone layer depletion (86% less) than the BHP.

Integration of the 2G bioethanol and xylitol process seems to be technically viable because the sugars derived from lignocellulose can be hydrogenated to xylitol. Hydrolyzed xylose can be hydrogenated to xylitol, while hexose sugars could be used to produce sorbitol, which may be converted to polyols. As mentioned earlier, the barriers to viable production of lignocellulosic xylitol are the high cost of the lignocellulose to sugars conversion and the separation–purification of sugars and products. In 2016, S2G BioChem, a Canadian developer of natural biotechnology conversion processes, announced that it has entered into a license and collaboration agreement with Mondelēz International, a world leader in biscuits, chocolate, candy, and powdered beverages, to help commercialize a sustainably sourced supply of xylitol. S2G will receive financial support to develop the first commercial facility for cellulosic biomass-based xylitol. S2G’s technology can use hard and softwoods, sugarcane, and bagasse, along with non-wood sources like wheat straw as the feedstock, Fig. 6 [537]. Therefore, despite the mentioned barriers, the commercial production of lignocellulosic xylitol is a promising technology that could be a reality, particularly as part of a cellulosic biorefinery.

## Conclusions

Publications on the production of valuable bioproducts have reviewed dozens of promising bio-based chemicals and materials that are on the bench, pilot plant, demonstration, or industrial stage. While most of the reported biochemicals are on the bench and pilot plant stage, some compounds have passed the demonstration stage, called “valley of death,” reaching the commercial production. 2G bioethanol is a promising biofuel that has reached the demonstration stage. However, the production cost of 2G bioethanol needs to be reduced to compete commercially with fossil fuels. This can be achieved by producing valuable materials and/or chemicals alongside 2G bioethanol. Thus, commercial technologies to produce biochemicals from sugars and ethanol can be implemented into the cellulosic ethanol process. While technologies for the production of biochemicals have been successfully industrialized, the production of some of these biochemicals has stopped due to their non-competitive prices against fossil-based chemicals. Since the high cost of sugar- and starch-based feedstock has a major detrimental effect on the production cost of biochemicals, the use of low-cost cellulosic biomass as feedstock is being developed. Therefore, the production of biochemicals using cellulose-based sugars or ethanol can improve the biochemicals and bioethanol’s commercial competitiveness.

Sugars produced during the pretreatment of biomass and enzymatic hydrolysis of pretreated biomass can be converted into a wide range of chemicals such as 1,3-propanediol, acetone, *n*-butanol, itaconic acid, or xylitol. Thus, a portion of the hydrolysate stream generated in the 2G bioethanol process can be used to manufacture biochemicals. However, two major barriers impede the immediate integration of the sugar-to-biochemicals technologies into the 2G bioethanol process. The first is the presence of by-products, generated during the pretreatment stage, which has a detrimental effect on the microorganisms and catalysts used to convert sugars to chemicals. Therefore, separation and purification technologies that remove detrimental compounds must be optimized and introduced into the 2G bioethanol process to generate a relatively pure sugars stream. However, the development of such technologies requires extensive research and their implementation will increase the capital and operating costs. Another option to reduce the negative effect of by-products on the 2G bioethanol process is developing inhibitor-resistant microorganisms and/or catalysts. Nonetheless, successful development and implementation of such microorganisms and catalysts may not be immediate. The second barrier is related to the fact that commercial technologies were developed to convert starch/molasses derivate sugar streams, high in glucose concentration, to chemicals. Most of the commercialized technologies are not designed to manage sugar mixtures, such as the cellulosic hydrolysate which contains pentose and hexose sugars. As a result, the design of microorganisms and catalysts capable of converting multiple sugars is needed to maximize production yields. An alternative approach to co-producing sugar-based chemicals and cellulosic ethanol is integrating the 1G and 2G bioethanol processes. In this arrangement, cellulosic ethanol could cover the ethanol demand, while starch/molasses derivate sugars could be used to produce chemicals by applying available technologies.

First-generation bioethanol is currently used to manufacture various compounds such as ethyl acetate, ethylene, ethylene glycol, or acetic acid. Since the ethanol stream produced in the 2G ethanol process has no major impurities, commercialized technologies for the conversion of ethanol to biochemicals can be immediately integrated. By using a portion of the produced 2G ethanol to manufacture valuable chemicals, the 2G ethanol process can easily adapt to market changes by adjusting production rates. Since the 2G ethanol process is in the demonstration stage, the conversion of cellulosic ethanol to chemicals can add value to lignocellulose and increase the 2G bioethanol feasibility. Technologies for the conversion of ethanol to chemicals are considered mature and can be immediately applied. While techno-economic analyses are needed to determine the viability of producing chemicals from cellulosic ethanol, market analyses are crucial to assessing the market value and size of the selected biochemicals.

Production of microfibrillated cellulose represents a unique opportunity for the 2G bioethanol industry because the solid residue produced after the hydrolysis of pretreated lignocellulose, which is normally burned to produce steam and electricity, can be converted into a more valuable component. Microfibrillated cellulose has applications in polymer reinforcement, and thus, its market is expected to grow in the next decade. Since the production process of microfibrillated cellulose has been successfully scaled up, it can be immediately integrated into the 2G bioethanol process. Co-production of microfibrillated cellulose is an attractive opportunity to increase the 2G bioethanol’s profitability, especially considering the microfibrillated cellulose’s large number of potential applications and market value.

The technologies reviewed in this work have been scaled up to industrial scale and can be integrated into a cellulosic ethanol process with adjustments. While the production of biochemicals from cellulosic derivate sugars requires the development of pathways to process the sugars present in the hydrolysate and to reduce or eliminate the impact of inhibitors, the production of biochemicals from cellulosic ethanol confronts fewer challenges and has room for improvement. Techno-economic analyses are needed to define the feasibility of integrating the mentioned technologies into a cellulosic ethanol plant. However, since most of the reviewed technologies are patented or belong to the private sector, access to their characteristics and specifications is challenging. Moreover, market analyses are vital to estimate the economic competitiveness of cellulosic-based biochemicals against fossil-based chemicals, especially considering the current low oil prices. The reviewed technologies are promising opportunities that could add value to a cellulosic-based biorefinery and boost the commercialization of cellulosic ethanol.

## Data Availability

Not applicable.
